# Supplementary figures and images for: Extended-Synaptotagmin-1 and -2 control T cell signaling and function (part 1 of 2)
Source: EMBO Rep. 2023 Dec 19;25(1):286–303. doi: 10.1038/s44319-023-00011-7 (PMC10897422; doi:10.1038/s44319-023-00011-7)

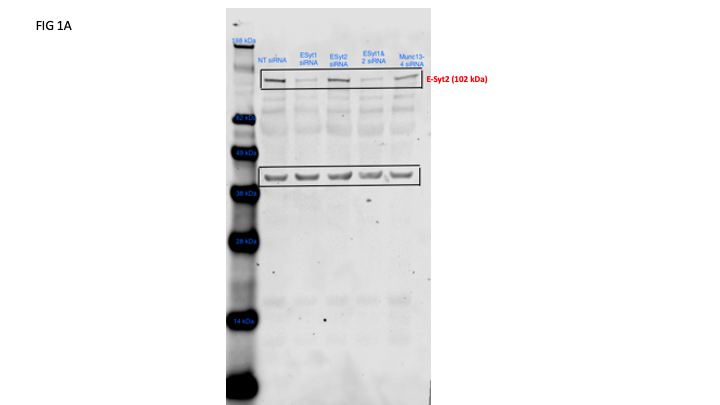

Supplement: Supplementary file 4 — Source Data Fig. 1 [file 44319_2023_11_MOESM4_ESM.zip › FIG 1/Figure 1A /Fig 1A1.png]

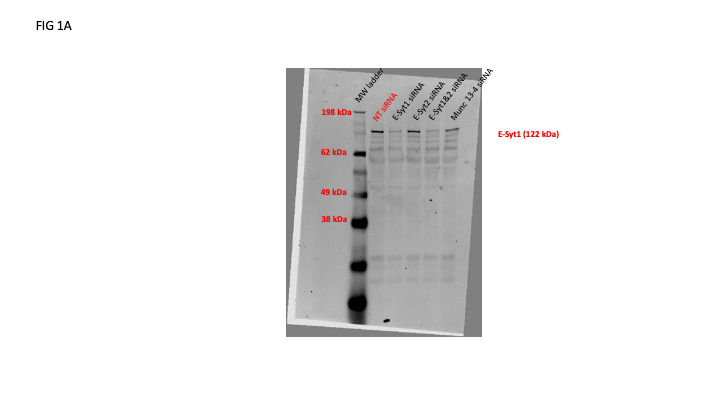

Supplement: Supplementary file 4 — Source Data Fig. 1 [file 44319_2023_11_MOESM4_ESM.zip › FIG 1/Figure 1A /Fig 1A2.png]

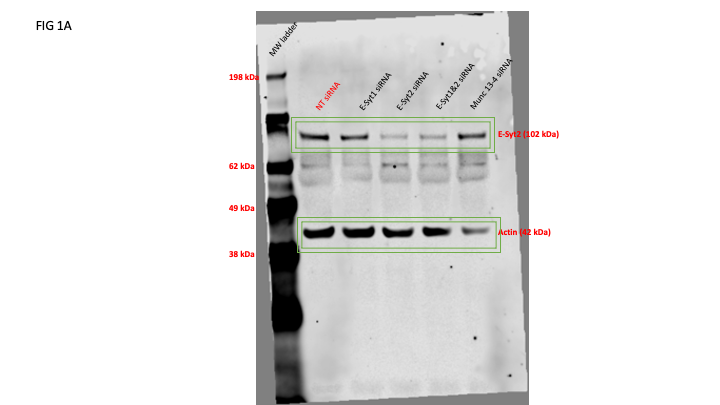

Supplement: Supplementary file 4 — Source Data Fig. 1 [file 44319_2023_11_MOESM4_ESM.zip › FIG 1/Figure 1A /Fig 1A3.png]

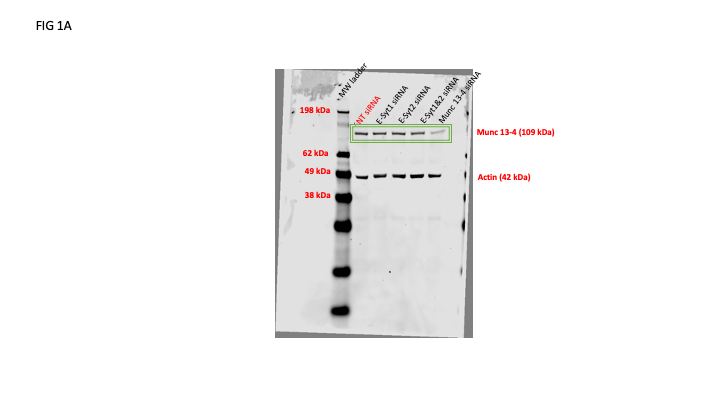

Supplement: Supplementary file 4 — Source Data Fig. 1 [file 44319_2023_11_MOESM4_ESM.zip › FIG 1/Figure 1A /Fig 1A4.png]

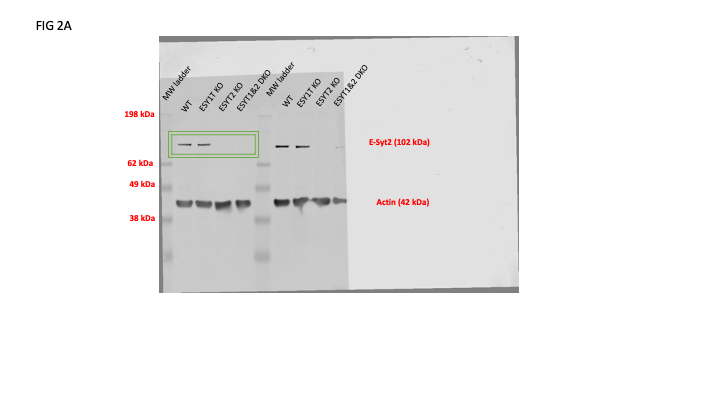

Supplement: Supplementary file 5 — Source Data Fig. 2 [file 44319_2023_11_MOESM5_ESM.zip › FIG 2/Fig 2A1.png]

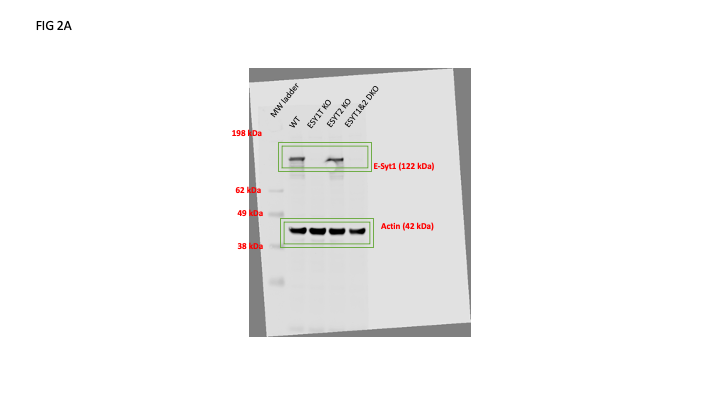

Supplement: Supplementary file 5 — Source Data Fig. 2 [file 44319_2023_11_MOESM5_ESM.zip › FIG 2/Fig 2A2.png]

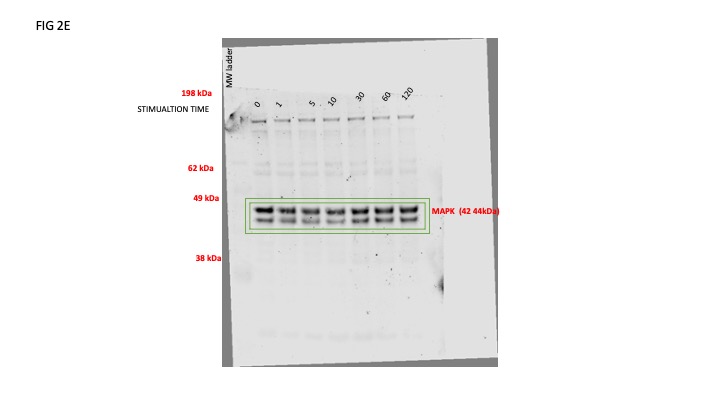

Supplement: Supplementary file 5 — Source Data Fig. 2 [file 44319_2023_11_MOESM5_ESM.zip › FIG 2/Fig 2E2.jpeg]

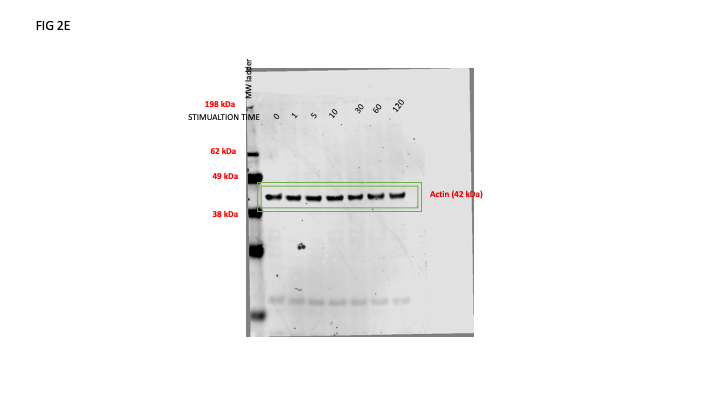

Supplement: Supplementary file 5 — Source Data Fig. 2 [file 44319_2023_11_MOESM5_ESM.zip › FIG 2/Fig 2E3.png]

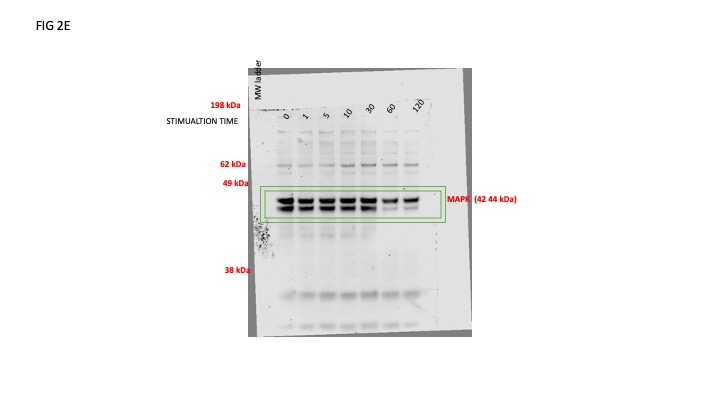

Supplement: Supplementary file 5 — Source Data Fig. 2 [file 44319_2023_11_MOESM5_ESM.zip › FIG 2/Fig 2E5.jpeg]

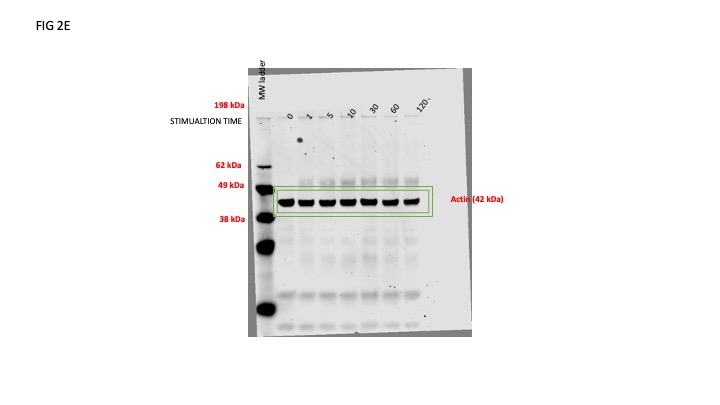

Supplement: Supplementary file 5 — Source Data Fig. 2 [file 44319_2023_11_MOESM5_ESM.zip › FIG 2/Fig 2E6.jpeg]

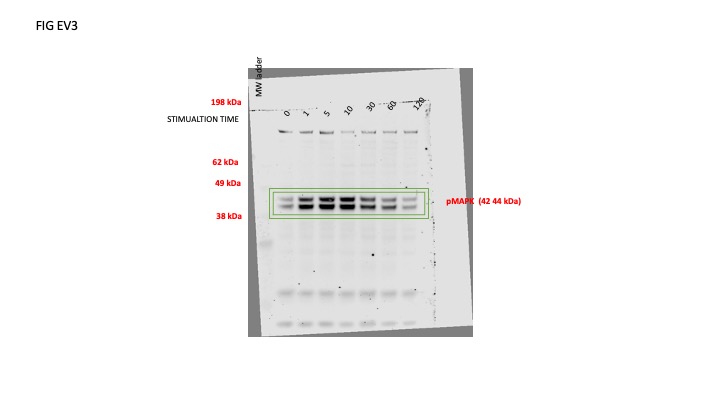

Supplement: Supplementary file 5 — Source Data Fig. 2 [file 44319_2023_11_MOESM5_ESM.zip › FIG 2/Fig 2E4.jpeg]

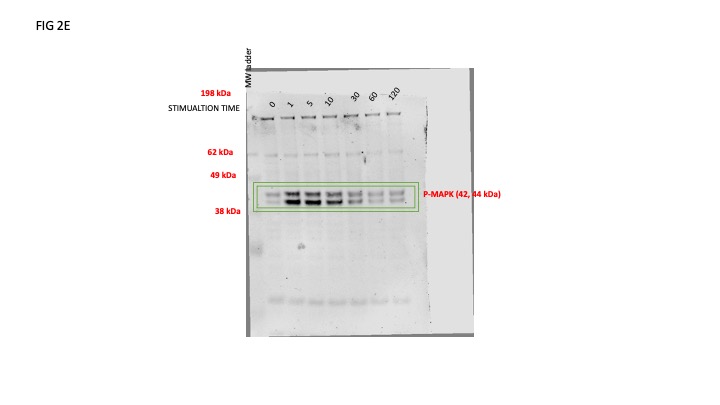

Supplement: Supplementary file 5 — Source Data Fig. 2 [file 44319_2023_11_MOESM5_ESM.zip › FIG 2/Fig 2E1 .jpeg]

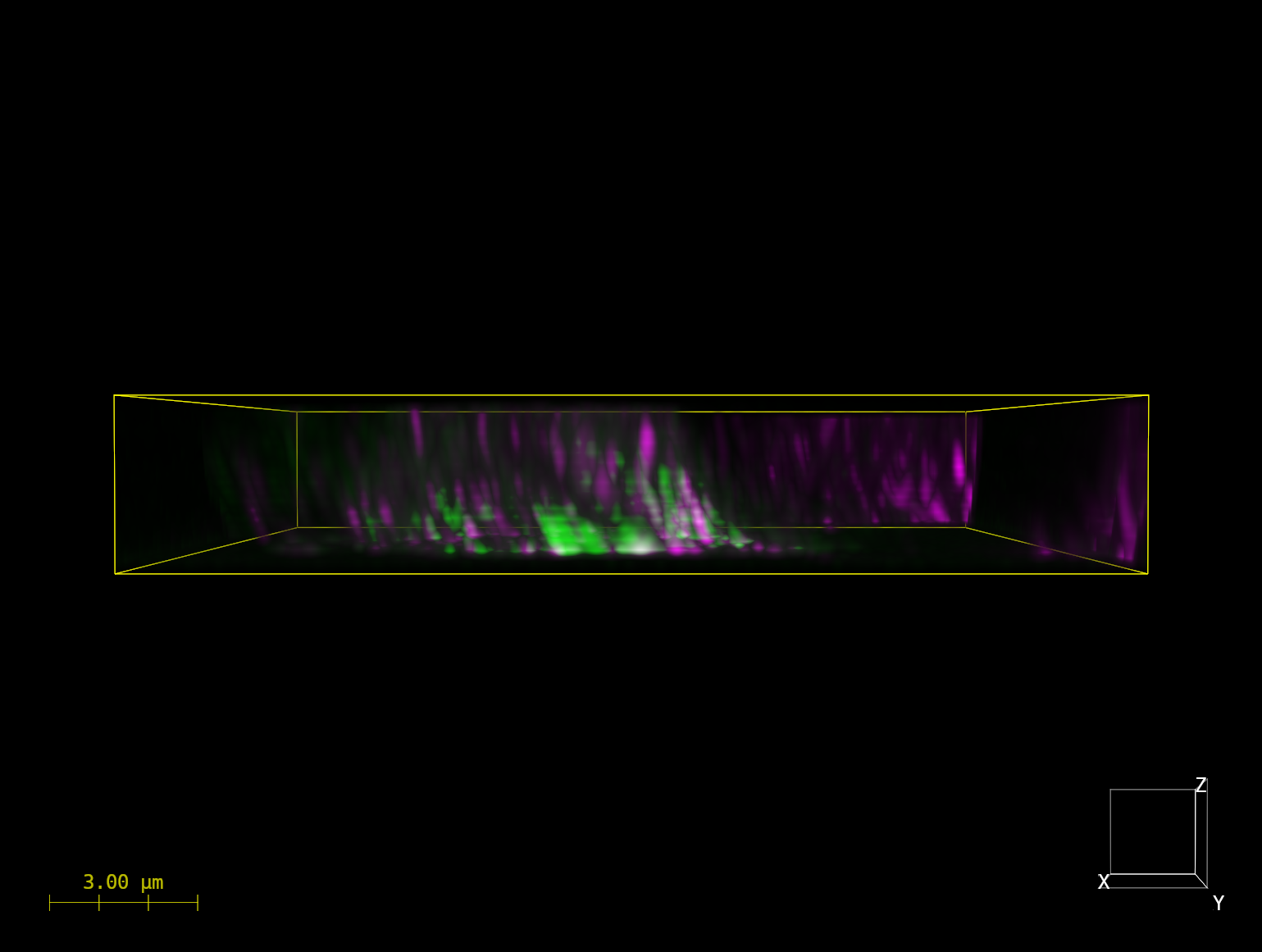

Supplement: Supplementary file 7 — Source Data Fig. 4 [file 44319_2023_11_MOESM7_ESM.zip › FIG 4/Figure 4F/2023-06-30-ESyt2KO_Jurkat-GFP-DAG-Cy3-CD4-Cy5-pLAT-Resting001_decon_MIP-3.tif]

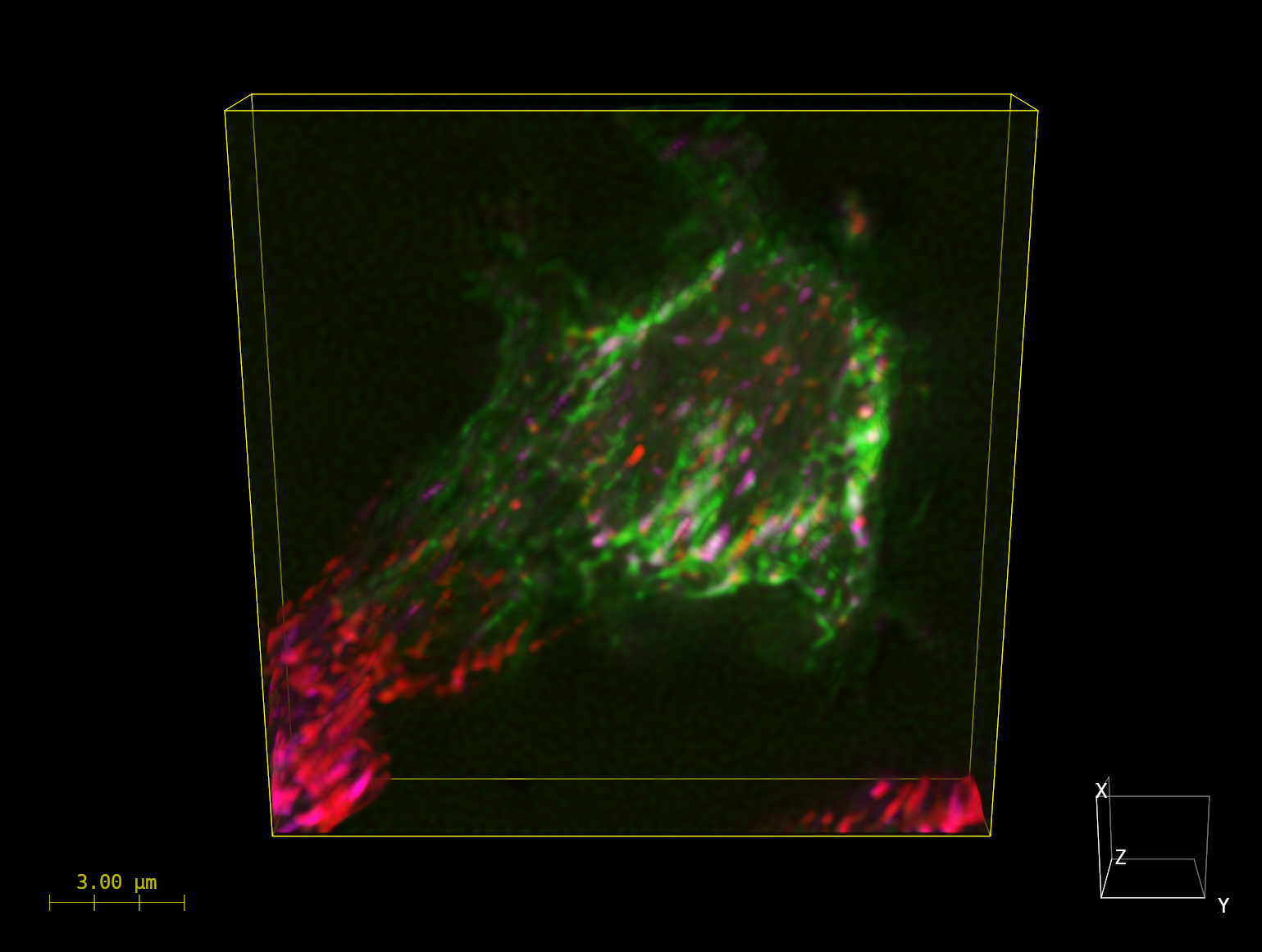

Supplement: Supplementary file 7 — Source Data Fig. 4 [file 44319_2023_11_MOESM7_ESM.zip › FIG 4/Figure 4F/2023-06-30-ESyt2KO_Jurkat-GFP-DAG-Cy3-CD4-Cy5-pLAT-Resting001_decon_MIP-2.tif]

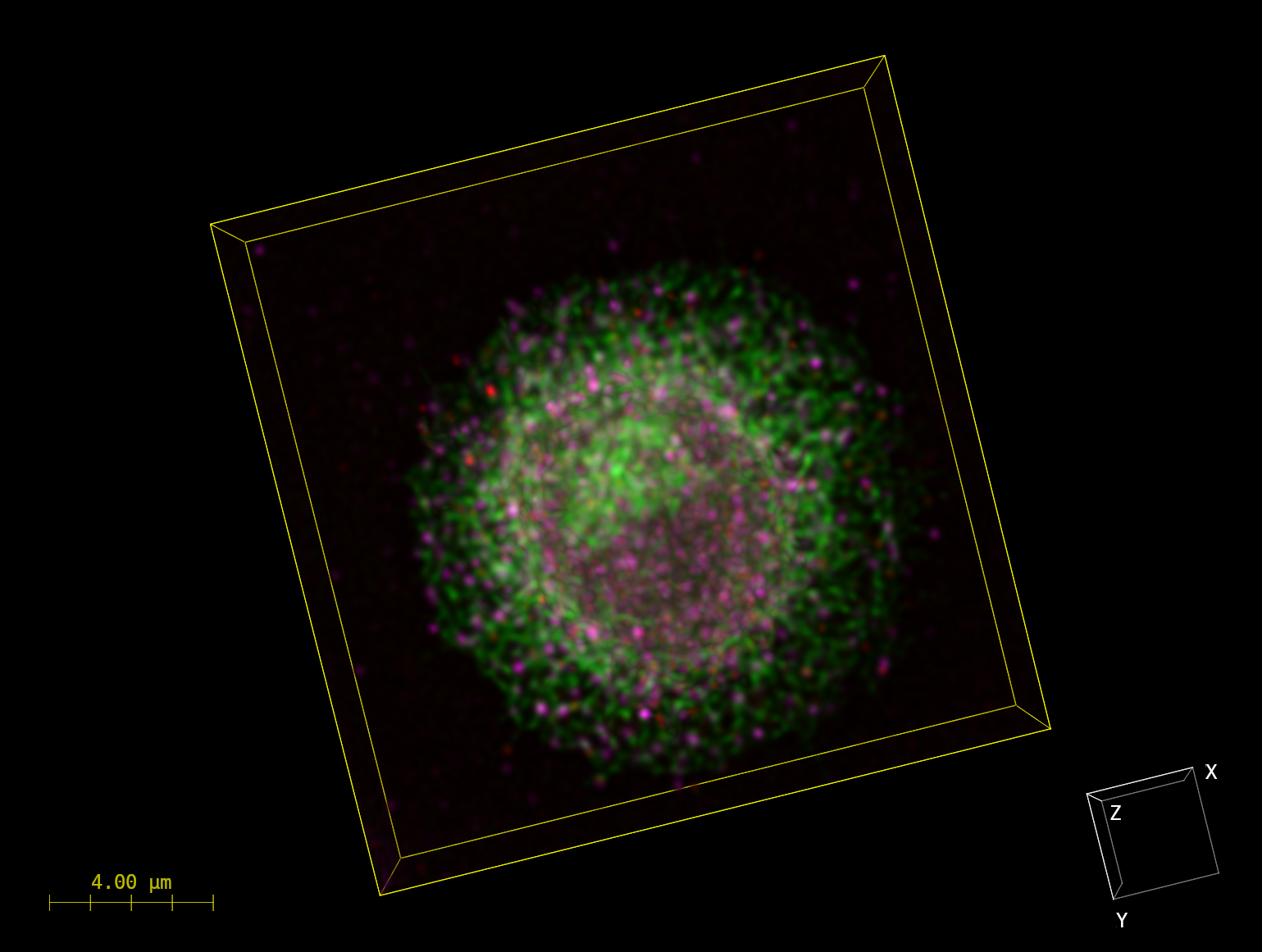

Supplement: Supplementary file 7 — Source Data Fig. 4 [file 44319_2023_11_MOESM7_ESM.zip › FIG 4/Figure 4G/2023-07-20-WT-Jurkat-GFP-DAG-Cy3-CD4-Cy5-pLAT-Resting008_decon_MIP-2.tif]

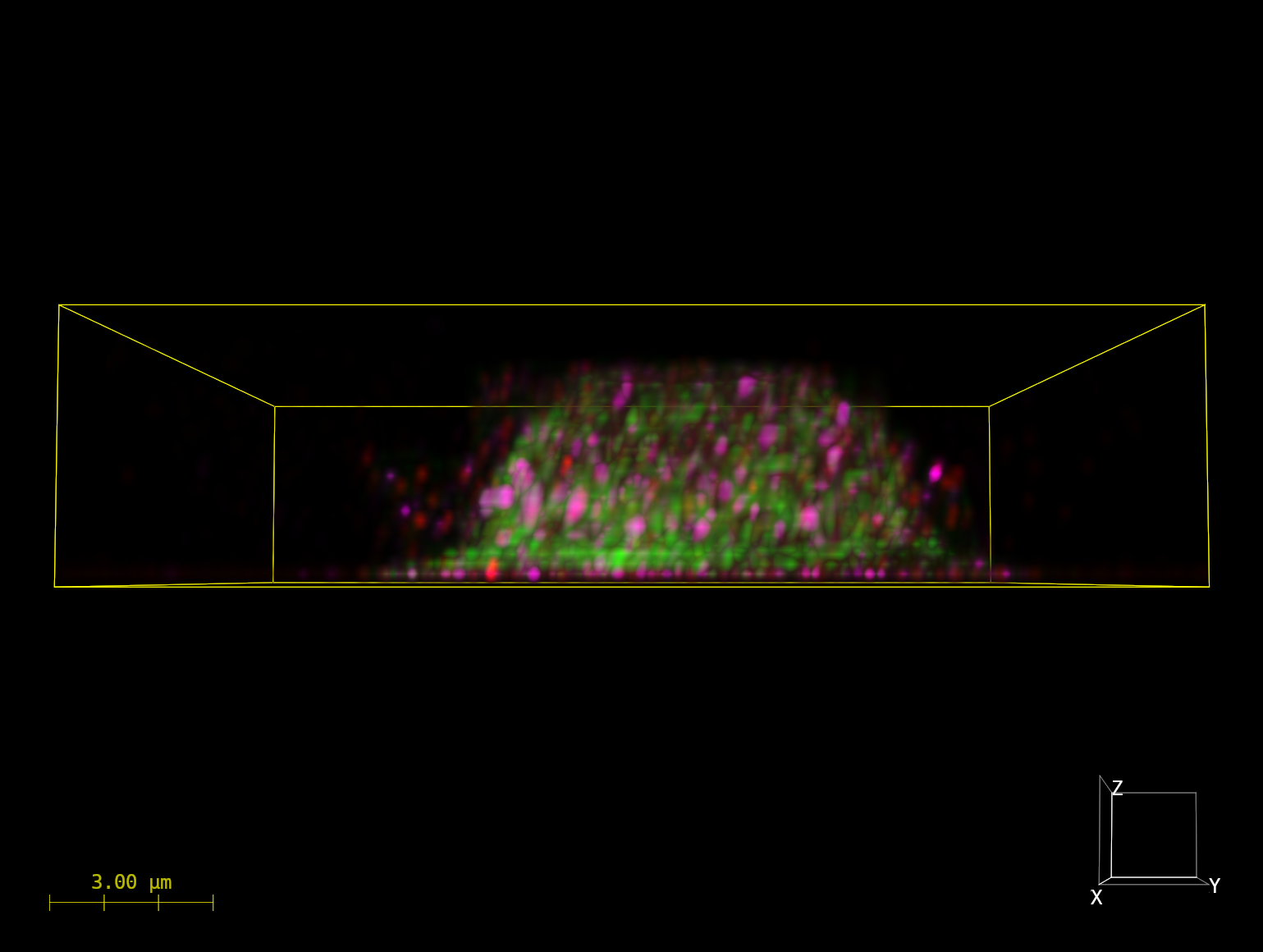

Supplement: Supplementary file 7 — Source Data Fig. 4 [file 44319_2023_11_MOESM7_ESM.zip › FIG 4/Figure 4G/2023-07-20-WT-Jurkat-GFP-DAG-Cy3-CD4-Cy5-pLAT-Resting008_decon_MIP-3.tif]

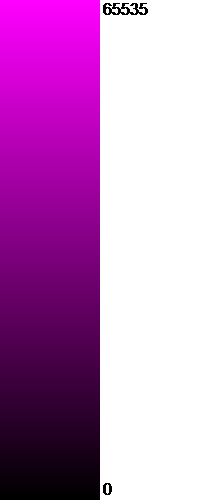

Supplement: Supplementary file 7 — Source Data Fig. 4 [file 44319_2023_11_MOESM7_ESM.zip › FIG 4/Figure 4A/MetaData/2023-08-21-NB-Jurkat-ESYT -cell markers 2_2023-08-23-WT-Jurkat-GFP-DAG-mCherry-ESYT1-Cy5-CD4-Resting003 ╧ä-STED1ch0LUT.png]

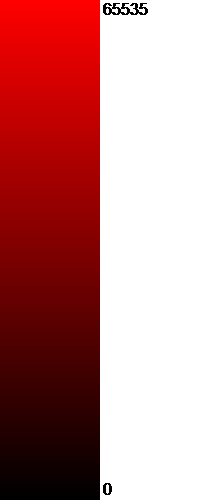

Supplement: Supplementary file 7 — Source Data Fig. 4 [file 44319_2023_11_MOESM7_ESM.zip › FIG 4/Figure 4A/MetaData/2023-08-21-NB-Jurkat-ESYT -cell markers 2_FLIM ╧ä-STED3ch1LUT.png]

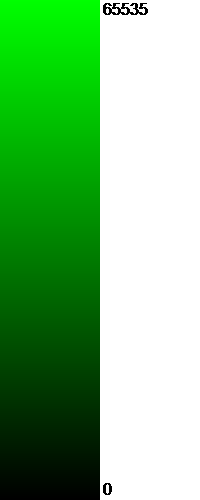

Supplement: Supplementary file 7 — Source Data Fig. 4 [file 44319_2023_11_MOESM7_ESM.zip › FIG 4/Figure 4A/MetaData/2023-08-21-NB-Jurkat-ESYT -cell markers 2_2023-08-23-WT-Jurkat-GFP-DAG-mCherry-ESYT1-Cy5-CD4-Resting003 ╧ä-STED1ch2LUT.png]

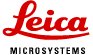

Supplement: Supplementary file 7 — Source Data Fig. 4 [file 44319_2023_11_MOESM7_ESM.zip › FIG 4/Figure 4A/MetaData/LeicaLogo.jpg]

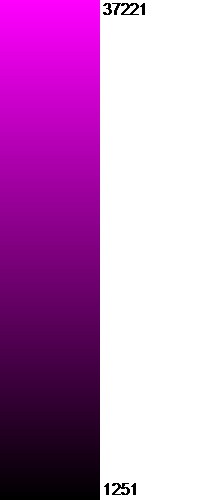

Supplement: Supplementary file 7 — Source Data Fig. 4 [file 44319_2023_11_MOESM7_ESM.zip › FIG 4/Figure 4A/MetaData/2023-08-21-NB-Jurkat-ESYT -cell markers 2_FLIM ╧ä-STED3ch0LUT.png]

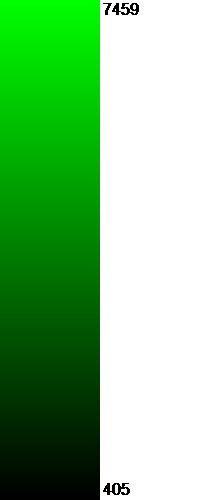

Supplement: Supplementary file 7 — Source Data Fig. 4 [file 44319_2023_11_MOESM7_ESM.zip › FIG 4/Figure 4A/MetaData/2023-08-21-NB-Jurkat-ESYT -cell markers 2_2023-08-22-WT-Jurkat-GFP-DAG-mCherry-ESyt2-Cy5-CD4-Resting006 ╧ä-STED1ch2LUT.png]

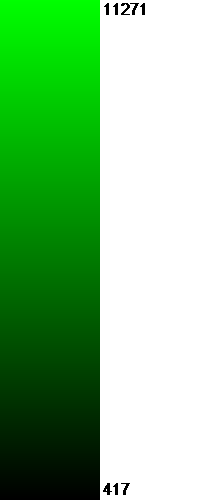

Supplement: Supplementary file 7 — Source Data Fig. 4 [file 44319_2023_11_MOESM7_ESM.zip › FIG 4/Figure 4A/MetaData/2023-08-21-NB-Jurkat-ESYT -cell markers 2_FLIM ╧ä-STED3ch2LUT.png]

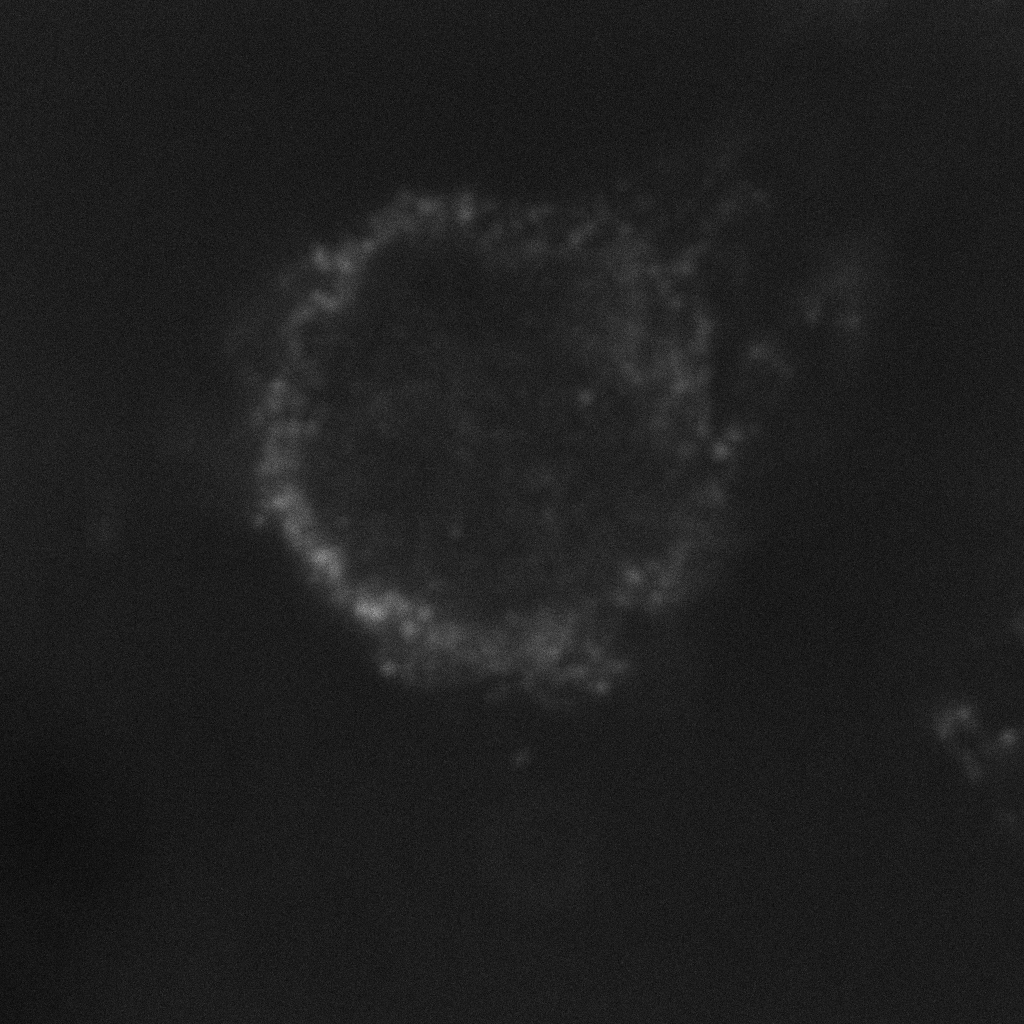

Supplement: Supplementary file 7 — Source Data Fig. 4 [file 44319_2023_11_MOESM7_ESM.zip › FIG 4/Figure 4B/ESYT1&2 DKO/Activated/2023-06-30 NB Jurkat cells ESyt manuscript revisions001_2023-07-05-ESyt1&@DKO-GFP-DAG-Cy3-CD4-Cy5-pLAT-Activated024_ch02.tif]

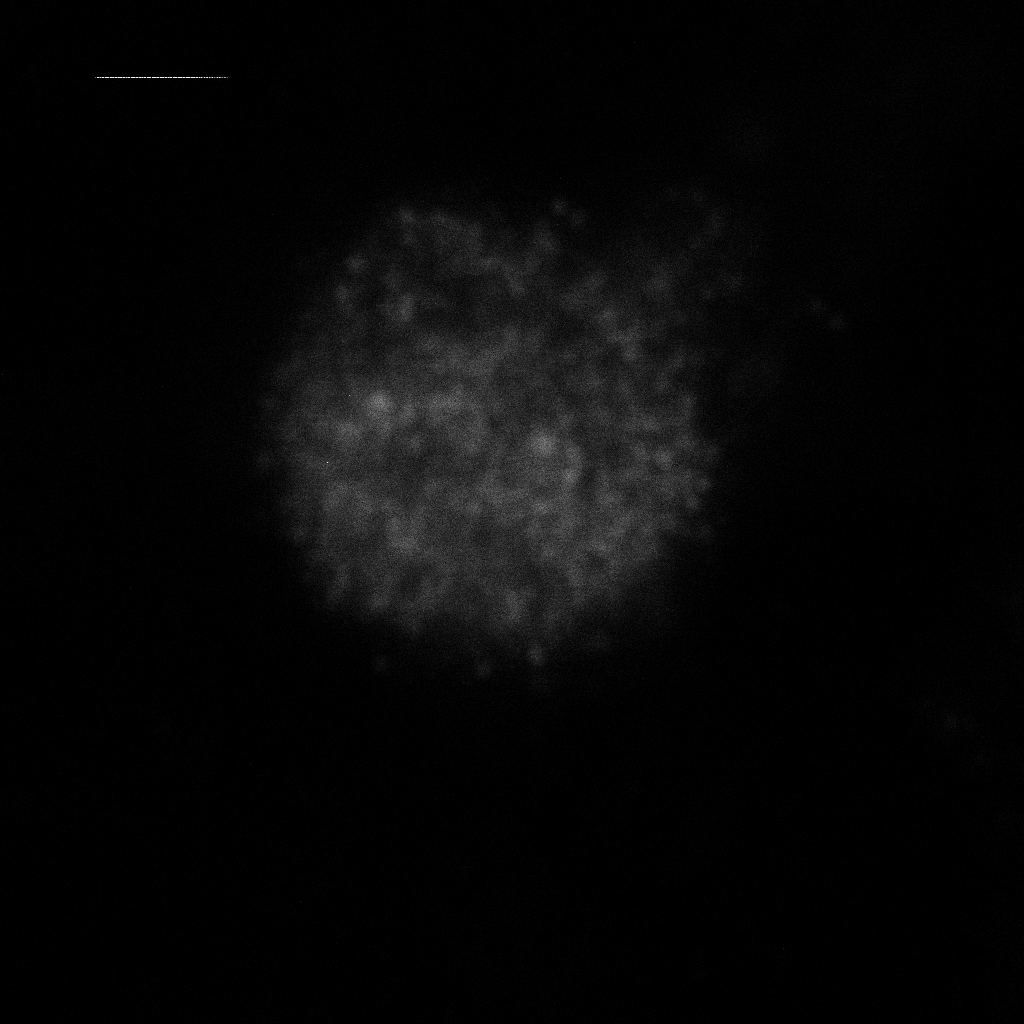

Supplement: Supplementary file 7 — Source Data Fig. 4 [file 44319_2023_11_MOESM7_ESM.zip › FIG 4/Figure 4B/ESYT1&2 DKO/Activated/2023-06-30 NB Jurkat cells ESyt manuscript revisions001_2023-07-05-ESyt1&@DKO-GFP-DAG-Cy3-CD4-Cy5-pLAT-Activated024_ch03.tif]

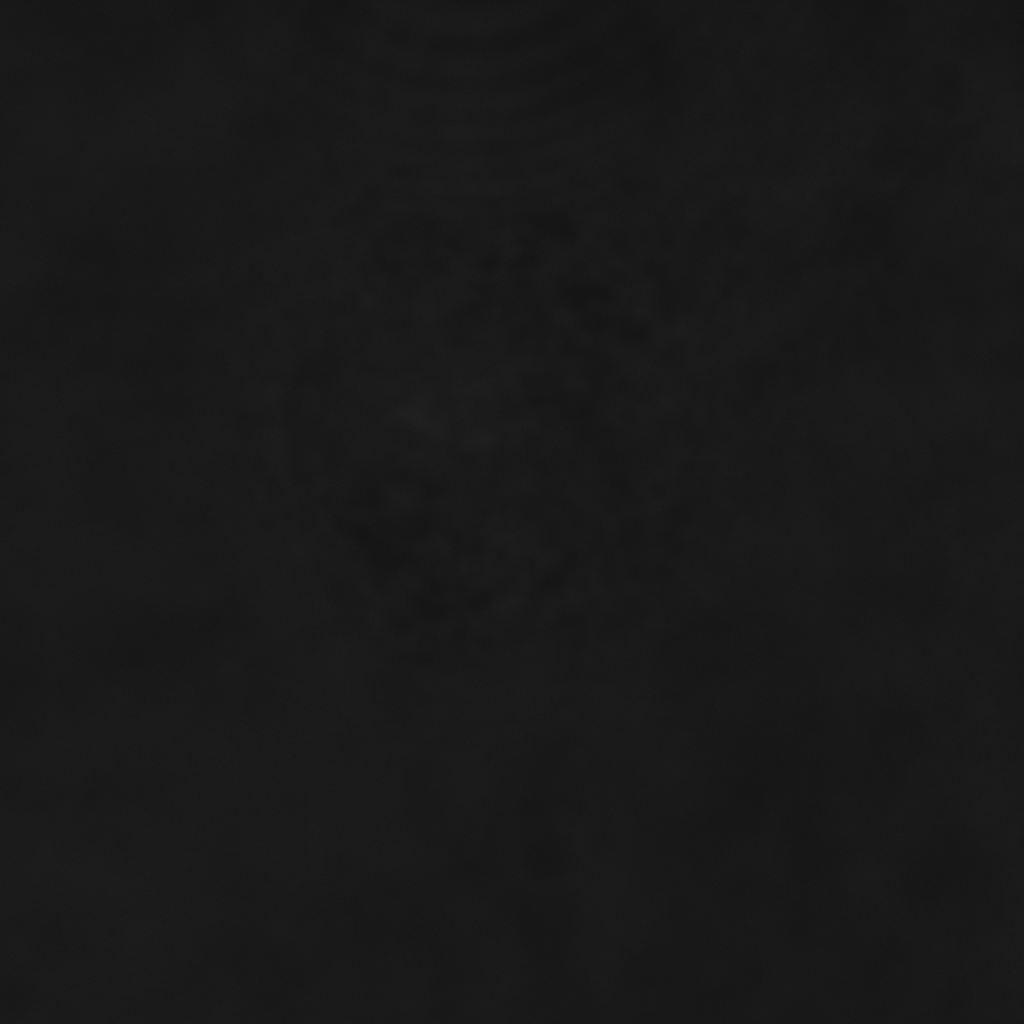

Supplement: Supplementary file 7 — Source Data Fig. 4 [file 44319_2023_11_MOESM7_ESM.zip › FIG 4/Figure 4B/ESYT1&2 DKO/Activated/2023-06-30 NB Jurkat cells ESyt manuscript revisions001_2023-07-05-ESyt1&@DKO-GFP-DAG-Cy3-CD4-Cy5-pLAT-Activated024_ch01.tif]

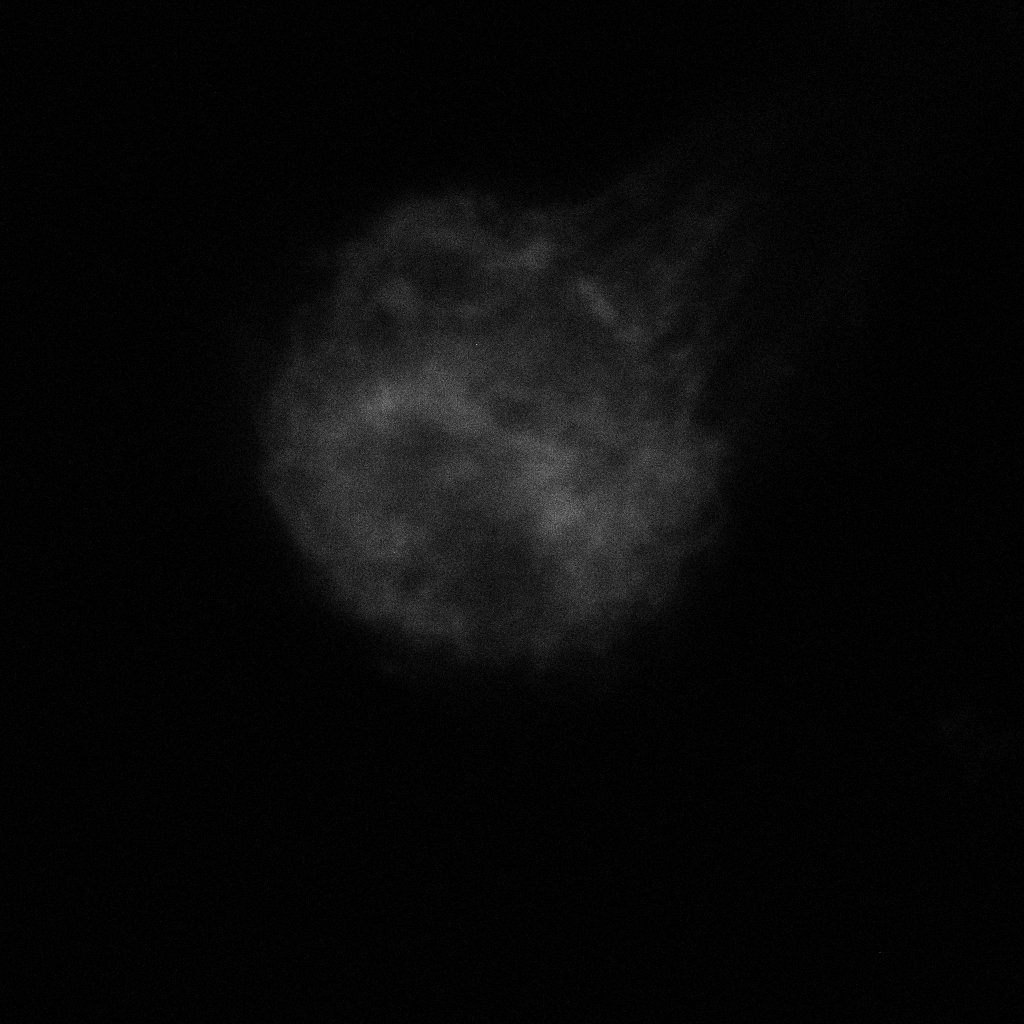

Supplement: Supplementary file 7 — Source Data Fig. 4 [file 44319_2023_11_MOESM7_ESM.zip › FIG 4/Figure 4B/ESYT1&2 DKO/Activated/2023-06-30 NB Jurkat cells ESyt manuscript revisions001_2023-07-05-ESyt1&@DKO-GFP-DAG-Cy3-CD4-Cy5-pLAT-Activated024_ch00.tif]

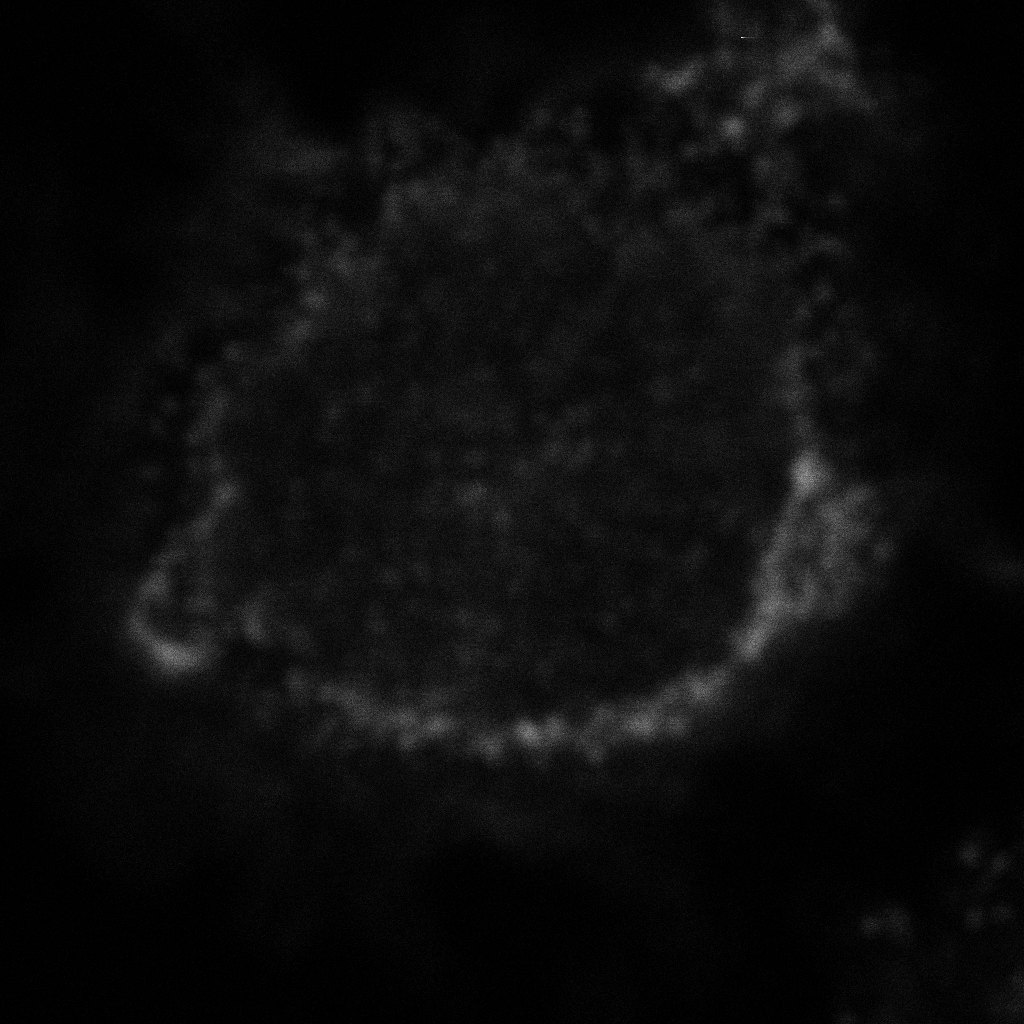

Supplement: Supplementary file 7 — Source Data Fig. 4 [file 44319_2023_11_MOESM7_ESM.zip › FIG 4/Figure 4B/ESYT1&2 DKO/Resting/2023-06-30 NB Jurkat cells ESyt manuscript revisions001_2023-07-05-ESyt1&@DKO-GFP-DAG-Cy3-CD4-Cy5-pLAT-Resting004_ch02.tif]

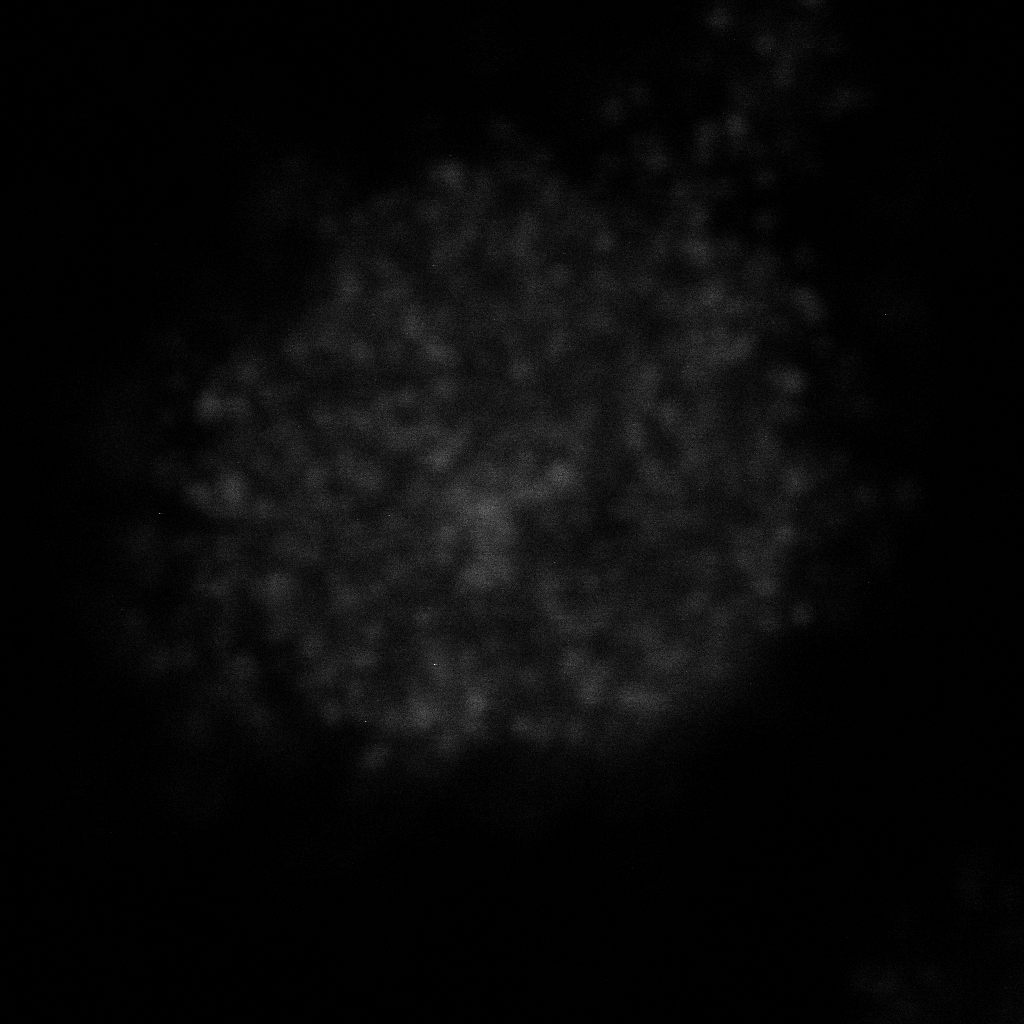

Supplement: Supplementary file 7 — Source Data Fig. 4 [file 44319_2023_11_MOESM7_ESM.zip › FIG 4/Figure 4B/ESYT1&2 DKO/Resting/2023-06-30 NB Jurkat cells ESyt manuscript revisions001_2023-07-05-ESyt1&@DKO-GFP-DAG-Cy3-CD4-Cy5-pLAT-Resting004_ch03.tif]

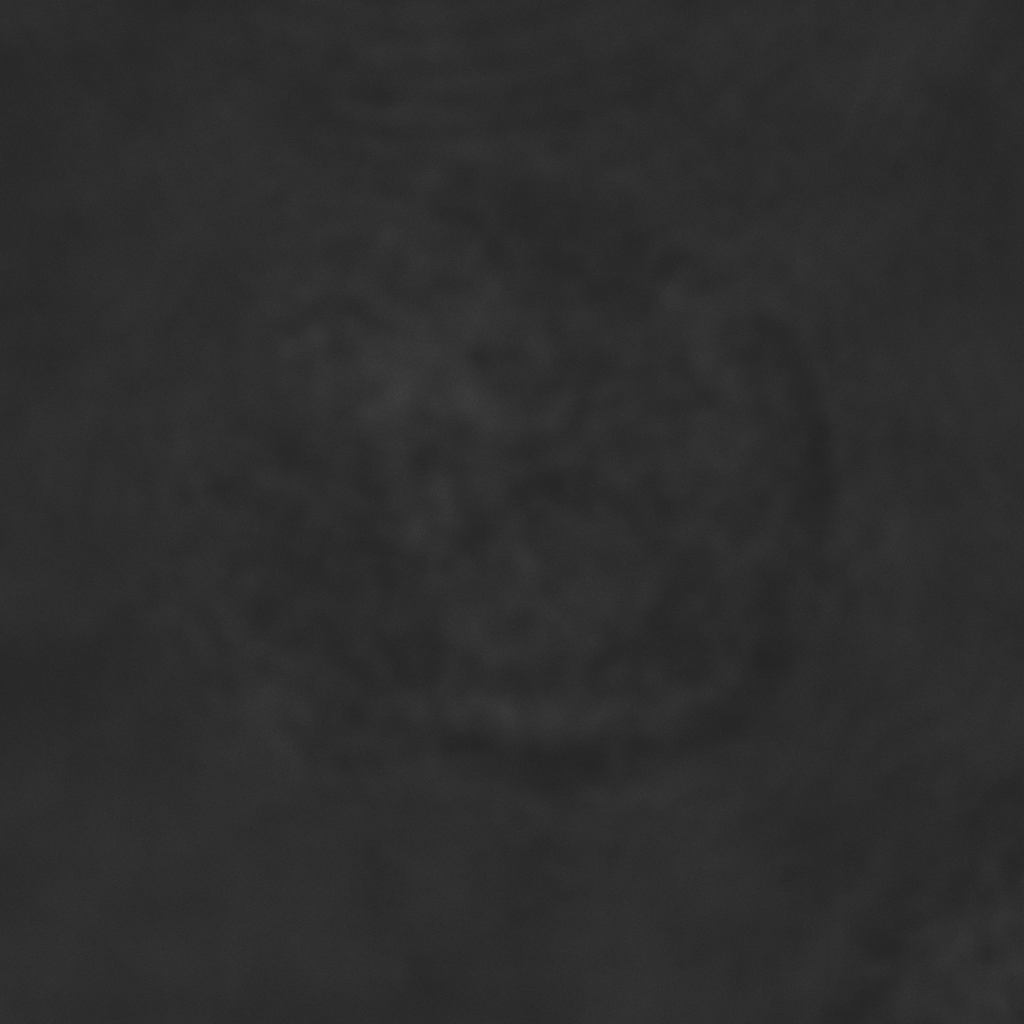

Supplement: Supplementary file 7 — Source Data Fig. 4 [file 44319_2023_11_MOESM7_ESM.zip › FIG 4/Figure 4B/ESYT1&2 DKO/Resting/2023-06-30 NB Jurkat cells ESyt manuscript revisions001_2023-07-05-ESyt1&@DKO-GFP-DAG-Cy3-CD4-Cy5-pLAT-Resting004_ch01.tif]

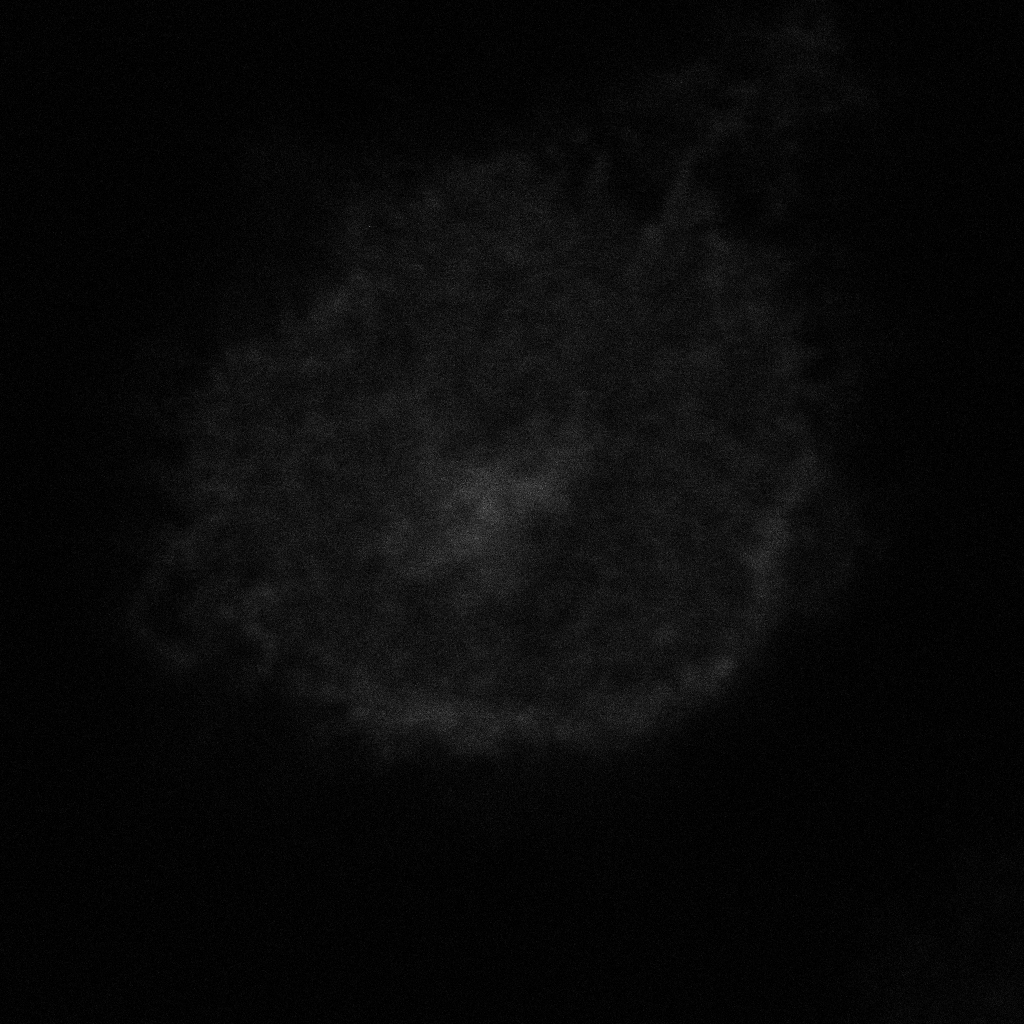

Supplement: Supplementary file 7 — Source Data Fig. 4 [file 44319_2023_11_MOESM7_ESM.zip › FIG 4/Figure 4B/ESYT1&2 DKO/Resting/2023-06-30 NB Jurkat cells ESyt manuscript revisions001_2023-07-05-ESyt1&@DKO-GFP-DAG-Cy3-CD4-Cy5-pLAT-Resting004_ch00.tif]

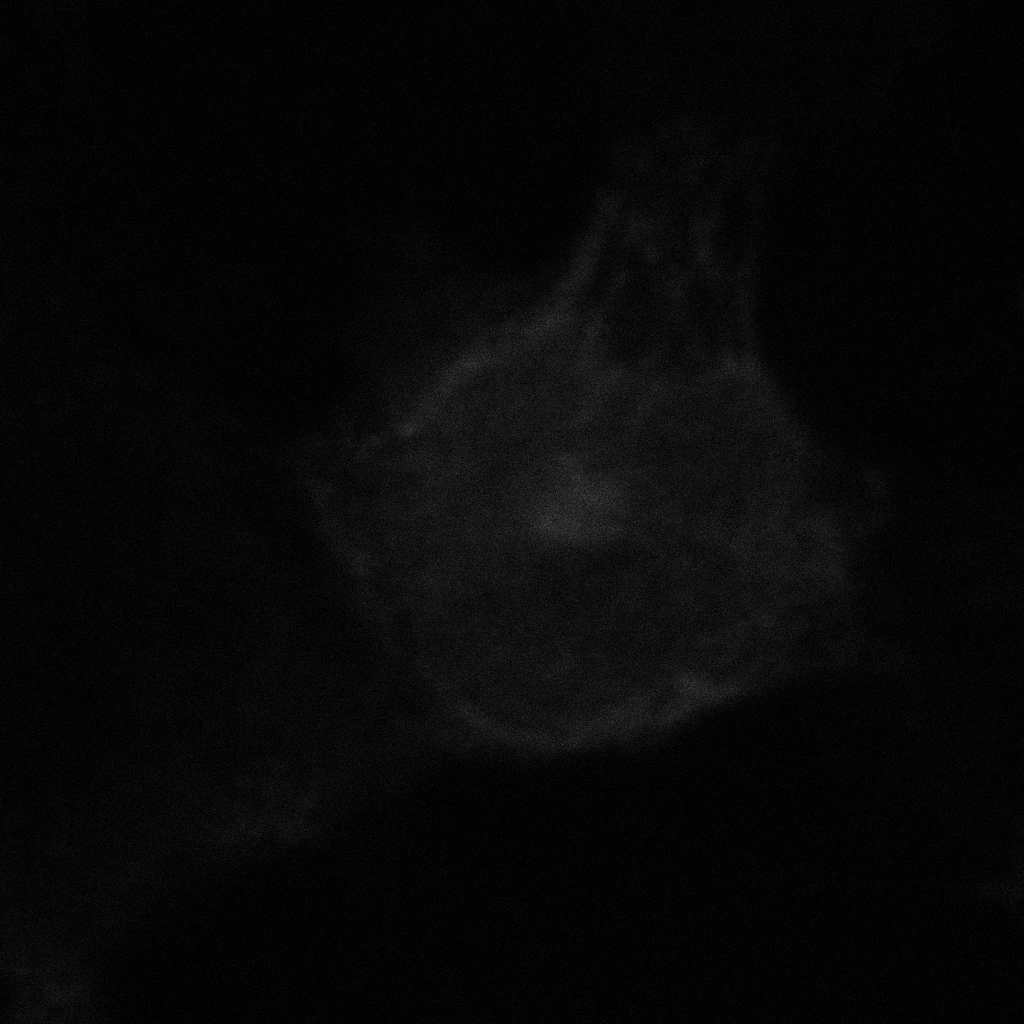

Supplement: Supplementary file 7 — Source Data Fig. 4 [file 44319_2023_11_MOESM7_ESM.zip › FIG 4/Figure 4B/ESYT2 KO/Activated/2023-06-30 NB Jurkat cells ESyt manuscript revisions001_2023-07-03-ESyt2KO Jurkat-GFP-DAG-Cy3-CD4-Cy5-pLAT-Activated009_ch00.tif]

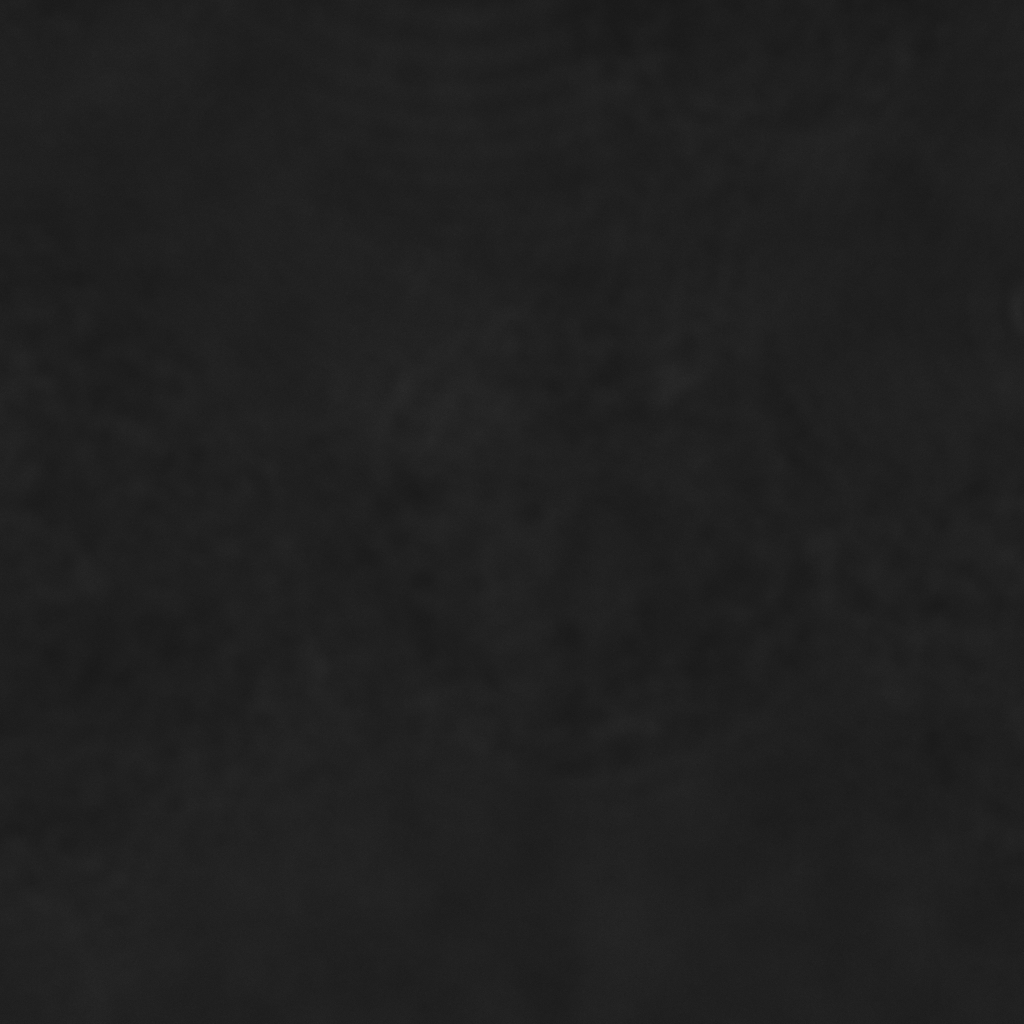

Supplement: Supplementary file 7 — Source Data Fig. 4 [file 44319_2023_11_MOESM7_ESM.zip › FIG 4/Figure 4B/ESYT2 KO/Activated/2023-06-30 NB Jurkat cells ESyt manuscript revisions001_2023-07-03-ESyt2KO Jurkat-GFP-DAG-Cy3-CD4-Cy5-pLAT-Activated009_ch01.tif]

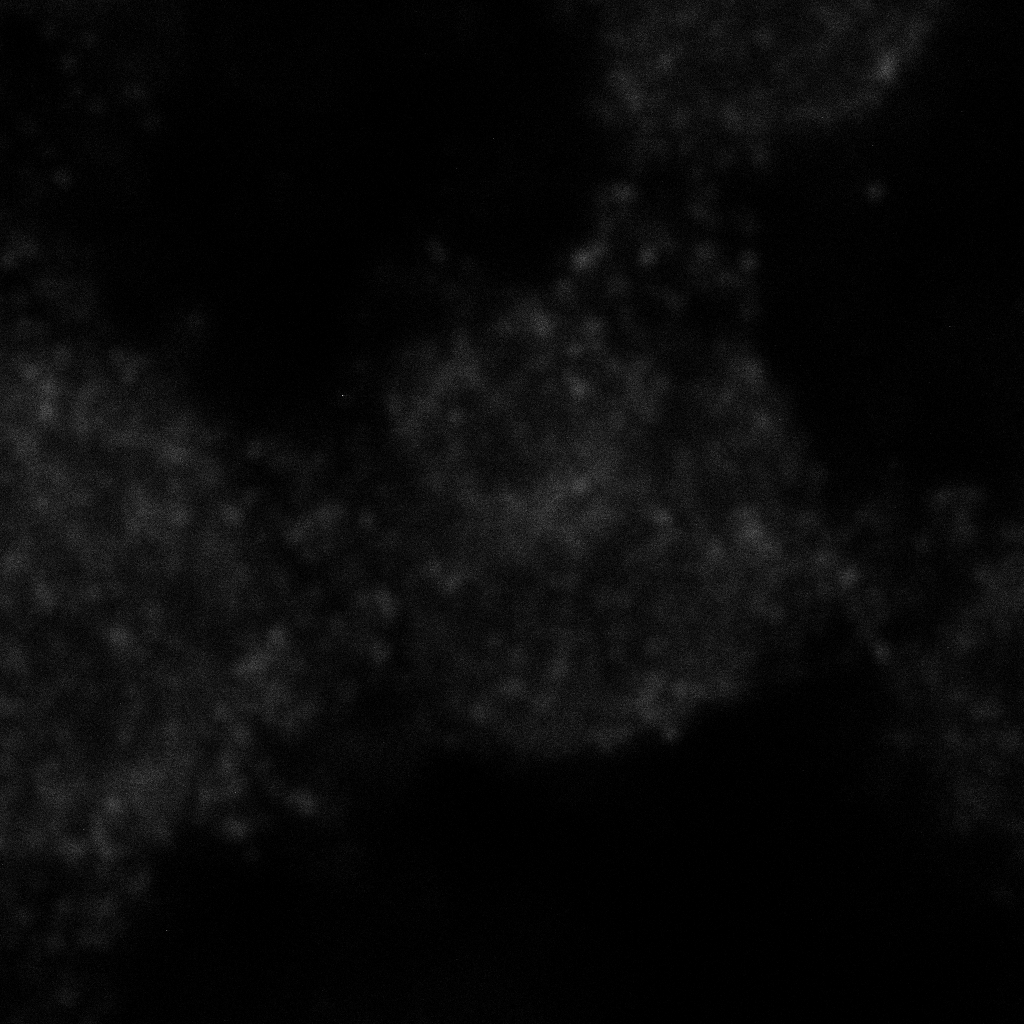

Supplement: Supplementary file 7 — Source Data Fig. 4 [file 44319_2023_11_MOESM7_ESM.zip › FIG 4/Figure 4B/ESYT2 KO/Activated/2023-06-30 NB Jurkat cells ESyt manuscript revisions001_2023-07-03-ESyt2KO Jurkat-GFP-DAG-Cy3-CD4-Cy5-pLAT-Activated009_ch03.tif]

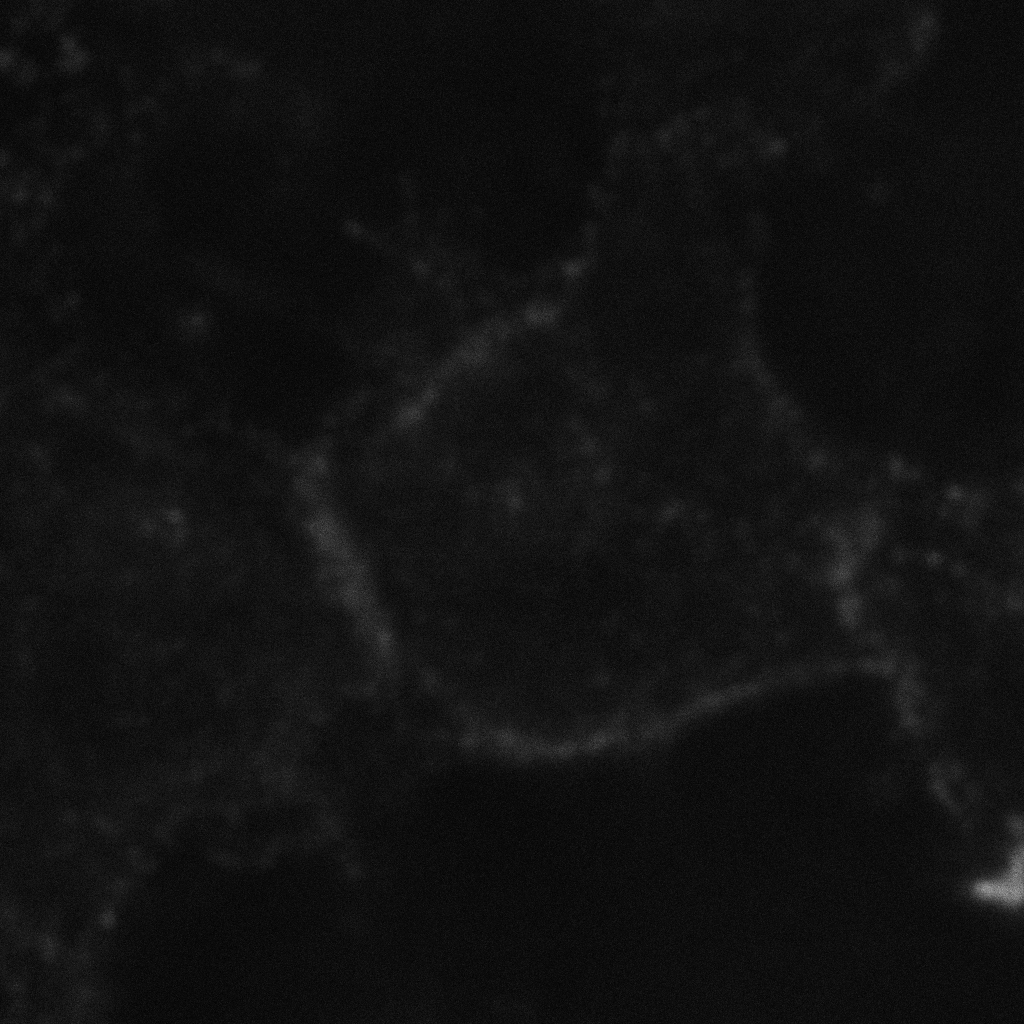

Supplement: Supplementary file 7 — Source Data Fig. 4 [file 44319_2023_11_MOESM7_ESM.zip › FIG 4/Figure 4B/ESYT2 KO/Activated/2023-06-30 NB Jurkat cells ESyt manuscript revisions001_2023-07-03-ESyt2KO Jurkat-GFP-DAG-Cy3-CD4-Cy5-pLAT-Activated009_ch02.tif]

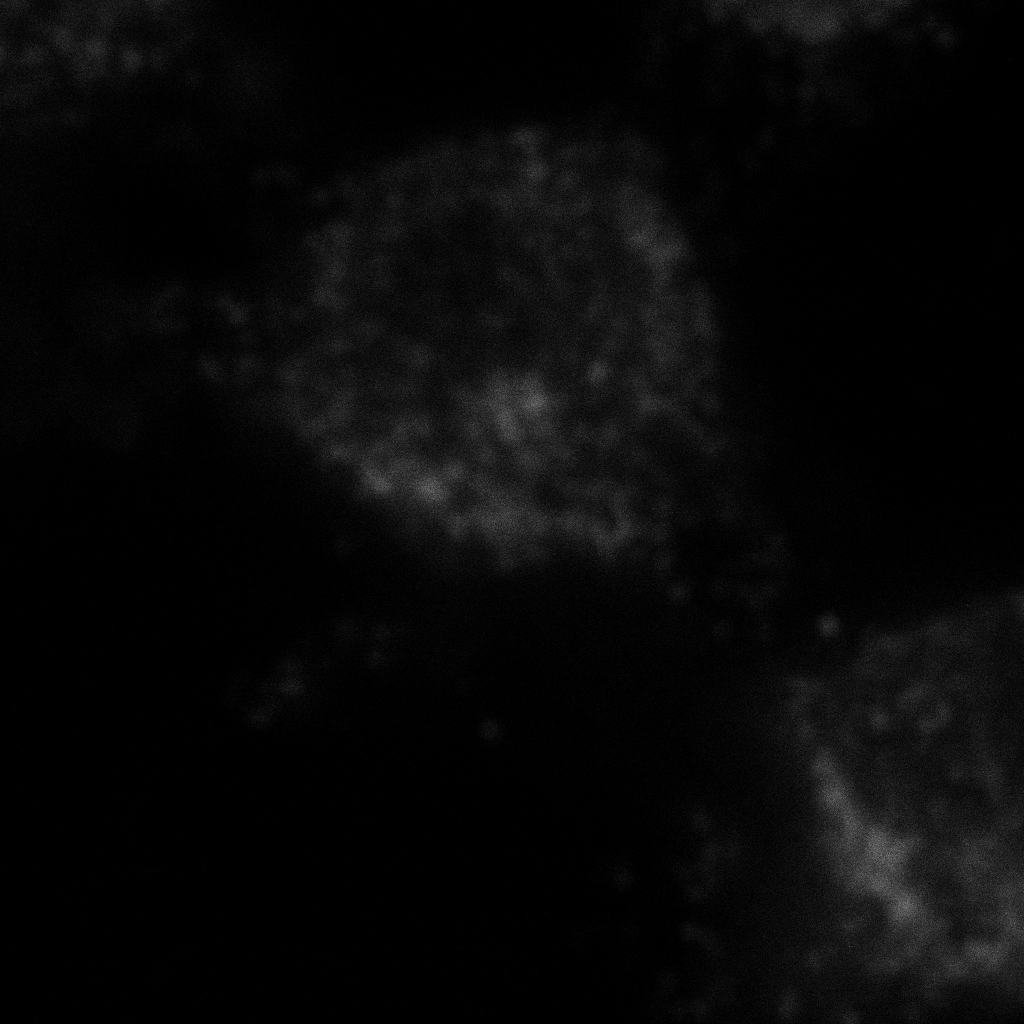

Supplement: Supplementary file 7 — Source Data Fig. 4 [file 44319_2023_11_MOESM7_ESM.zip › FIG 4/Figure 4B/ESYT2 KO/Resting/2023-06-30 NB Jurkat cells ESyt manuscript revisions001_2023-06-30-ESyt2KO Jurkat-GFP-DAG-Cy3-CD4-Cy5-pLAT-Resting002_ch03.tif]

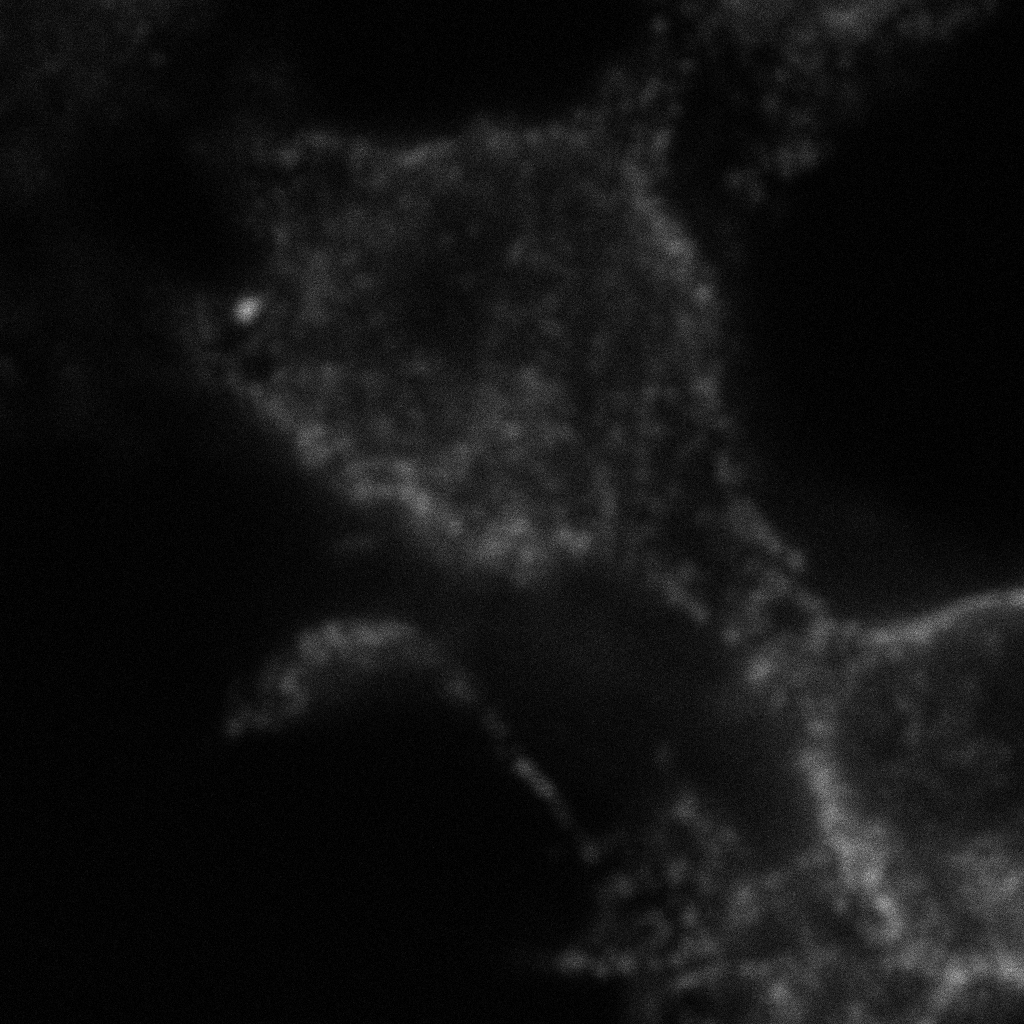

Supplement: Supplementary file 7 — Source Data Fig. 4 [file 44319_2023_11_MOESM7_ESM.zip › FIG 4/Figure 4B/ESYT2 KO/Resting/2023-06-30 NB Jurkat cells ESyt manuscript revisions001_2023-06-30-ESyt2KO Jurkat-GFP-DAG-Cy3-CD4-Cy5-pLAT-Resting002_ch02.tif]

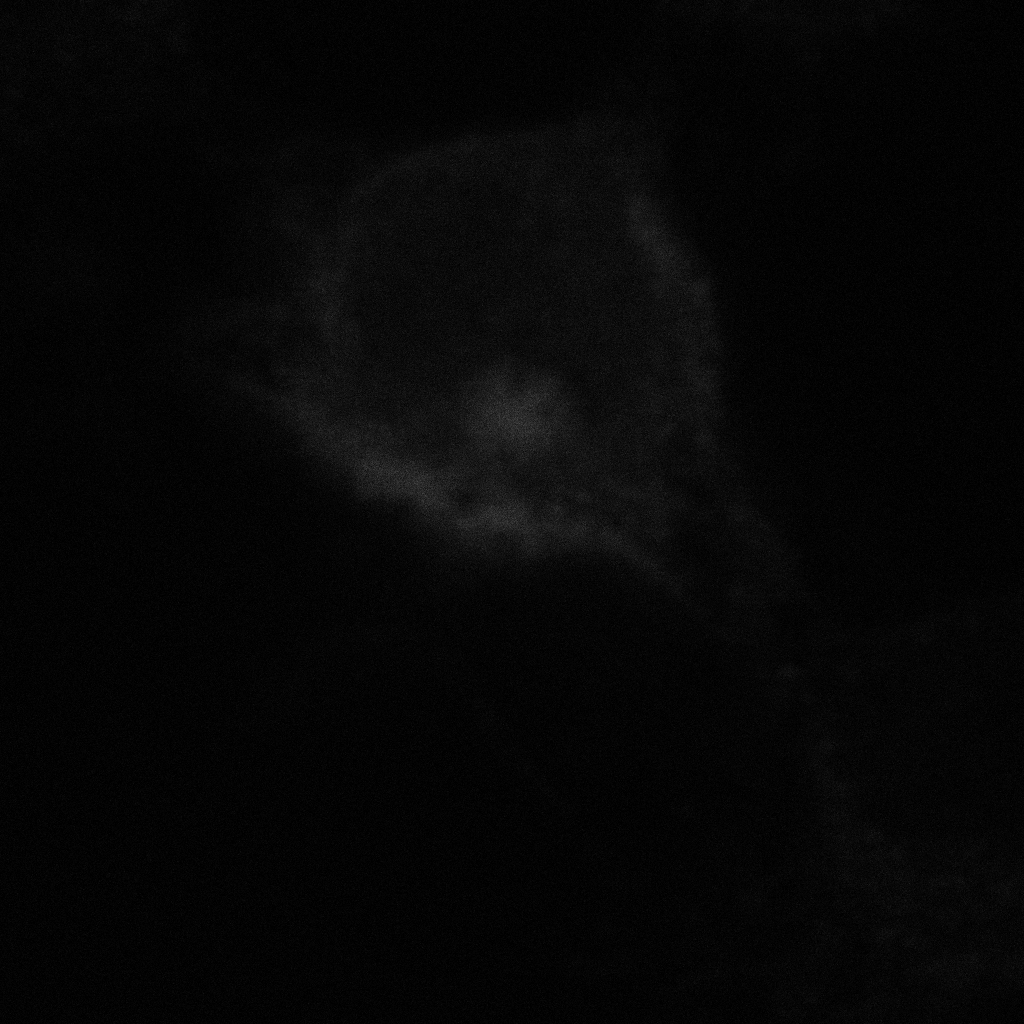

Supplement: Supplementary file 7 — Source Data Fig. 4 [file 44319_2023_11_MOESM7_ESM.zip › FIG 4/Figure 4B/ESYT2 KO/Resting/2023-06-30 NB Jurkat cells ESyt manuscript revisions001_2023-06-30-ESyt2KO Jurkat-GFP-DAG-Cy3-CD4-Cy5-pLAT-Resting002_ch00.tif]

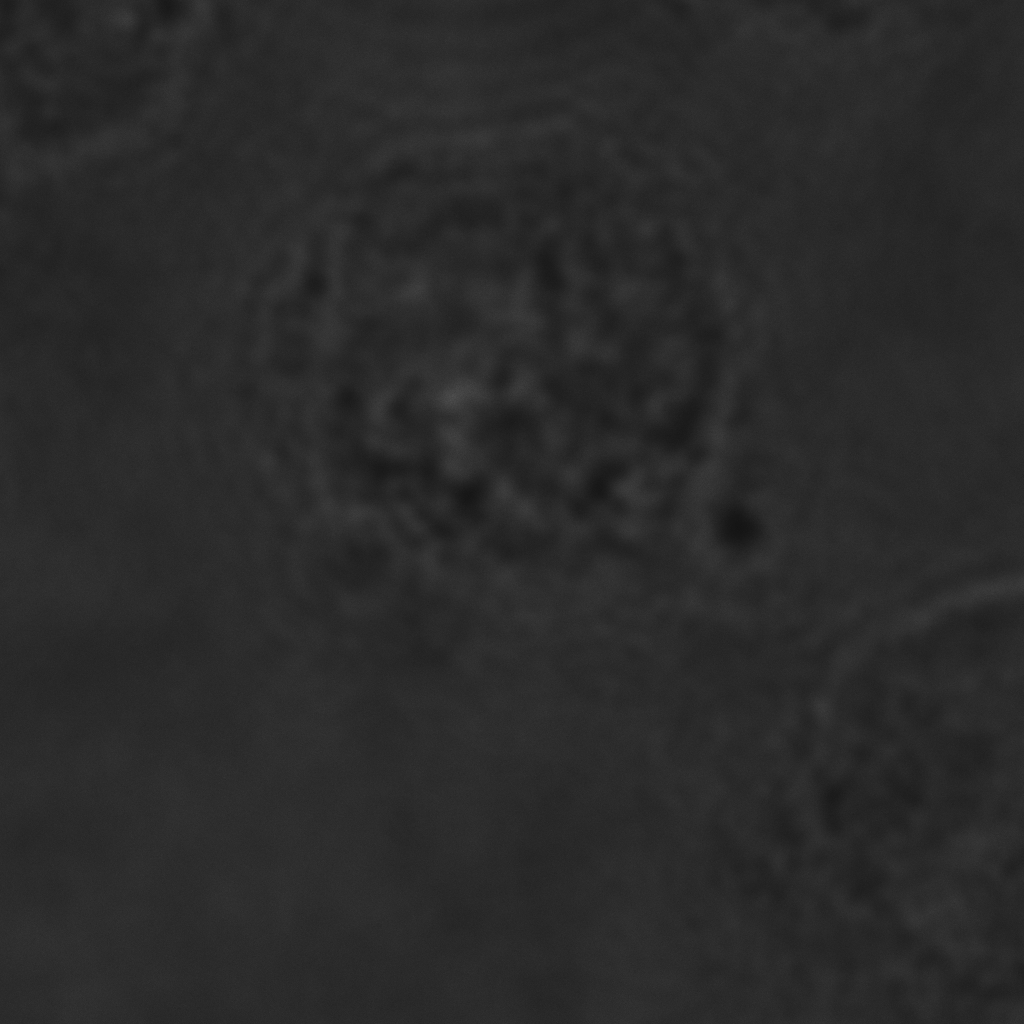

Supplement: Supplementary file 7 — Source Data Fig. 4 [file 44319_2023_11_MOESM7_ESM.zip › FIG 4/Figure 4B/ESYT2 KO/Resting/2023-06-30 NB Jurkat cells ESyt manuscript revisions001_2023-06-30-ESyt2KO Jurkat-GFP-DAG-Cy3-CD4-Cy5-pLAT-Resting002_ch01.tif]

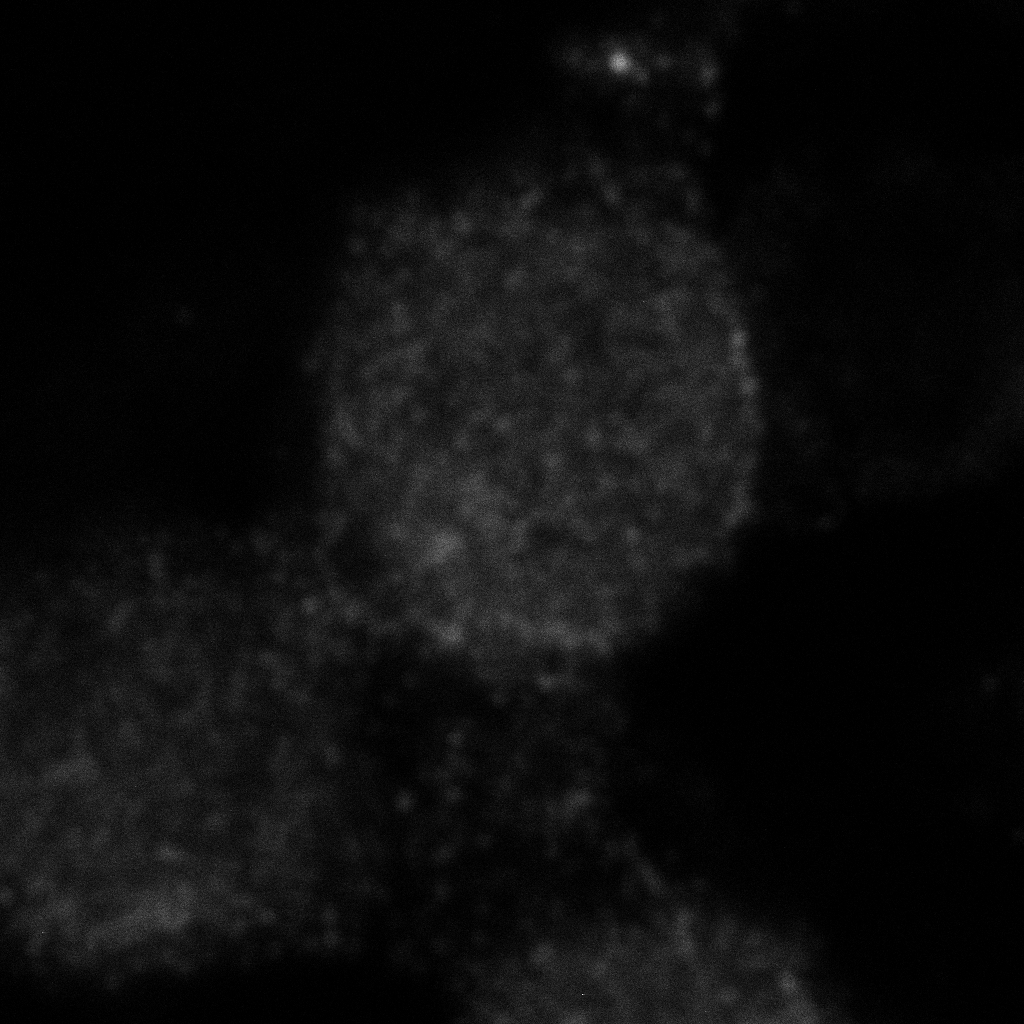

Supplement: Supplementary file 7 — Source Data Fig. 4 [file 44319_2023_11_MOESM7_ESM.zip › FIG 4/Figure 4B/ESYT1 KO/Activated/2023-06-30 NB Jurkat cells ESyt manuscript revisions001_2023-06-30-ESyt1KO Jurkat-GFP-DAG-Cy3-CD4-Cy5-pLAT-Activated012_ch03.tif]

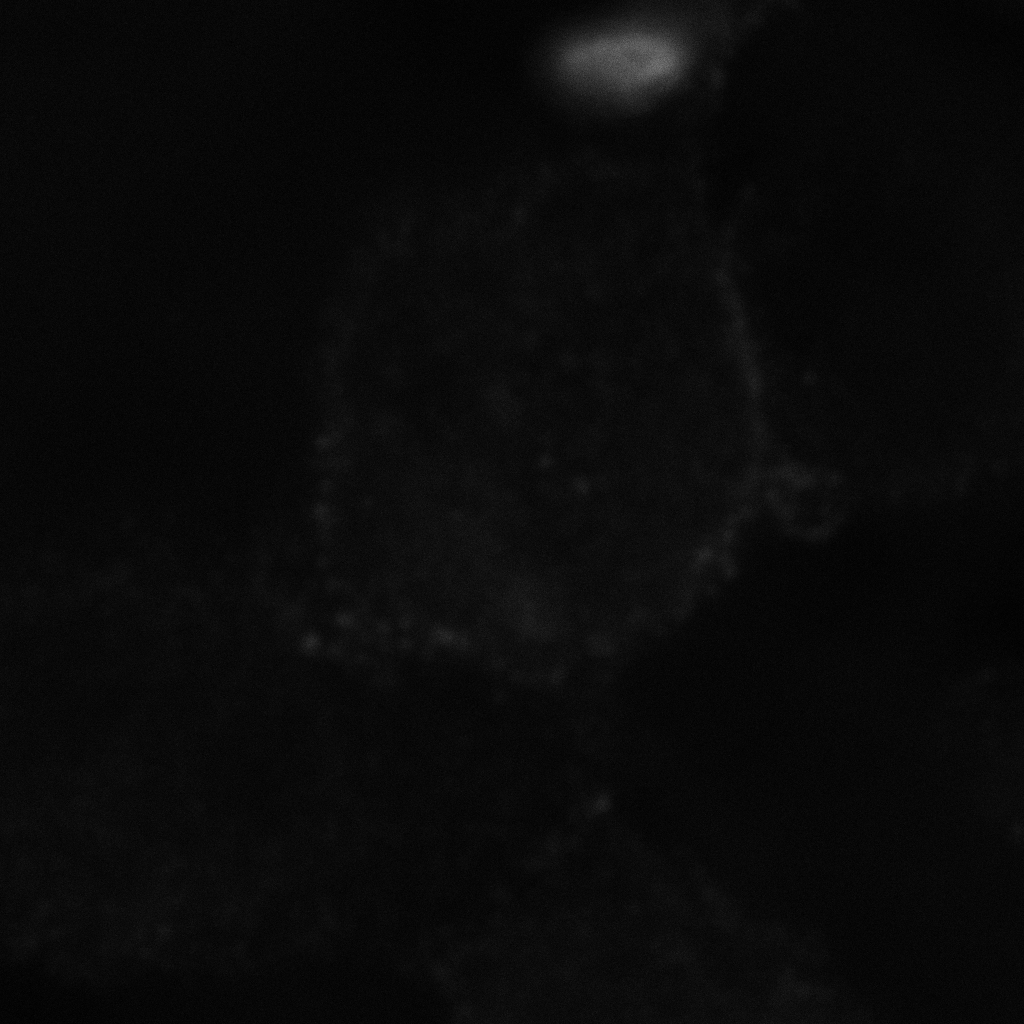

Supplement: Supplementary file 7 — Source Data Fig. 4 [file 44319_2023_11_MOESM7_ESM.zip › FIG 4/Figure 4B/ESYT1 KO/Activated/2023-06-30 NB Jurkat cells ESyt manuscript revisions001_2023-06-30-ESyt1KO Jurkat-GFP-DAG-Cy3-CD4-Cy5-pLAT-Activated012_ch02.tif]

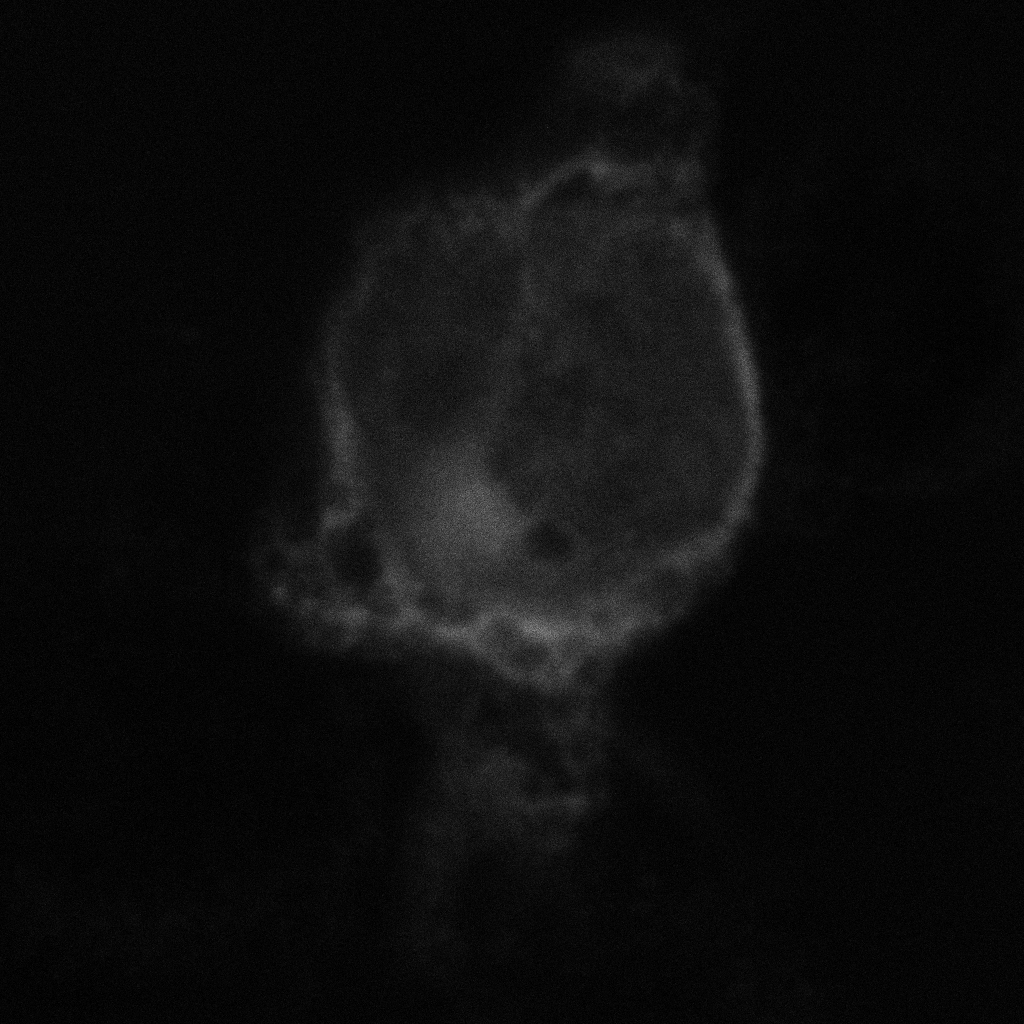

Supplement: Supplementary file 7 — Source Data Fig. 4 [file 44319_2023_11_MOESM7_ESM.zip › FIG 4/Figure 4B/ESYT1 KO/Activated/2023-06-30 NB Jurkat cells ESyt manuscript revisions001_2023-06-30-ESyt1KO Jurkat-GFP-DAG-Cy3-CD4-Cy5-pLAT-Activated012_ch00.tif]

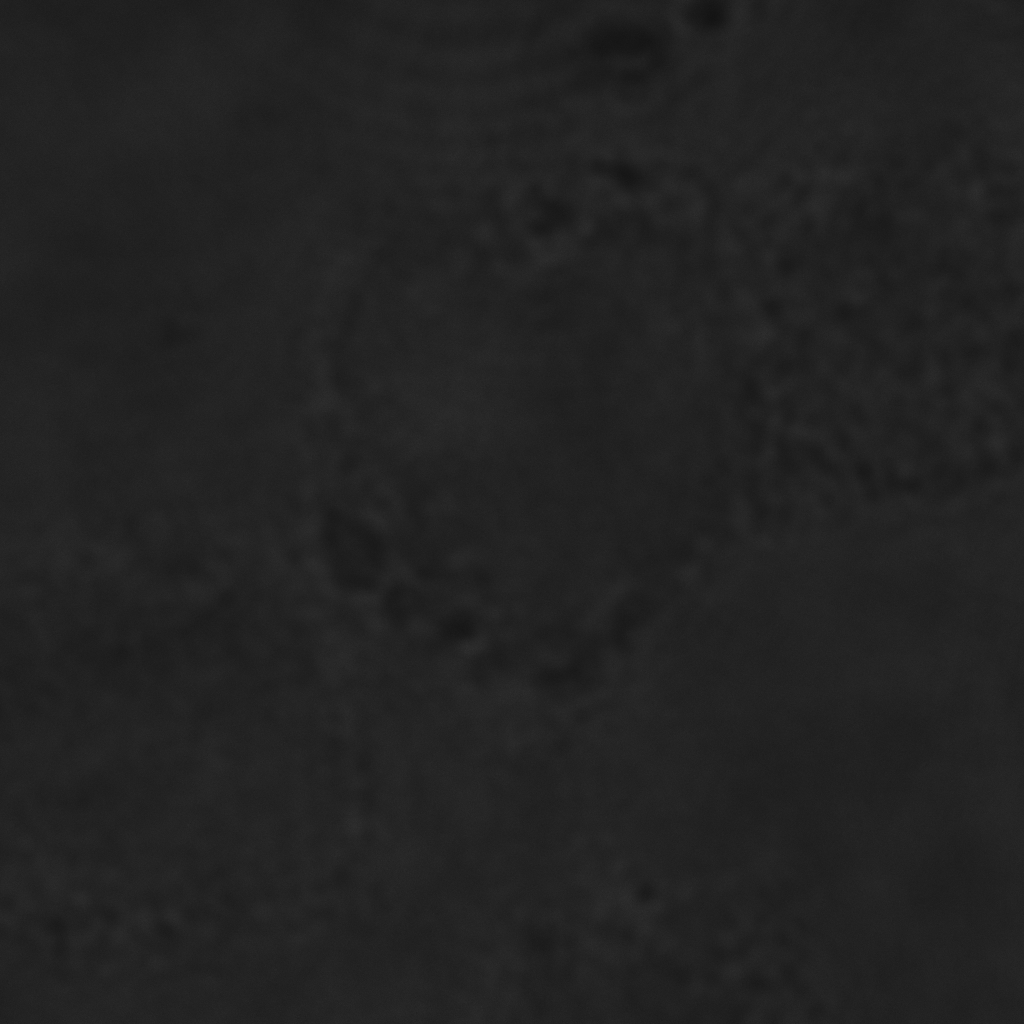

Supplement: Supplementary file 7 — Source Data Fig. 4 [file 44319_2023_11_MOESM7_ESM.zip › FIG 4/Figure 4B/ESYT1 KO/Activated/2023-06-30 NB Jurkat cells ESyt manuscript revisions001_2023-06-30-ESyt1KO Jurkat-GFP-DAG-Cy3-CD4-Cy5-pLAT-Activated012_ch01.tif]

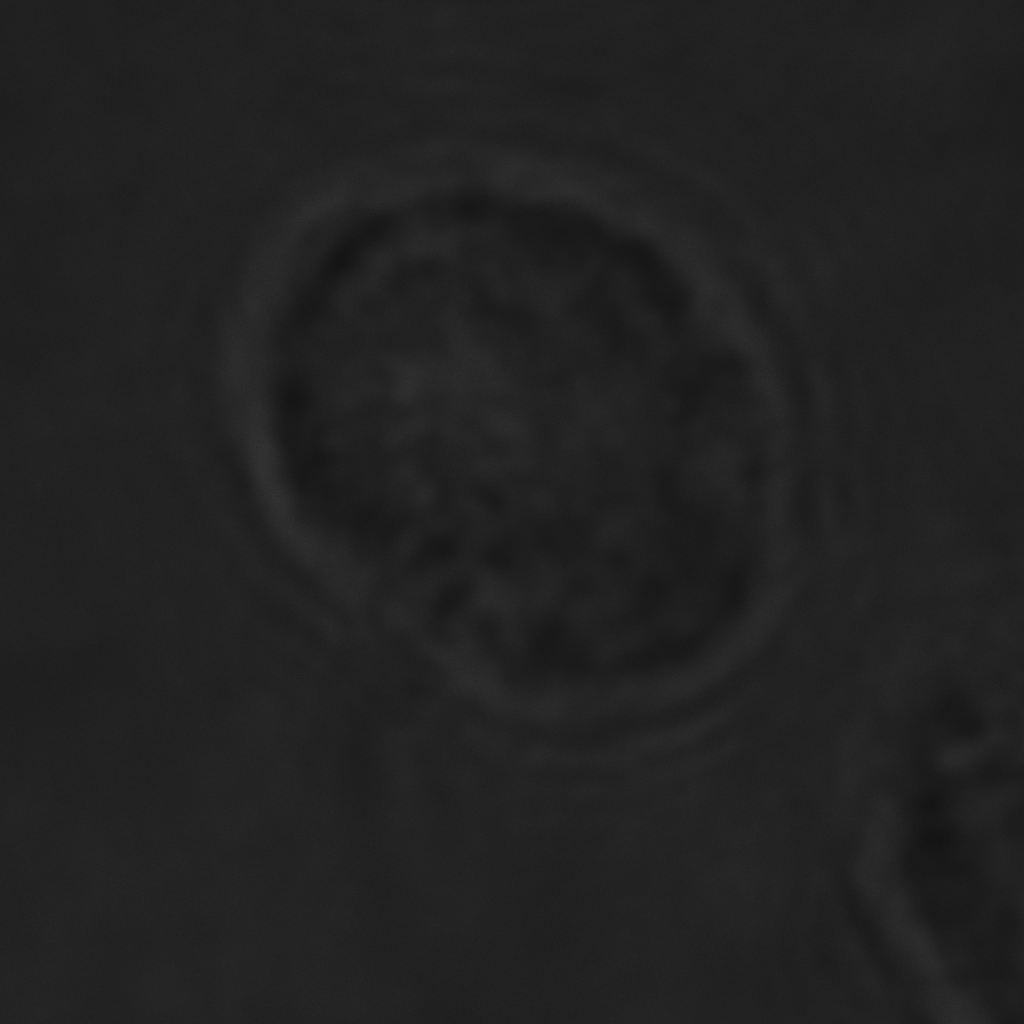

Supplement: Supplementary file 7 — Source Data Fig. 4 [file 44319_2023_11_MOESM7_ESM.zip › FIG 4/Figure 4B/ESYT1 KO/Resting/2023-06-30 NB Jurkat cells ESyt manuscript revisions001_2023-06-30-ESyt1KO Jurkat-GFP-DAG-Cy3-CD4-Cy5-pLAT-Resting017_ch01.tif]

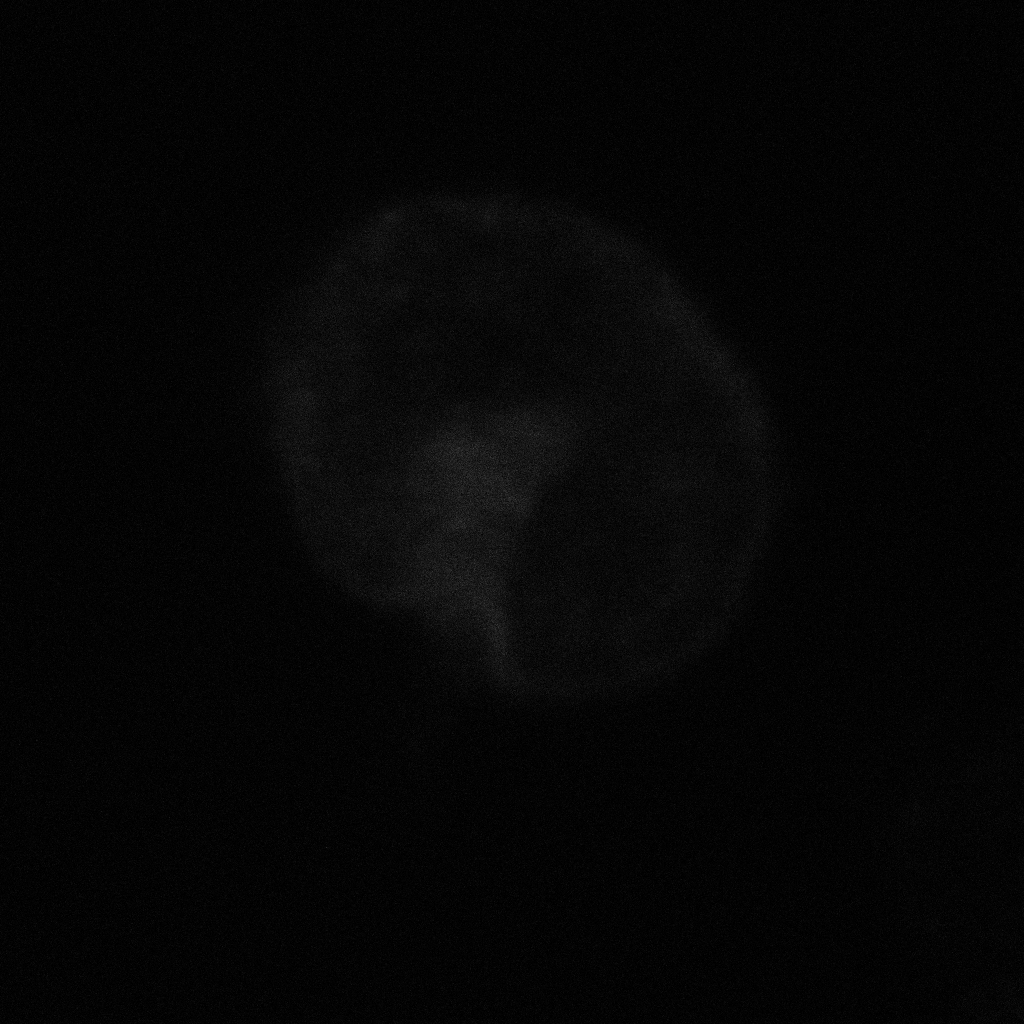

Supplement: Supplementary file 7 — Source Data Fig. 4 [file 44319_2023_11_MOESM7_ESM.zip › FIG 4/Figure 4B/ESYT1 KO/Resting/2023-06-30 NB Jurkat cells ESyt manuscript revisions001_2023-06-30-ESyt1KO Jurkat-GFP-DAG-Cy3-CD4-Cy5-pLAT-Resting017_ch00.tif]

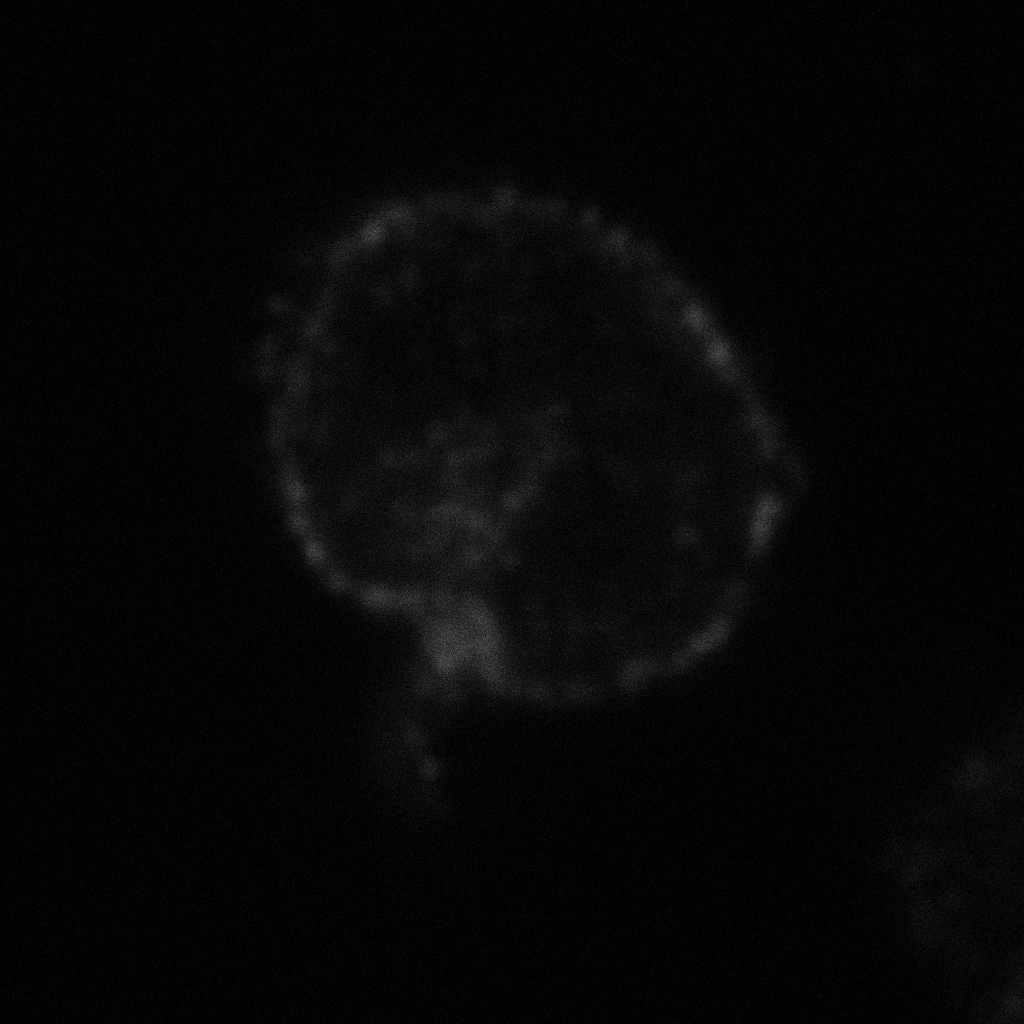

Supplement: Supplementary file 7 — Source Data Fig. 4 [file 44319_2023_11_MOESM7_ESM.zip › FIG 4/Figure 4B/ESYT1 KO/Resting/2023-06-30 NB Jurkat cells ESyt manuscript revisions001_2023-06-30-ESyt1KO Jurkat-GFP-DAG-Cy3-CD4-Cy5-pLAT-Resting017_ch02.tif]

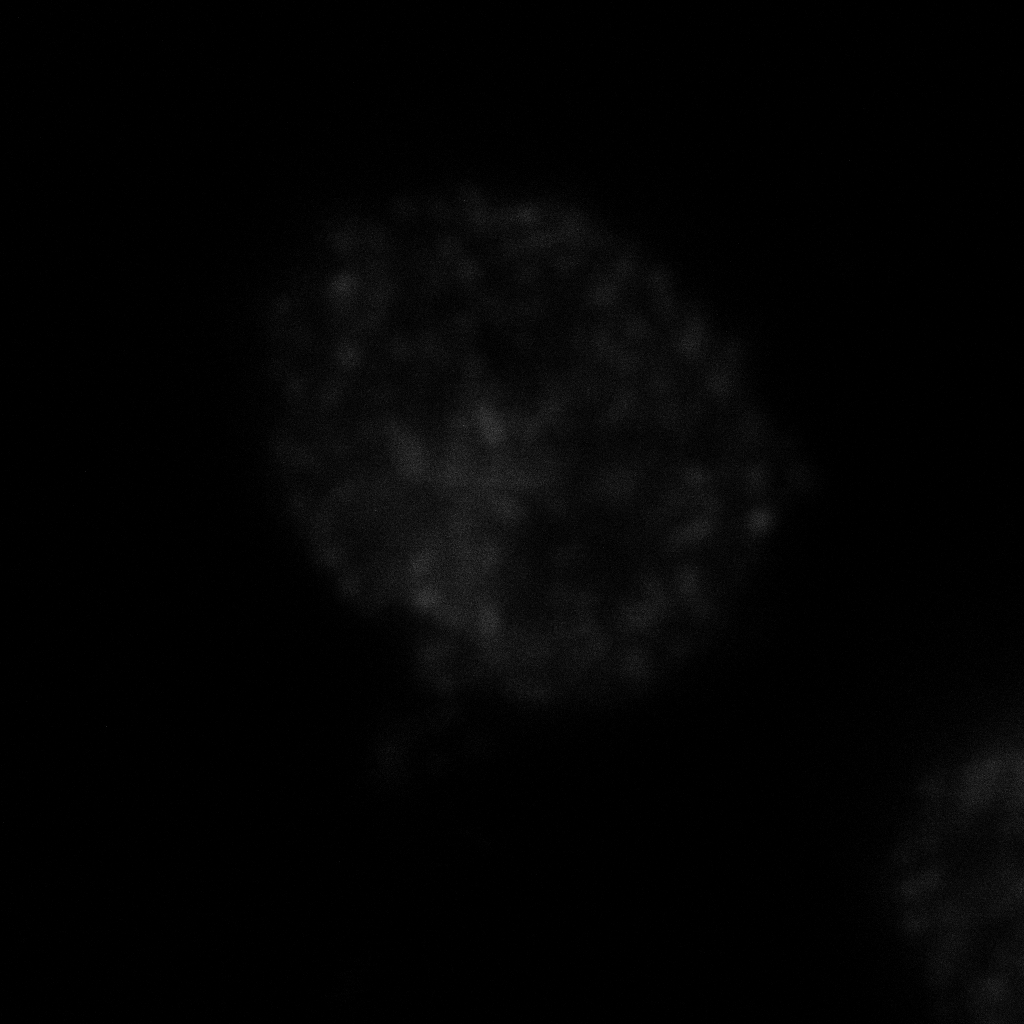

Supplement: Supplementary file 7 — Source Data Fig. 4 [file 44319_2023_11_MOESM7_ESM.zip › FIG 4/Figure 4B/ESYT1 KO/Resting/2023-06-30 NB Jurkat cells ESyt manuscript revisions001_2023-06-30-ESyt1KO Jurkat-GFP-DAG-Cy3-CD4-Cy5-pLAT-Resting017_ch03.tif]

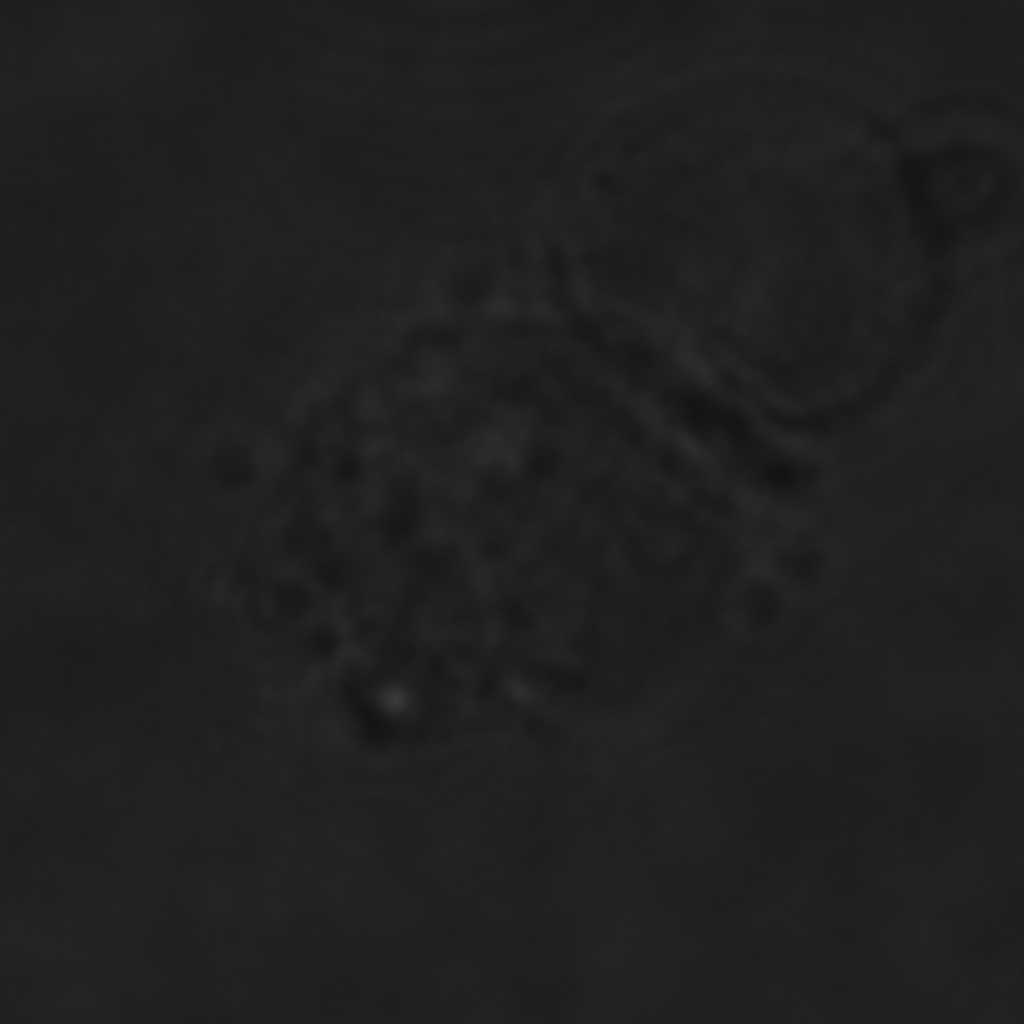

Supplement: Supplementary file 7 — Source Data Fig. 4 [file 44319_2023_11_MOESM7_ESM.zip › FIG 4/Figure 4B/WT Jurkat/Activated/2023-06-30 NB Jurkat cells ESyt manuscript revisions001_2023-06-30-WT Jurkat-GFP-DAG-Cy3-CD4-Cy5-pLAT-Activated015_ch01.tif]

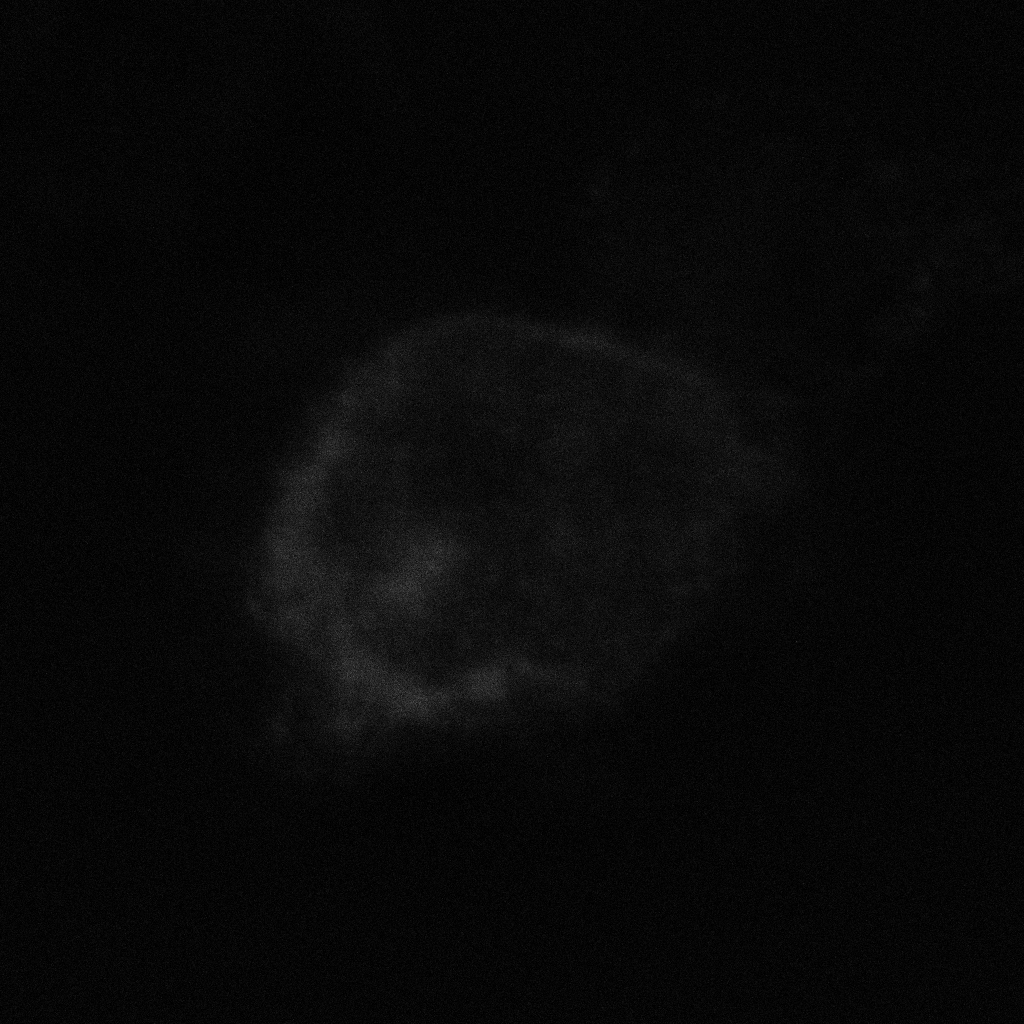

Supplement: Supplementary file 7 — Source Data Fig. 4 [file 44319_2023_11_MOESM7_ESM.zip › FIG 4/Figure 4B/WT Jurkat/Activated/2023-06-30 NB Jurkat cells ESyt manuscript revisions001_2023-06-30-WT Jurkat-GFP-DAG-Cy3-CD4-Cy5-pLAT-Activated015_ch00.tif]

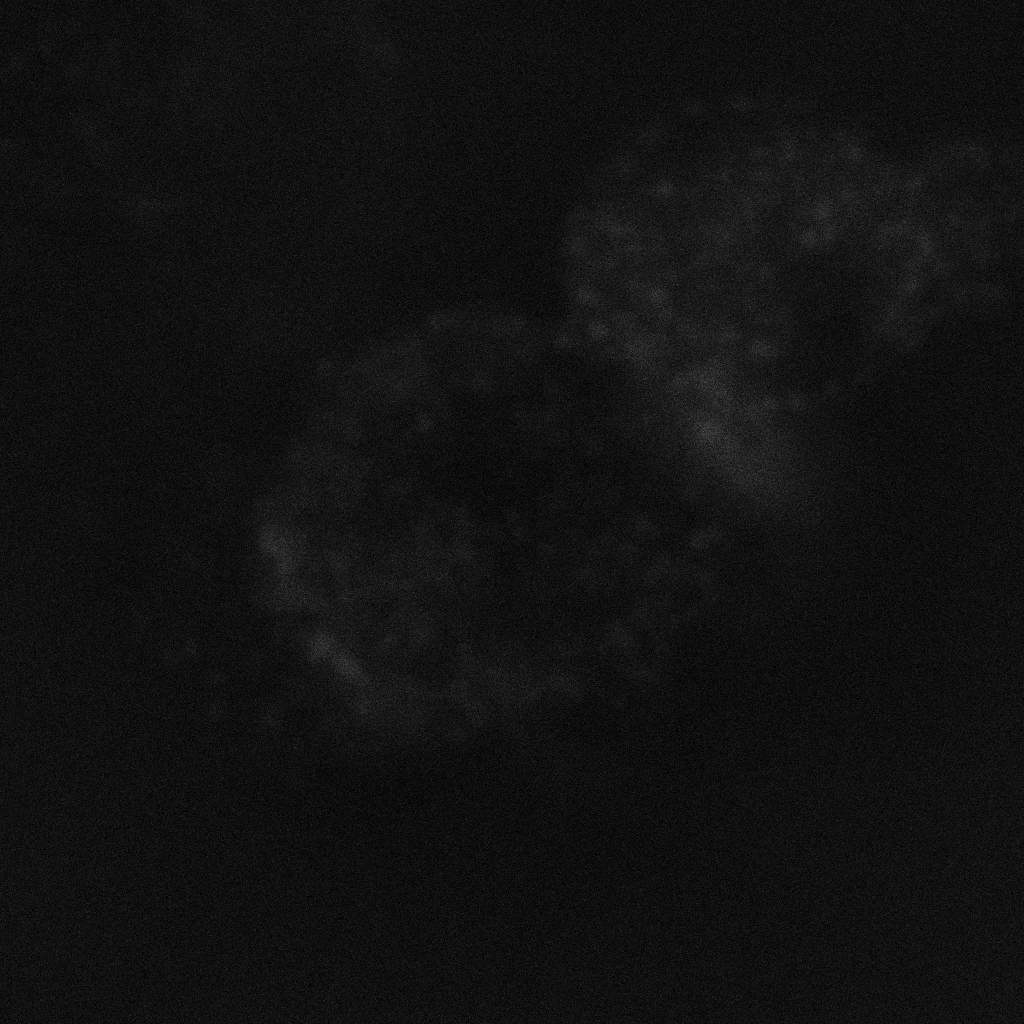

Supplement: Supplementary file 7 — Source Data Fig. 4 [file 44319_2023_11_MOESM7_ESM.zip › FIG 4/Figure 4B/WT Jurkat/Activated/2023-06-30 NB Jurkat cells ESyt manuscript revisions001_2023-06-30-WT Jurkat-GFP-DAG-Cy3-CD4-Cy5-pLAT-Activated015_ch02.tif]

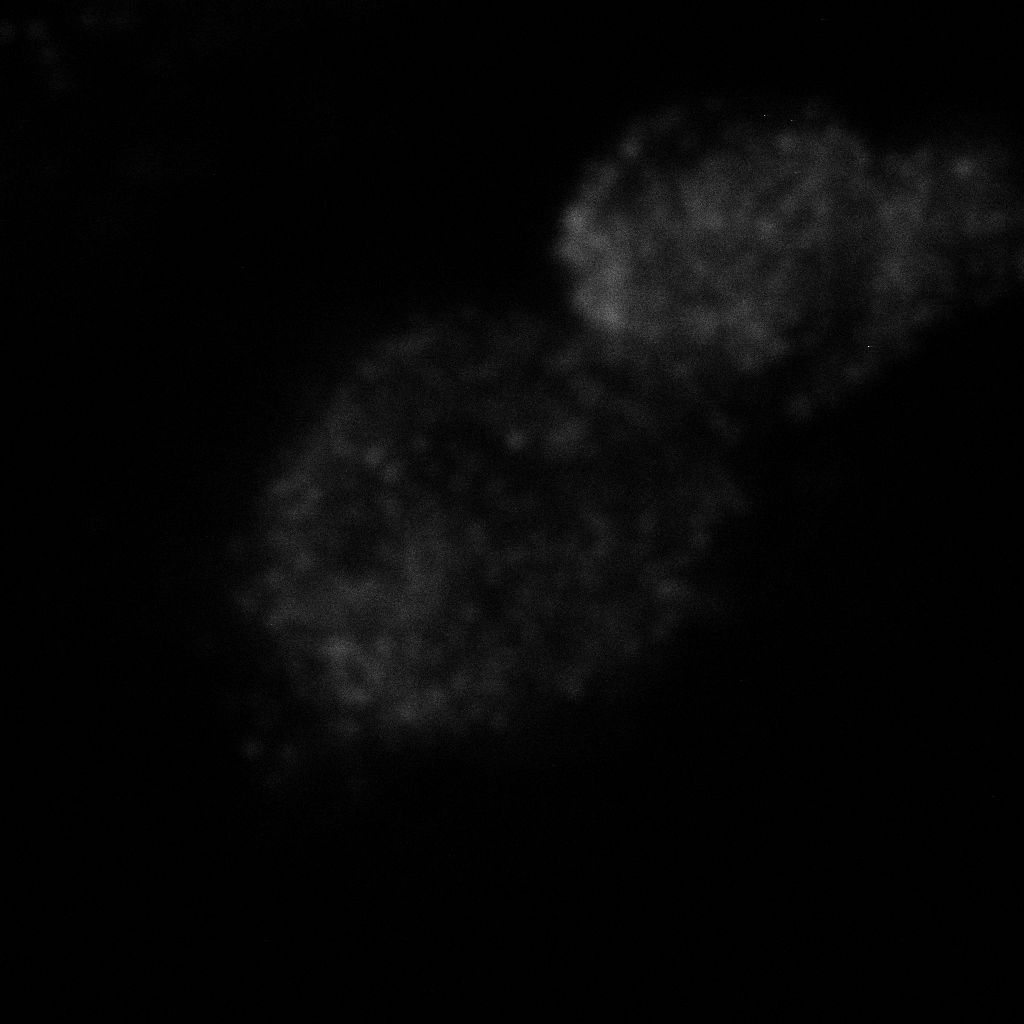

Supplement: Supplementary file 7 — Source Data Fig. 4 [file 44319_2023_11_MOESM7_ESM.zip › FIG 4/Figure 4B/WT Jurkat/Activated/2023-06-30 NB Jurkat cells ESyt manuscript revisions001_2023-06-30-WT Jurkat-GFP-DAG-Cy3-CD4-Cy5-pLAT-Activated015_ch03.tif]

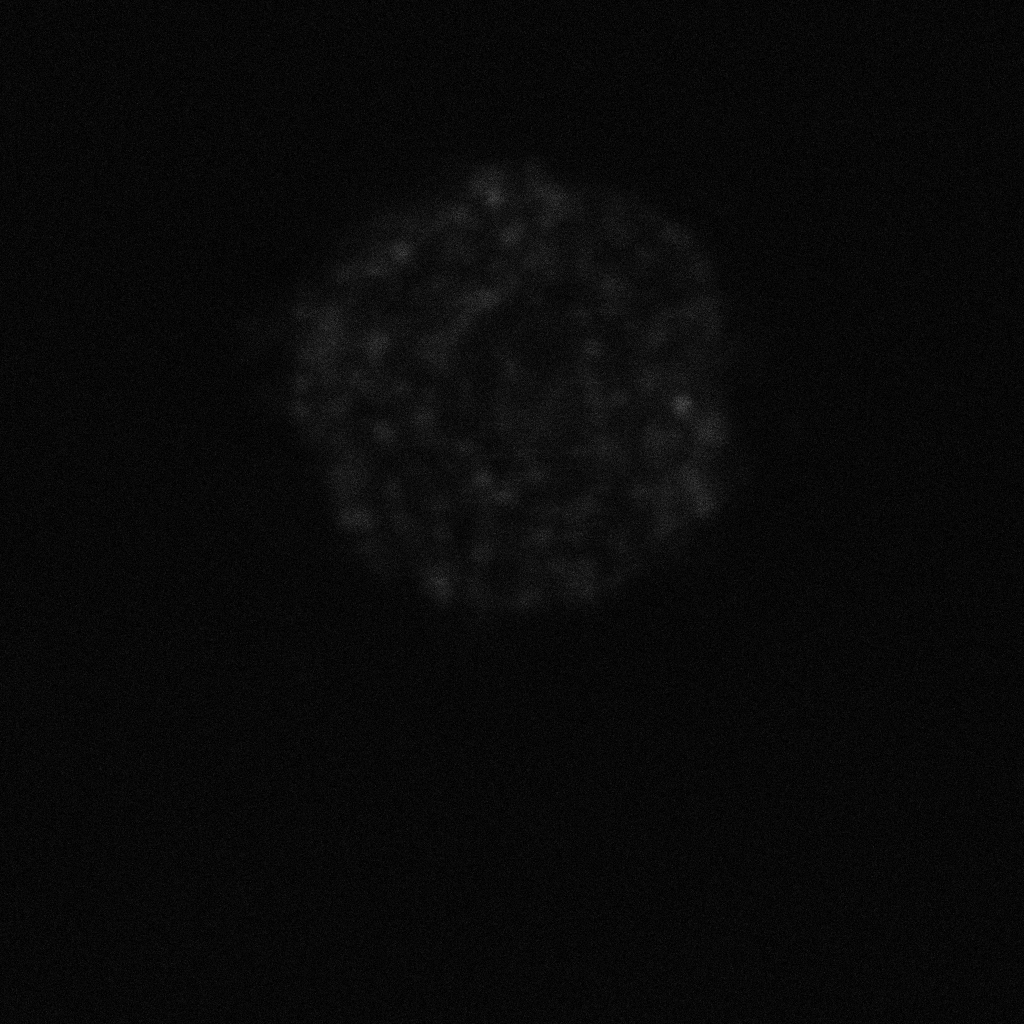

Supplement: Supplementary file 7 — Source Data Fig. 4 [file 44319_2023_11_MOESM7_ESM.zip › FIG 4/Figure 4B/WT Jurkat/Resting/2023-06-30 NB Jurkat cells ESyt manuscript revisions001_2023-06-30-WT Jurkat-GFP-DAG-Cy3-CD4-Cy5-pLAT-Resting004_ch02.tif]

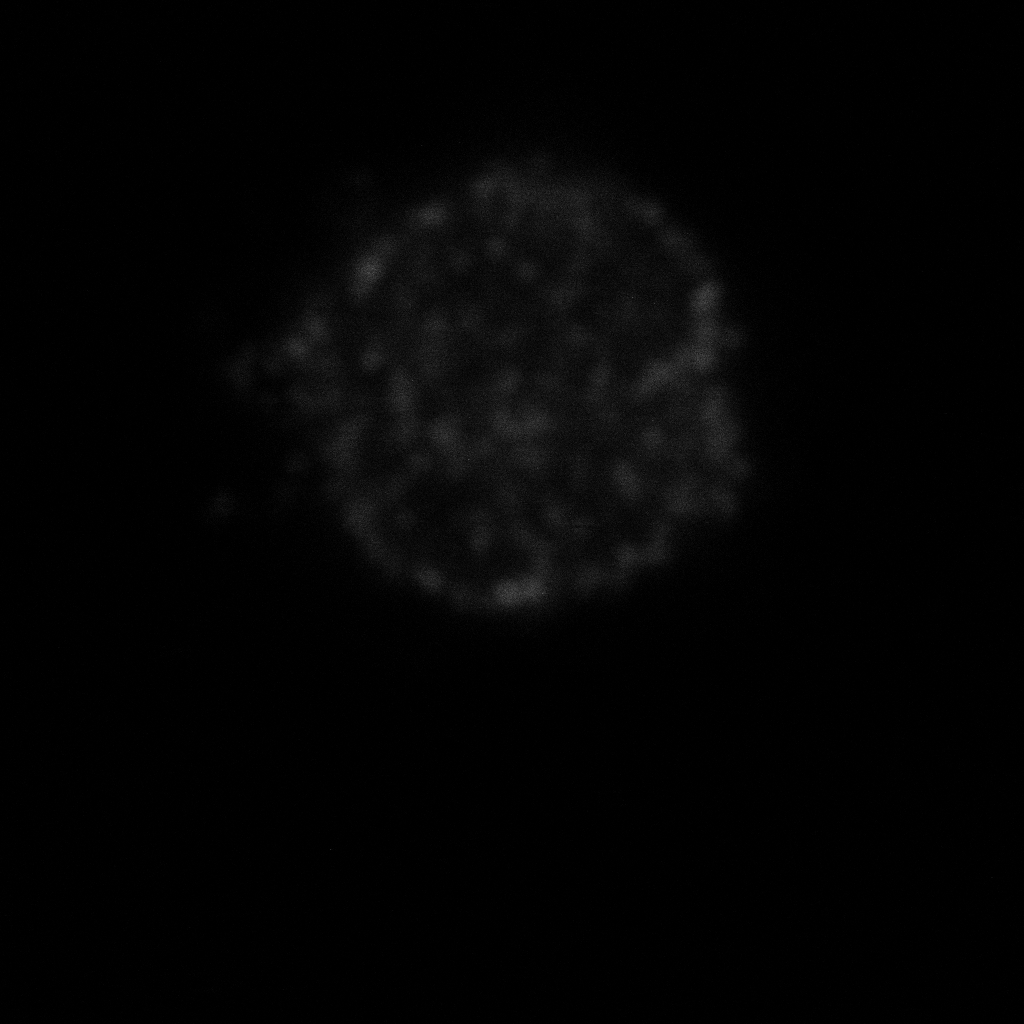

Supplement: Supplementary file 7 — Source Data Fig. 4 [file 44319_2023_11_MOESM7_ESM.zip › FIG 4/Figure 4B/WT Jurkat/Resting/2023-06-30 NB Jurkat cells ESyt manuscript revisions001_2023-06-30-WT Jurkat-GFP-DAG-Cy3-CD4-Cy5-pLAT-Resting004_ch03.tif]

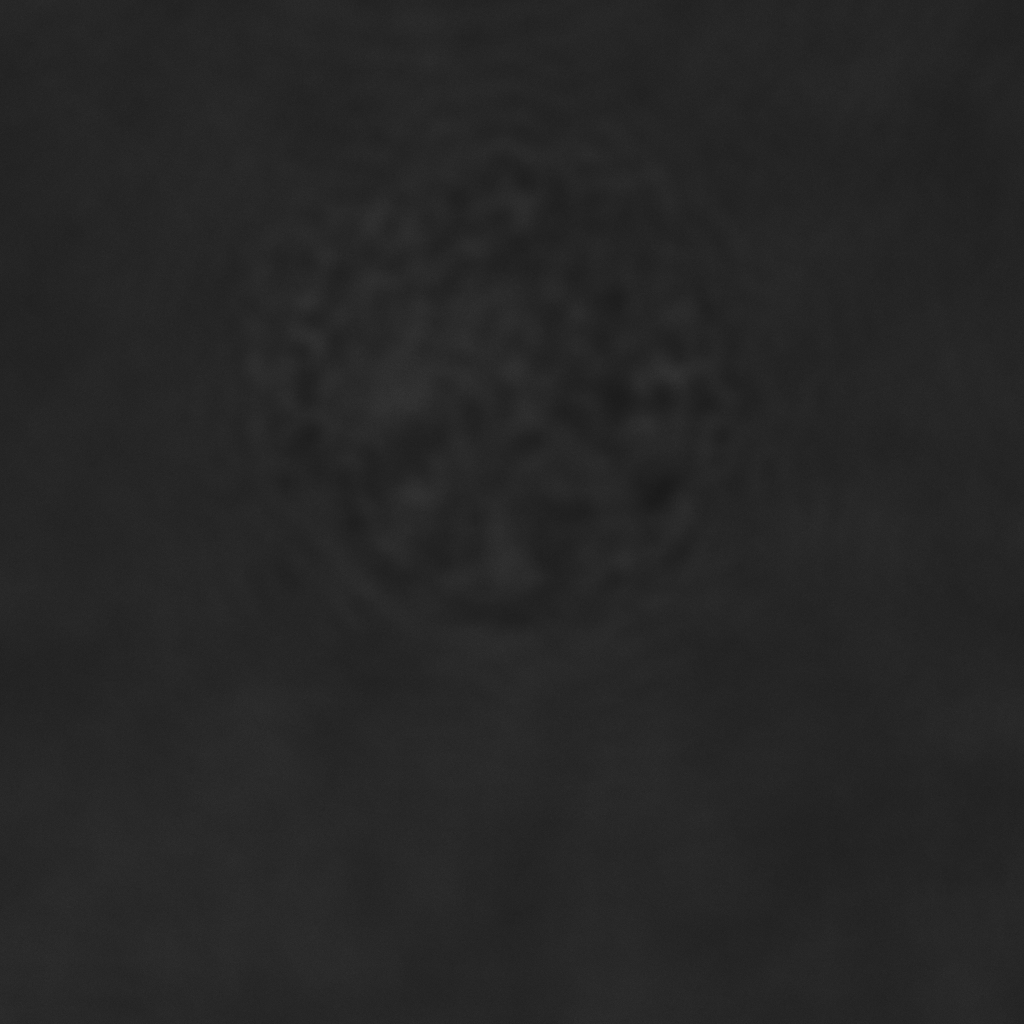

Supplement: Supplementary file 7 — Source Data Fig. 4 [file 44319_2023_11_MOESM7_ESM.zip › FIG 4/Figure 4B/WT Jurkat/Resting/2023-06-30 NB Jurkat cells ESyt manuscript revisions001_2023-06-30-WT Jurkat-GFP-DAG-Cy3-CD4-Cy5-pLAT-Resting004_ch01.tif]

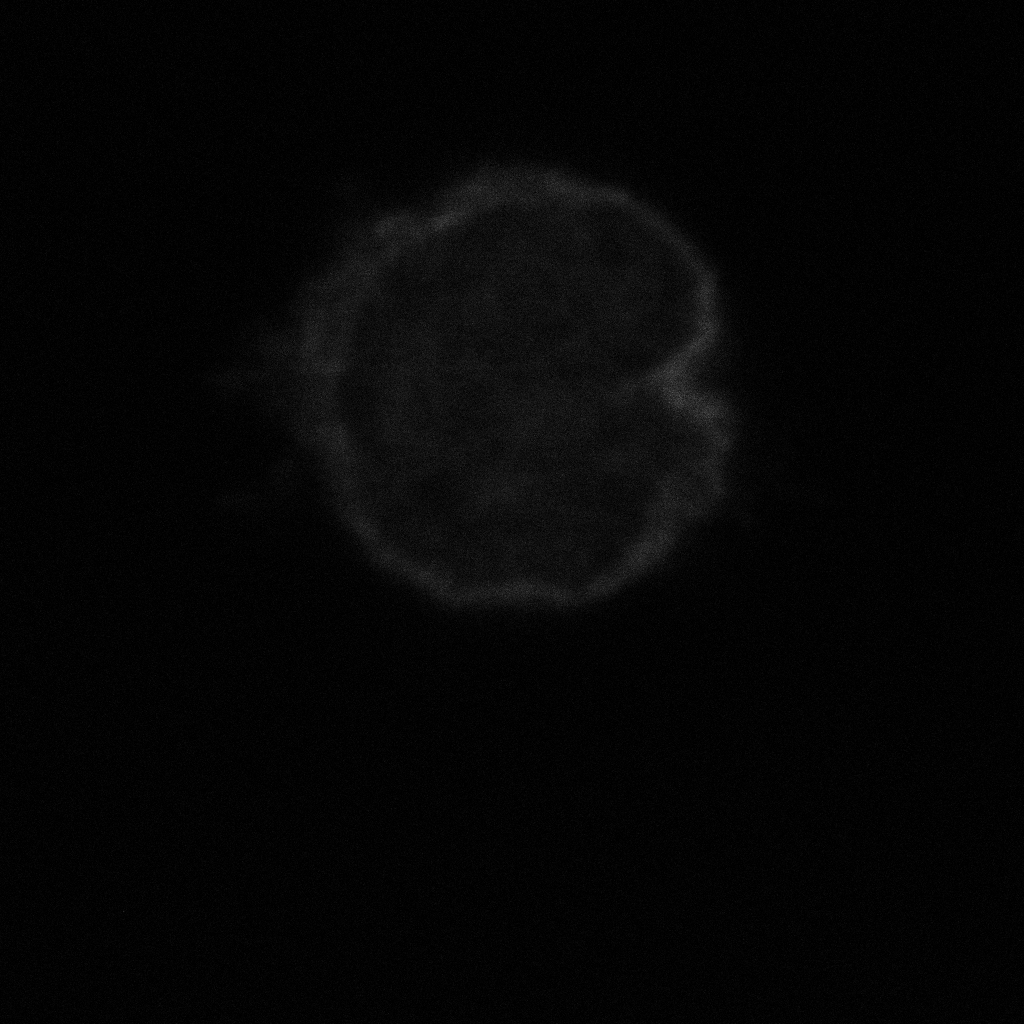

Supplement: Supplementary file 7 — Source Data Fig. 4 [file 44319_2023_11_MOESM7_ESM.zip › FIG 4/Figure 4B/WT Jurkat/Resting/2023-06-30 NB Jurkat cells ESyt manuscript revisions001_2023-06-30-WT Jurkat-GFP-DAG-Cy3-CD4-Cy5-pLAT-Resting004_ch00.tif]

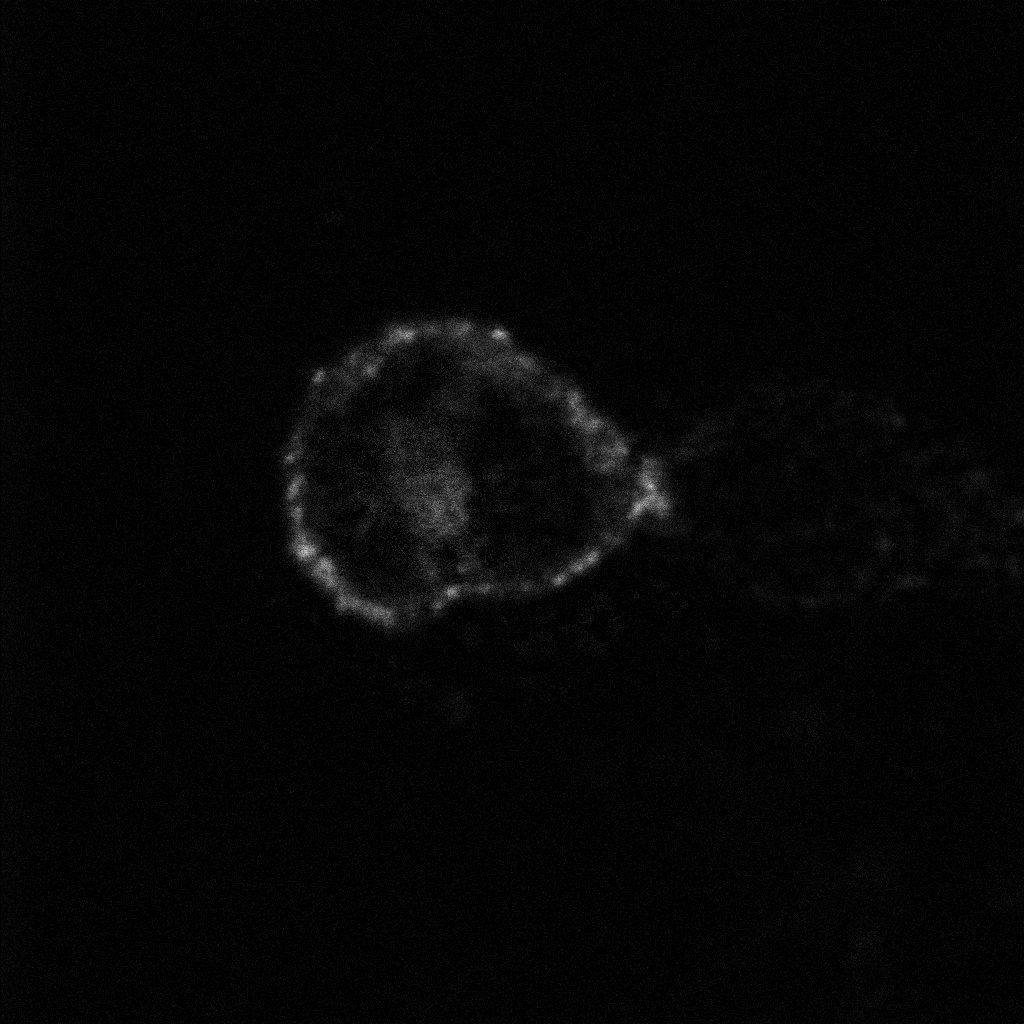

Supplement: Supplementary file 7 — Source Data Fig. 4 [file 44319_2023_11_MOESM7_ESM.zip › FIG 4/Figure 4A/WT Jurkat GFP DAG mCherry ESYT2 Cy5 CD4/Activated/2023-08-21-NB-Jurkat-ESYT -cell markers 2_2023-08-22-WT-Jurkat-GFP-DAG-mCherry-ESyt2-Cy5-CD4-Activated006 ╧ä-STED1_ch01.tif]

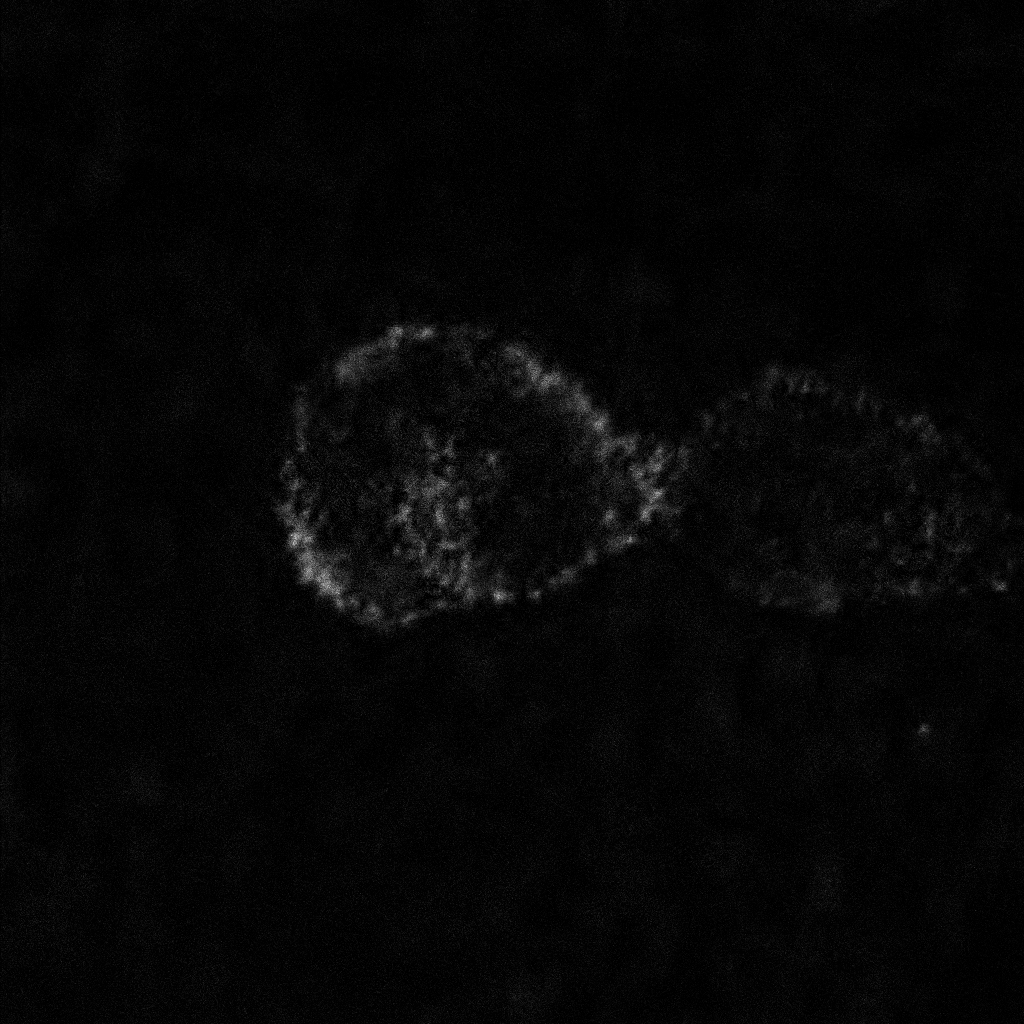

Supplement: Supplementary file 7 — Source Data Fig. 4 [file 44319_2023_11_MOESM7_ESM.zip › FIG 4/Figure 4A/WT Jurkat GFP DAG mCherry ESYT2 Cy5 CD4/Activated/2023-08-21-NB-Jurkat-ESYT -cell markers 2_2023-08-22-WT-Jurkat-GFP-DAG-mCherry-ESyt2-Cy5-CD4-Activated006 ╧ä-STED1_ch00.tif]

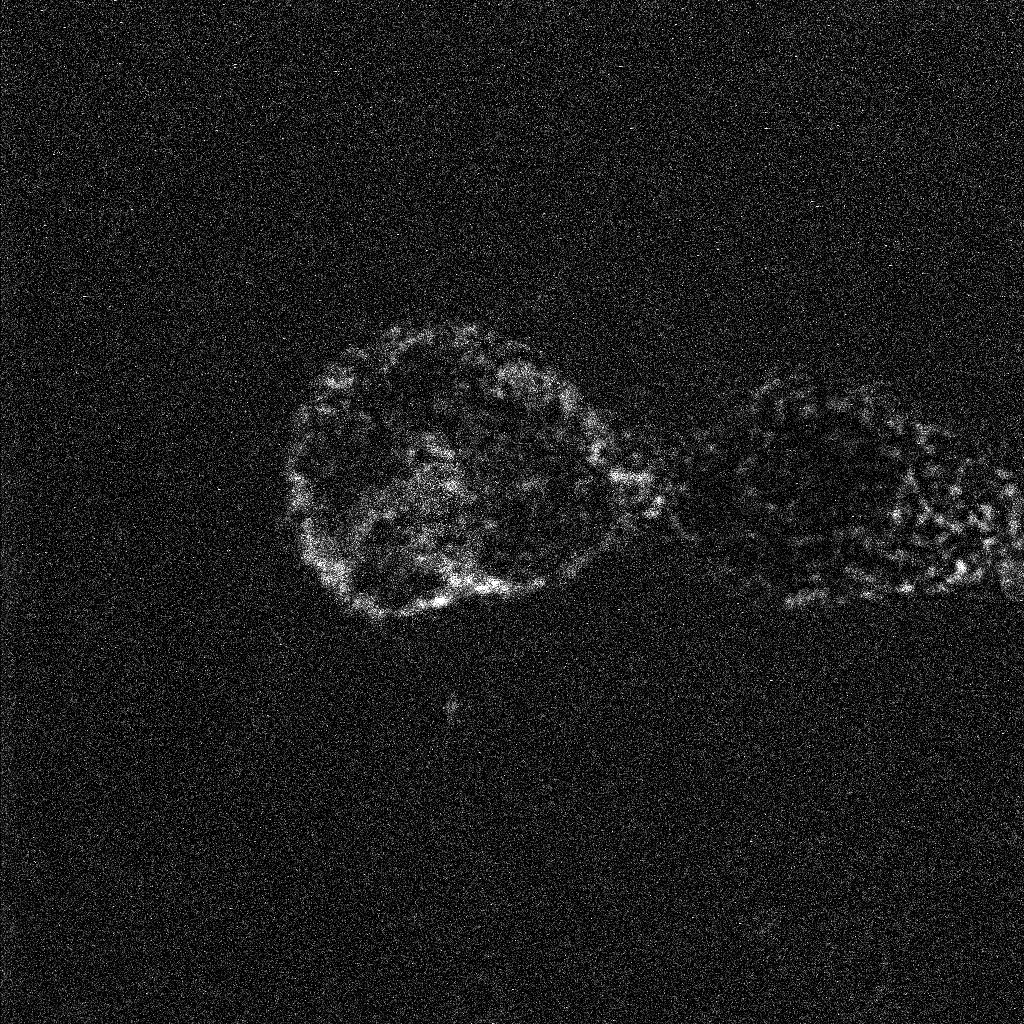

Supplement: Supplementary file 7 — Source Data Fig. 4 [file 44319_2023_11_MOESM7_ESM.zip › FIG 4/Figure 4A/WT Jurkat GFP DAG mCherry ESYT2 Cy5 CD4/Activated/2023-08-21-NB-Jurkat-ESYT -cell markers 2_2023-08-22-WT-Jurkat-GFP-DAG-mCherry-ESyt2-Cy5-CD4-Activated006 ╧ä-STED1_ch02.tif]

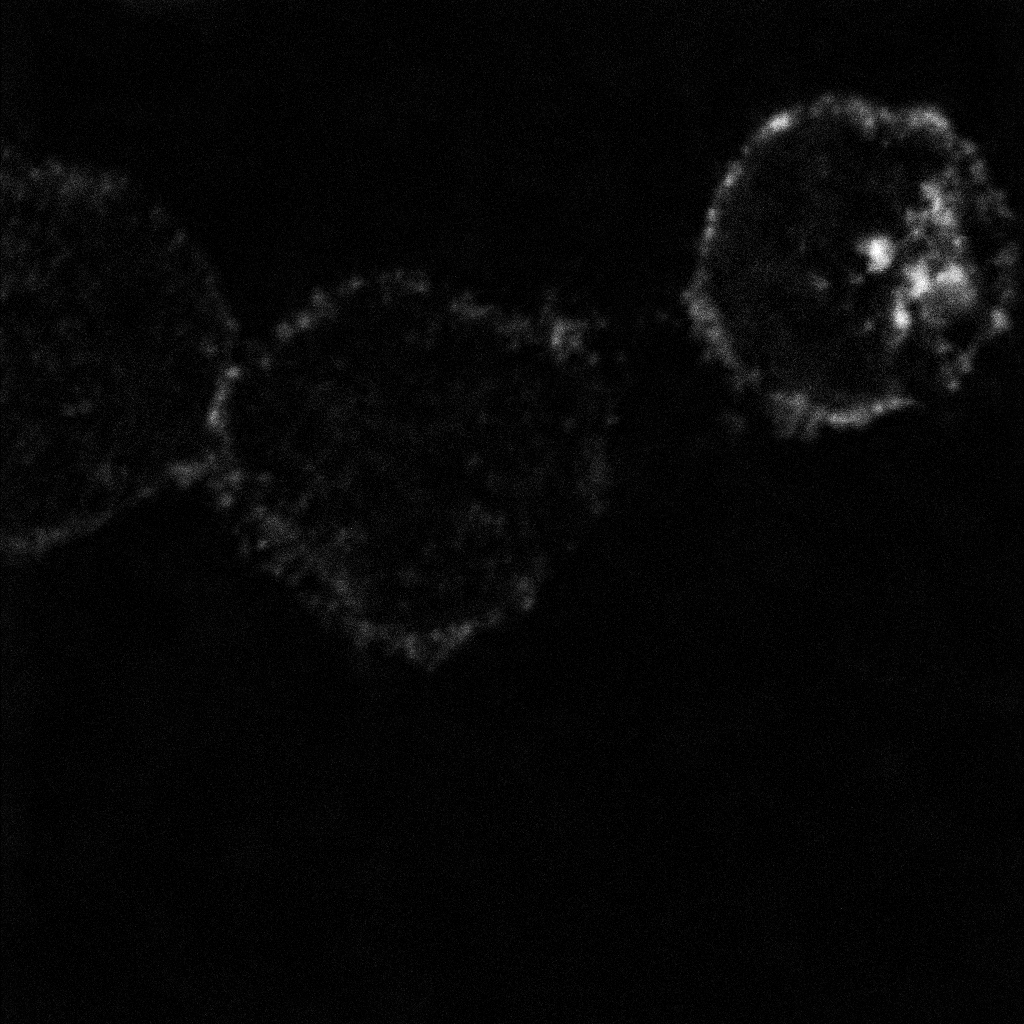

Supplement: Supplementary file 7 — Source Data Fig. 4 [file 44319_2023_11_MOESM7_ESM.zip › FIG 4/Figure 4A/WT Jurkat GFP DAG mCherry ESYT2 Cy5 CD4/Resting/2023-08-21-NB-Jurkat-ESYT -cell markers 2-2023-08-22-WT-Jurkat-GFP-DAG-mCherry-ESyt2-Cy5-CD4-Resting005_FLIM ╧ä-STED3_ch00.tif]

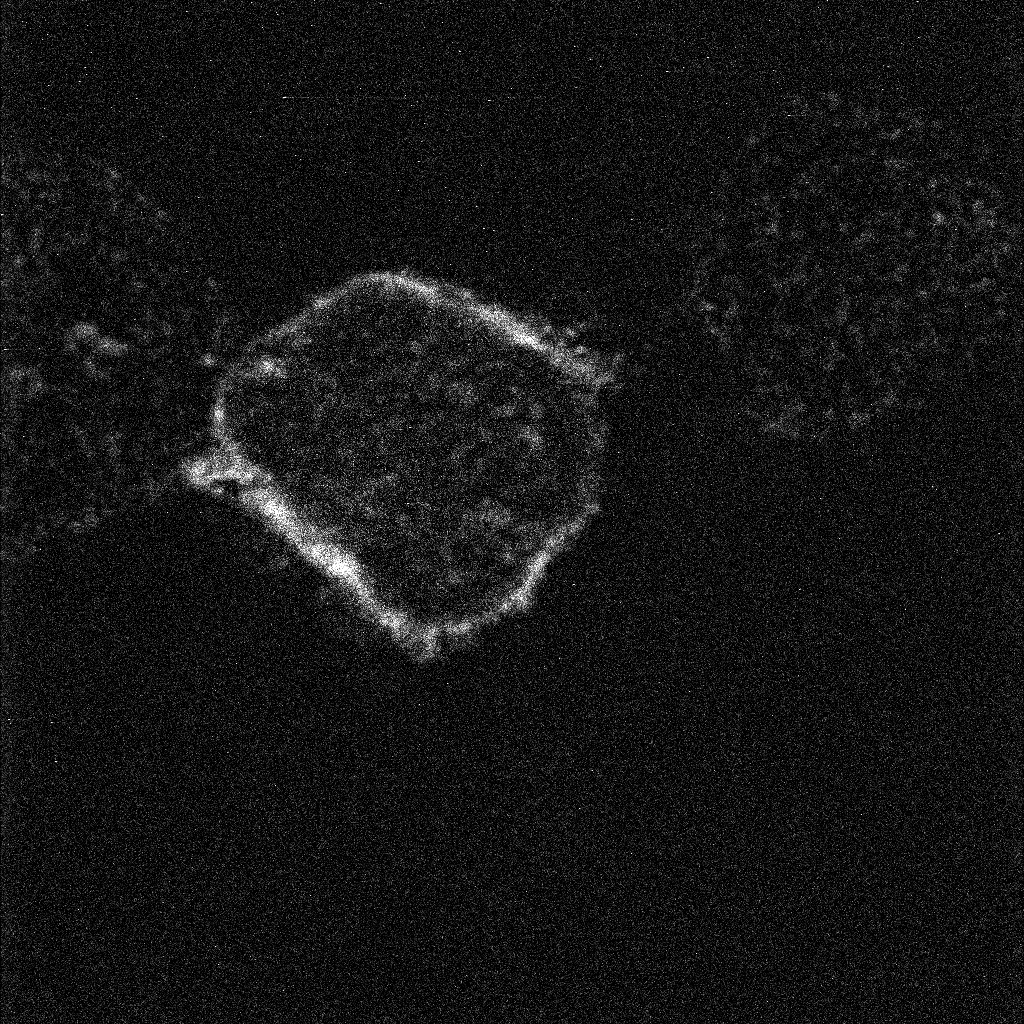

Supplement: Supplementary file 7 — Source Data Fig. 4 [file 44319_2023_11_MOESM7_ESM.zip › FIG 4/Figure 4A/WT Jurkat GFP DAG mCherry ESYT2 Cy5 CD4/Resting/2023-08-21-NB-Jurkat-ESYT -cell markers 2_ 2023-08-22-WT-Jurkat-GFP-DAG-mCherry-ESyt2-Cy5-Cy5-CD4-Resting005FLIM ╧ä-STED3_ch02.tif]

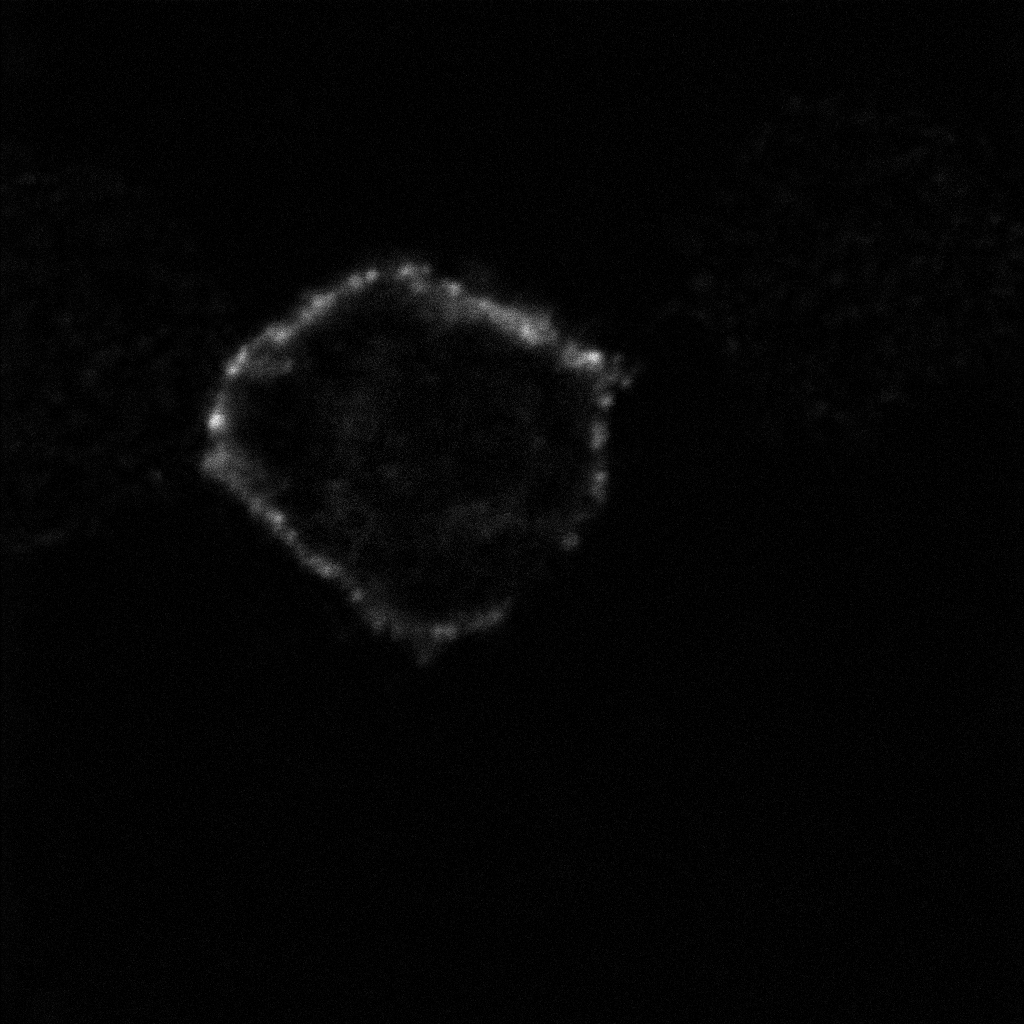

Supplement: Supplementary file 7 — Source Data Fig. 4 [file 44319_2023_11_MOESM7_ESM.zip › FIG 4/Figure 4A/WT Jurkat GFP DAG mCherry ESYT2 Cy5 CD4/Resting/2023-08-21-NB-Jurkat-ESYT -cell markers 22023-08-22-WT-Jrukat-GFP-DAG-mCherry-ESyt2-Cy5-CD4-Resting005_FLIM ╧ä-STED3_ch01.tif]

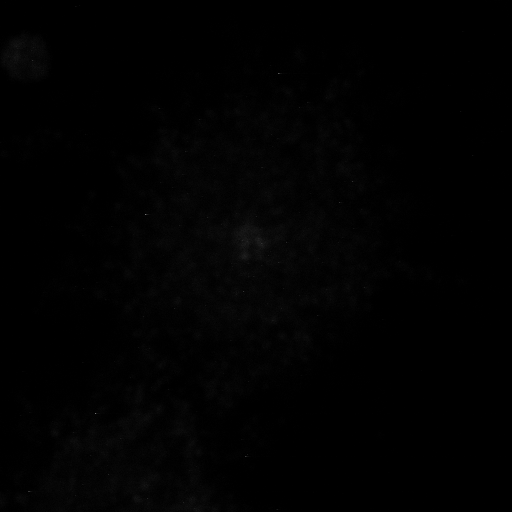

Supplement: Supplementary file 7 — Source Data Fig. 4 [file 44319_2023_11_MOESM7_ESM.zip › FIG 4/Figure 4A/WT Jurkat- GFP DAG mCherry ESYT1 Cy5 CD4/Activated/2021-06-17-N-Jurkat-Bioprobes-Constructs-STED2_2021-06-17-WT-Jurkat-GFP-DAG-mCherry-ESYT2-Cy5-CD4-Activated015_ch02.tif]

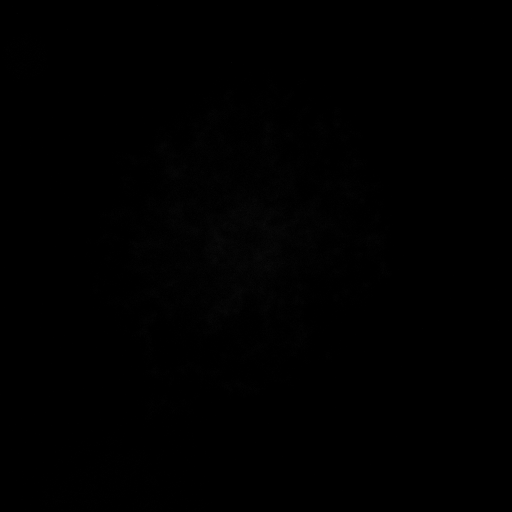

Supplement: Supplementary file 7 — Source Data Fig. 4 [file 44319_2023_11_MOESM7_ESM.zip › FIG 4/Figure 4A/WT Jurkat- GFP DAG mCherry ESYT1 Cy5 CD4/Activated/2021-06-17-N-Jurkat-Bioprobes-Constructs-STED2_2021-06-17-WT-Jurkat-GFP-DAG-mCherry-ESYT2-Cy5-CD4-Activated015_ch01.tif]

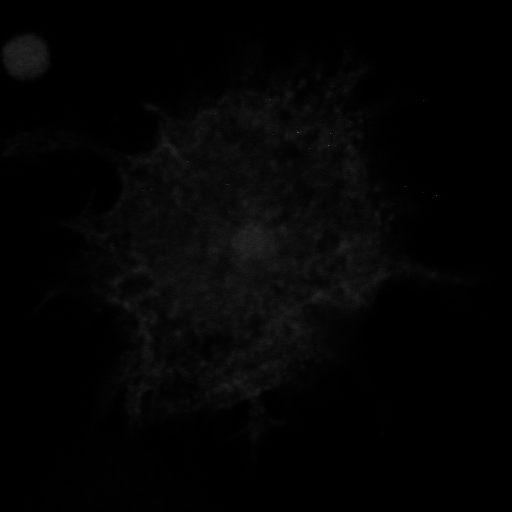

Supplement: Supplementary file 7 — Source Data Fig. 4 [file 44319_2023_11_MOESM7_ESM.zip › FIG 4/Figure 4A/WT Jurkat- GFP DAG mCherry ESYT1 Cy5 CD4/Activated/2021-06-17-N-Jurkat-Bioprobes-Constructs-STED2_2021-06-17-WT-Jurkat-GFP-DAG-mCherry-ESYT2-Cy5-CD4-Activated015_ch00.tif]

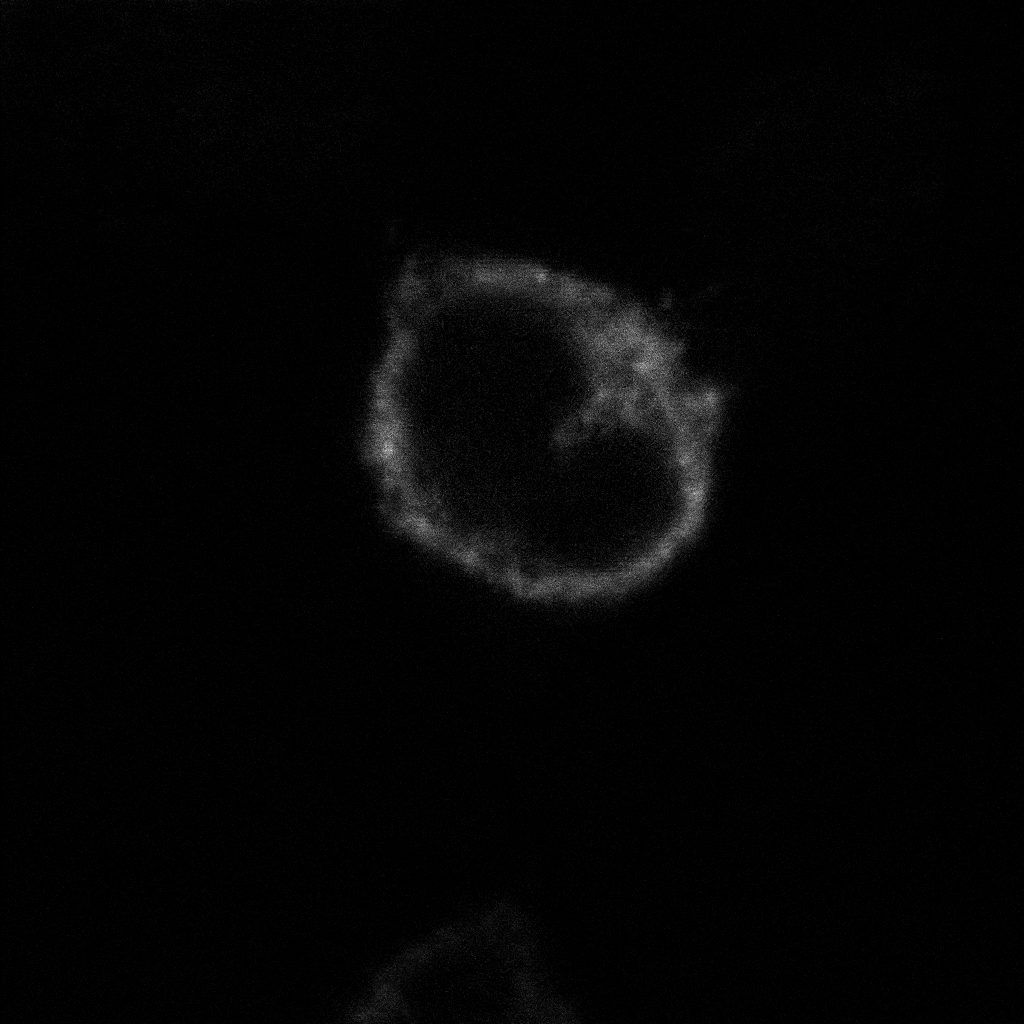

Supplement: Supplementary file 7 — Source Data Fig. 4 [file 44319_2023_11_MOESM7_ESM.zip › FIG 4/Figure 4A/WT Jurkat- GFP DAG mCherry ESYT1 Cy5 CD4/Resting/2023-08-21-NB-Jurkat-ESYT -cell markers 2_2023-08-23-WT-Jurkat-GFP-DAG-mCherry-ESYT1-Cy5-CD4-Resting003 ╧ä-STED1_ch01.tif]

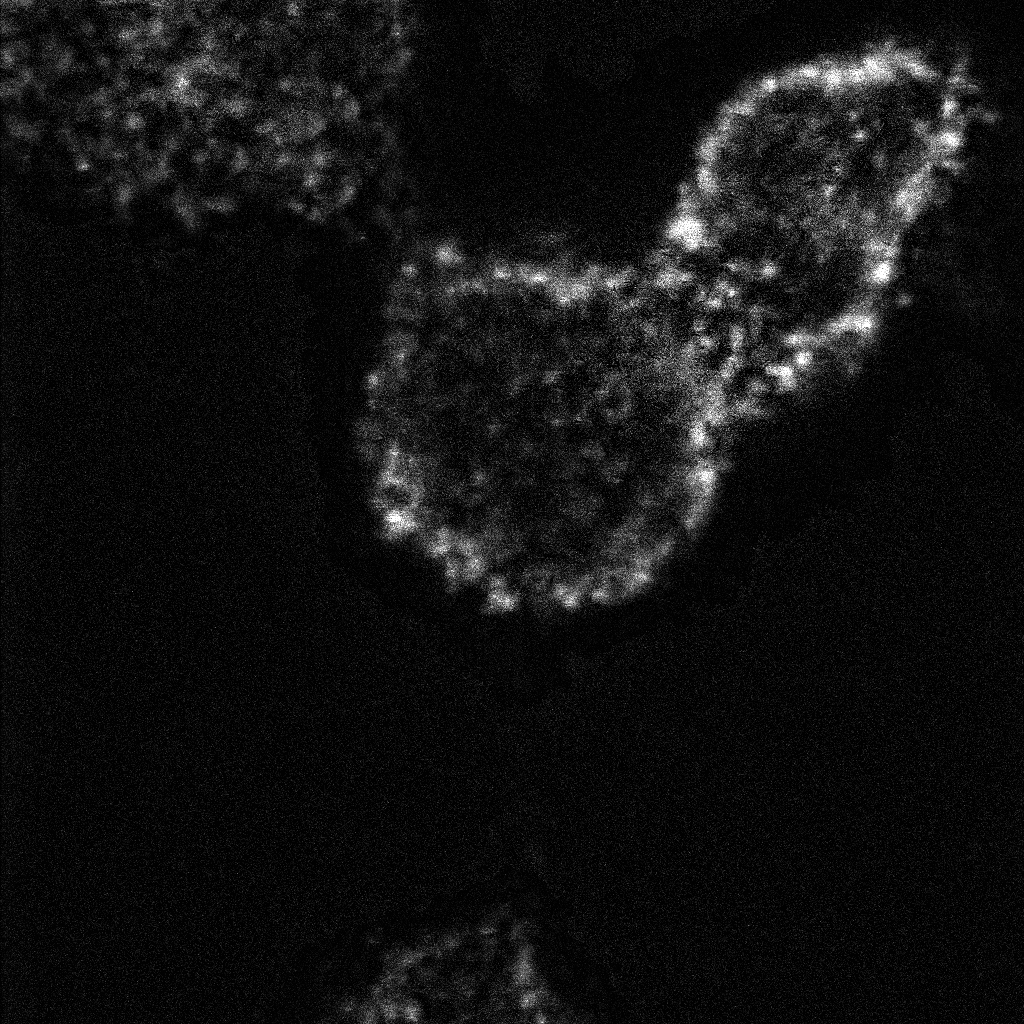

Supplement: Supplementary file 7 — Source Data Fig. 4 [file 44319_2023_11_MOESM7_ESM.zip › FIG 4/Figure 4A/WT Jurkat- GFP DAG mCherry ESYT1 Cy5 CD4/Resting/2023-08-21-NB-Jurkat-ESYT -cell markers 2_2023-08-23-WT-Jurkat-GFP-DAG-mCherry-ESYT1-Cy5-CD4-Resting003 ╧ä-STED1_ch00.tif]

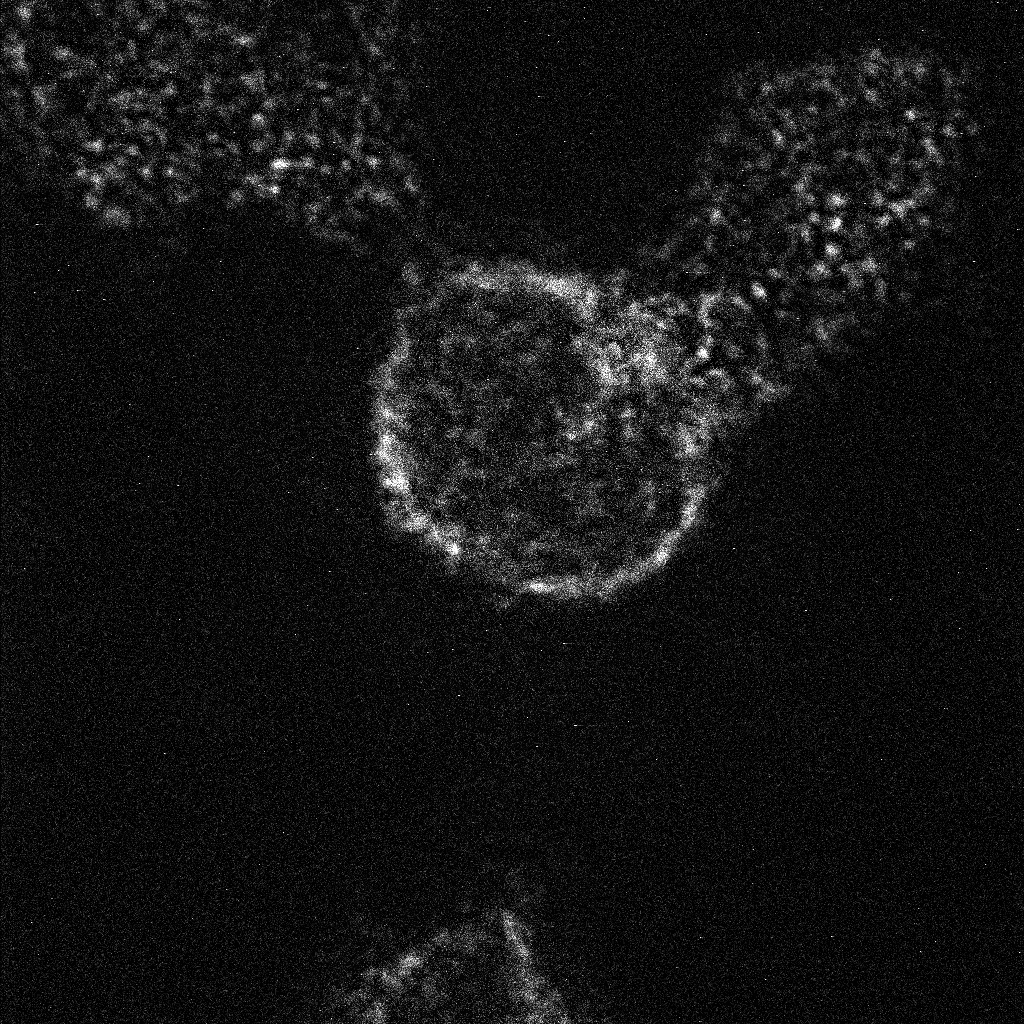

Supplement: Supplementary file 7 — Source Data Fig. 4 [file 44319_2023_11_MOESM7_ESM.zip › FIG 4/Figure 4A/WT Jurkat- GFP DAG mCherry ESYT1 Cy5 CD4/Resting/2023-08-21-NB-Jurkat-ESYT -cell markers 2_2023-08-23-WT-Jurkat-GFP-DAG-mCherry-ESYT1-Cy5-CD4-Resting003 ╧ä-STED1_ch02.tif]

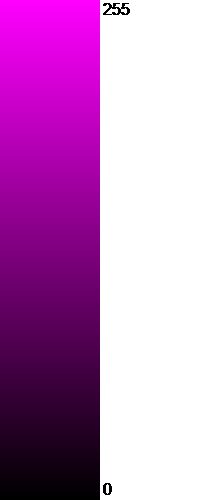

Supplement: Supplementary file 7 — Source Data Fig. 4 [file 44319_2023_11_MOESM7_ESM.zip › FIG 4/Figure 4B/ESYT1&2 DKO/Activated/MetaData/2023-06-30 NB Jurkat cells ESyt manuscript revisions001_2023-07-05-ESyt1&@DKO-GFP-DAG-Cy3-CD4-Cy5-pLAT-Activated024ch3LUT.png]

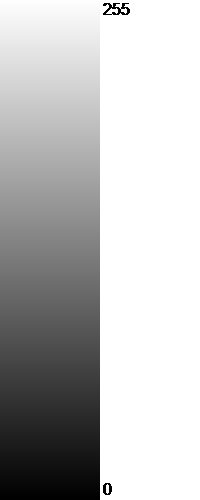

Supplement: Supplementary file 7 — Source Data Fig. 4 [file 44319_2023_11_MOESM7_ESM.zip › FIG 4/Figure 4B/ESYT1&2 DKO/Activated/MetaData/2023-06-30 NB Jurkat cells ESyt manuscript revisions001_2023-07-05-ESyt1&@DKO-GFP-DAG-Cy3-CD4-Cy5-pLAT-Activated024ch1LUT.png]

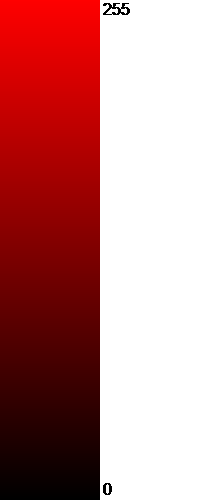

Supplement: Supplementary file 7 — Source Data Fig. 4 [file 44319_2023_11_MOESM7_ESM.zip › FIG 4/Figure 4B/ESYT1&2 DKO/Activated/MetaData/2023-06-30 NB Jurkat cells ESyt manuscript revisions001_2023-07-05-ESyt1&@DKO-GFP-DAG-Cy3-CD4-Cy5-pLAT-Activated024ch2LUT.png]

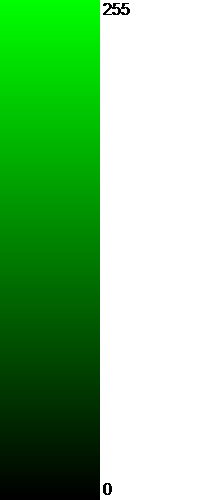

Supplement: Supplementary file 7 — Source Data Fig. 4 [file 44319_2023_11_MOESM7_ESM.zip › FIG 4/Figure 4B/ESYT1&2 DKO/Activated/MetaData/2023-06-30 NB Jurkat cells ESyt manuscript revisions001_2023-07-05-ESyt1&@DKO-GFP-DAG-Cy3-CD4-Cy5-pLAT-Activated024ch0LUT.png]

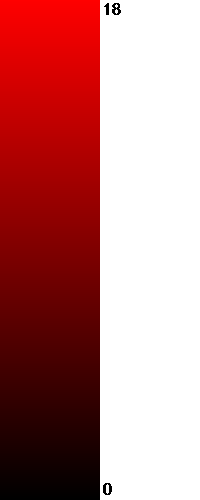

Supplement: Supplementary file 7 — Source Data Fig. 4 [file 44319_2023_11_MOESM7_ESM.zip › FIG 4/Figure 4A/WT Jurkat- GFP DAG mCherry ESYT1 Cy5 CD4/Activated/MetaData/2021-06-17-N-Jurkat-Bioprobes-Constructs-STED2_2021-06-17-WT-Jurkat-GFP-DAG-mCherry-ESYT2-Cy5-CD4-Activated015ch1LUT.png]

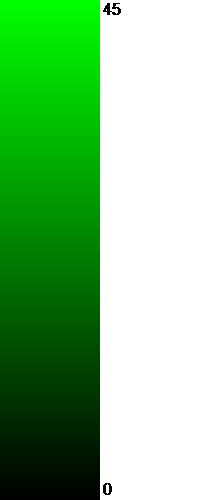

Supplement: Supplementary file 7 — Source Data Fig. 4 [file 44319_2023_11_MOESM7_ESM.zip › FIG 4/Figure 4A/WT Jurkat- GFP DAG mCherry ESYT1 Cy5 CD4/Activated/MetaData/2021-06-17-N-Jurkat-Bioprobes-Constructs-STED2_2021-06-17-WT-Jurkat-GFP-DAG-mCherry-ESYT2-Cy5-CD4-Activated015ch0LUT.png]

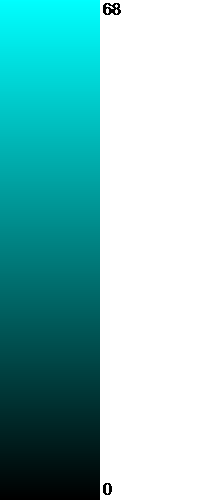

Supplement: Supplementary file 7 — Source Data Fig. 4 [file 44319_2023_11_MOESM7_ESM.zip › FIG 4/Figure 4A/WT Jurkat- GFP DAG mCherry ESYT1 Cy5 CD4/Activated/MetaData/2021-06-17-N-Jurkat-Bioprobes-Constructs-STED2_2021-06-17-WT-Jurkat-GFP-DAG-mCherry-ESYT2-Cy5-CD4-Activated015ch2LUT.png]

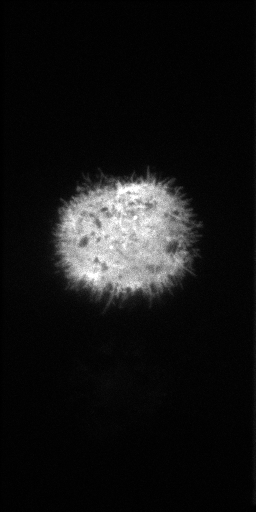

Supplement: Supplementary file 8 — Source Data Fig. 5 [file 44319_2023_11_MOESM8_ESM.zip › FIG 5/Figure 5C/Fig 5C. E-Syt1&2 DKO Jurkat cell activated conditions movie 4- TIRF/movie 4_E-Syt1&2 DKO Jurkat activated t=0.tif]

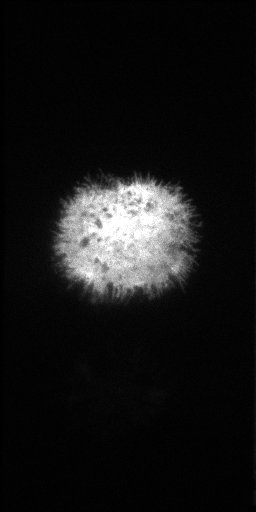

Supplement: Supplementary file 8 — Source Data Fig. 5 [file 44319_2023_11_MOESM8_ESM.zip › FIG 5/Figure 5C/Fig 5C. E-Syt1&2 DKO Jurkat cell activated conditions movie 4- TIRF/movie 4_E-Syt1&2 DKO Jurkat activated t=100.jpg]

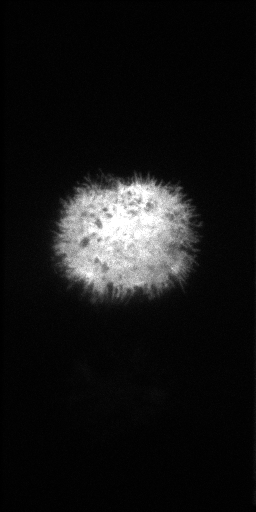

Supplement: Supplementary file 8 — Source Data Fig. 5 [file 44319_2023_11_MOESM8_ESM.zip › FIG 5/Figure 5C/Fig 5C. E-Syt1&2 DKO Jurkat cell activated conditions movie 4- TIRF/movie 4_E-Syt1&2 DKO Jurkat activated t=100.tif]

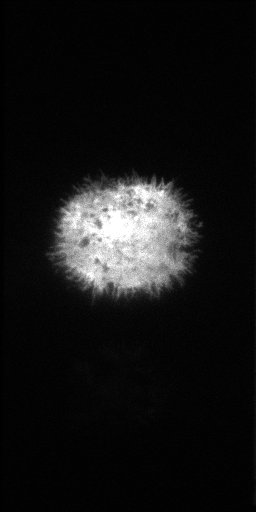

Supplement: Supplementary file 8 — Source Data Fig. 5 [file 44319_2023_11_MOESM8_ESM.zip › FIG 5/Figure 5C/Fig 5C. E-Syt1&2 DKO Jurkat cell activated conditions movie 4- TIRF/movie 4_E-Syt1&2 DKO Jurkat activated t=200.jpg]

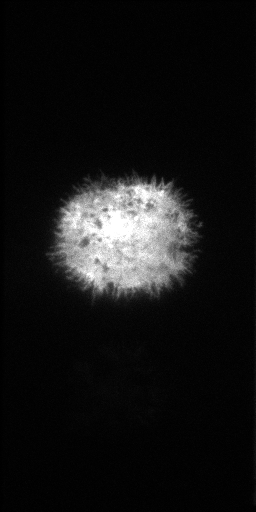

Supplement: Supplementary file 8 — Source Data Fig. 5 [file 44319_2023_11_MOESM8_ESM.zip › FIG 5/Figure 5C/Fig 5C. E-Syt1&2 DKO Jurkat cell activated conditions movie 4- TIRF/movie 4_E-Syt1&2 DKO Jurkat activated t=200.tif]

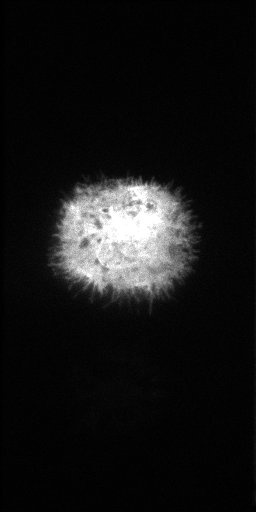

Supplement: Supplementary file 8 — Source Data Fig. 5 [file 44319_2023_11_MOESM8_ESM.zip › FIG 5/Figure 5C/Fig 5C. E-Syt1&2 DKO Jurkat cell activated conditions movie 4- TIRF/movie 4_E-Syt1&2 DKO Jurkat activated t=300.jpg]

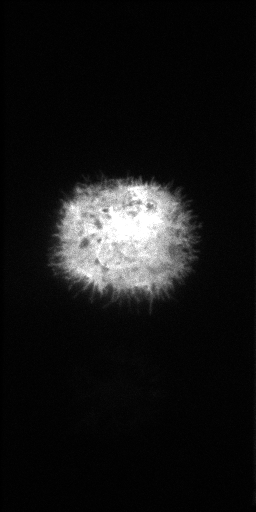

Supplement: Supplementary file 8 — Source Data Fig. 5 [file 44319_2023_11_MOESM8_ESM.zip › FIG 5/Figure 5C/Fig 5C. E-Syt1&2 DKO Jurkat cell activated conditions movie 4- TIRF/movie 4_E-Syt1&2 DKO Jurkat activated t=300.tif]

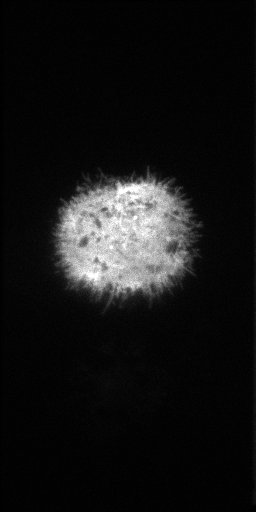

Supplement: Supplementary file 8 — Source Data Fig. 5 [file 44319_2023_11_MOESM8_ESM.zip › FIG 5/Figure 5C/Fig 5C. E-Syt1&2 DKO Jurkat cell activated conditions movie 4- TIRF/movie 4_E-Syt1&2 DKO Jurkat activated t=0.jpg]

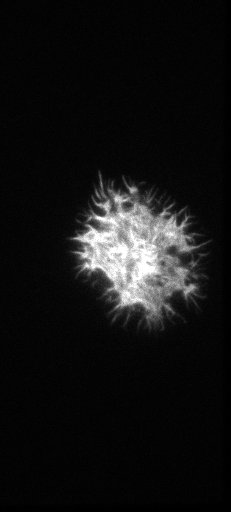

Supplement: Supplementary file 8 — Source Data Fig. 5 [file 44319_2023_11_MOESM8_ESM.zip › FIG 5/Figure 5C/Fig 5C. WT Jurkat activated state movie 1- TIRF /movie 1_ WT Jurkat activated t=200.jpg]

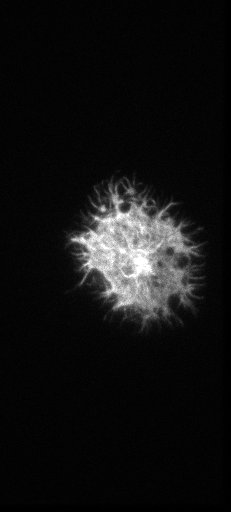

Supplement: Supplementary file 8 — Source Data Fig. 5 [file 44319_2023_11_MOESM8_ESM.zip › FIG 5/Figure 5C/Fig 5C. WT Jurkat activated state movie 1- TIRF /movie 1 _ WT Jurkat activated t=100.jpg]

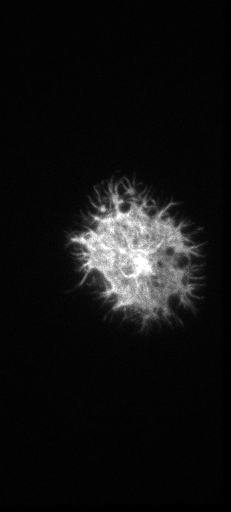

Supplement: Supplementary file 8 — Source Data Fig. 5 [file 44319_2023_11_MOESM8_ESM.zip › FIG 5/Figure 5C/Fig 5C. WT Jurkat activated state movie 1- TIRF /movie 1 _ WT Jurkat activated t=100.tif]

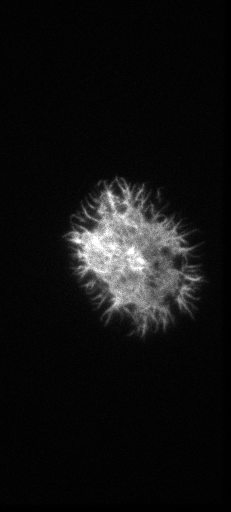

Supplement: Supplementary file 8 — Source Data Fig. 5 [file 44319_2023_11_MOESM8_ESM.zip › FIG 5/Figure 5C/Fig 5C. WT Jurkat activated state movie 1- TIRF /movie 1 _ WT Jurkat activated t=0 .tif]

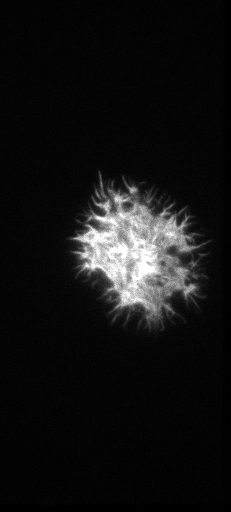

Supplement: Supplementary file 8 — Source Data Fig. 5 [file 44319_2023_11_MOESM8_ESM.zip › FIG 5/Figure 5C/Fig 5C. WT Jurkat activated state movie 1- TIRF /movie 1_ WT Jurkat activated t=200.tif]

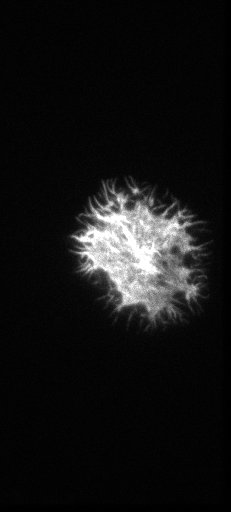

Supplement: Supplementary file 8 — Source Data Fig. 5 [file 44319_2023_11_MOESM8_ESM.zip › FIG 5/Figure 5C/Fig 5C. WT Jurkat activated state movie 1- TIRF /movie 1_ WT Jurkat activated t=300.jpg]

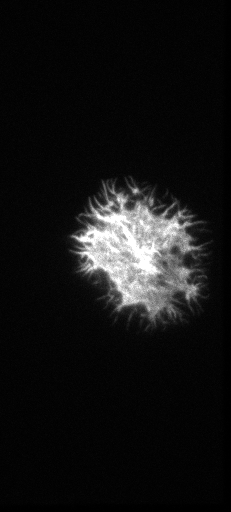

Supplement: Supplementary file 8 — Source Data Fig. 5 [file 44319_2023_11_MOESM8_ESM.zip › FIG 5/Figure 5C/Fig 5C. WT Jurkat activated state movie 1- TIRF /movie 1_ WT Jurkat activated t=300.tif]

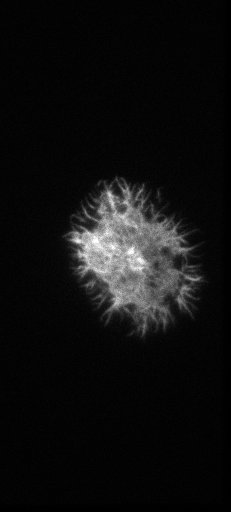

Supplement: Supplementary file 8 — Source Data Fig. 5 [file 44319_2023_11_MOESM8_ESM.zip › FIG 5/Figure 5C/Fig 5C. WT Jurkat activated state movie 1- TIRF /movie 1 _ WT Jurkat activated t=0 .jpg]

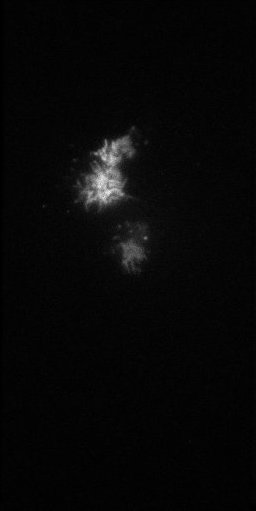

Supplement: Supplementary file 8 — Source Data Fig. 5 [file 44319_2023_11_MOESM8_ESM.zip › FIG 5/Figure 5A/Fig 5A. E-Syt2 KO Jurkat cell resting condition- movie 2 TIRF/movie 2 _ E-Syt2 KO Jurkat resting T-0 .jpg]

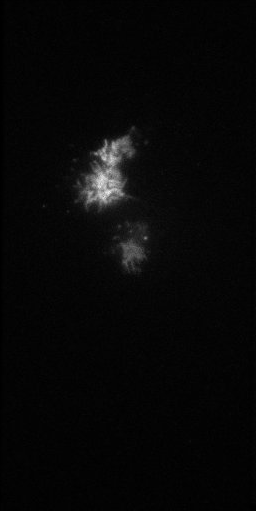

Supplement: Supplementary file 8 — Source Data Fig. 5 [file 44319_2023_11_MOESM8_ESM.zip › FIG 5/Figure 5A/Fig 5A. E-Syt2 KO Jurkat cell resting condition- movie 2 TIRF/movie 2 _ E-Syt2 KO Jurkat resting T-0.tif]

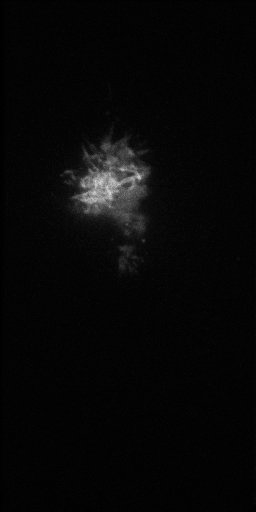

Supplement: Supplementary file 8 — Source Data Fig. 5 [file 44319_2023_11_MOESM8_ESM.zip › FIG 5/Figure 5A/Fig 5A. E-Syt2 KO Jurkat cell resting condition- movie 2 TIRF/movie 2_E-Syt2 KO Jurkat resting T-200 s .tif]

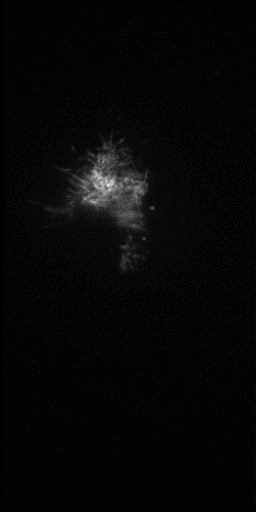

Supplement: Supplementary file 8 — Source Data Fig. 5 [file 44319_2023_11_MOESM8_ESM.zip › FIG 5/Figure 5A/Fig 5A. E-Syt2 KO Jurkat cell resting condition- movie 2 TIRF/movie 2_E-Syt2 KO Jurkat resting T-300 s .tif]

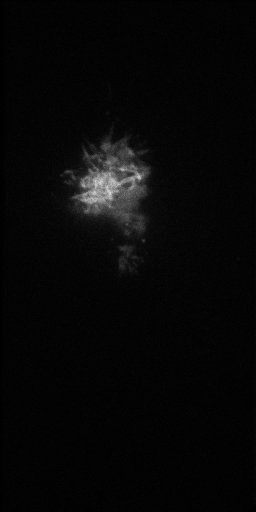

Supplement: Supplementary file 8 — Source Data Fig. 5 [file 44319_2023_11_MOESM8_ESM.zip › FIG 5/Figure 5A/Fig 5A. E-Syt2 KO Jurkat cell resting condition- movie 2 TIRF/movie 2_E-Syt2 KO Jurkat resting T-200 s .jpg]

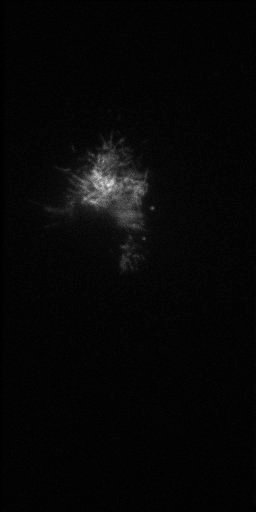

Supplement: Supplementary file 8 — Source Data Fig. 5 [file 44319_2023_11_MOESM8_ESM.zip › FIG 5/Figure 5A/Fig 5A. E-Syt2 KO Jurkat cell resting condition- movie 2 TIRF/movie 2_E-Syt2 KO Jurkat resting T-300 s.jpg]

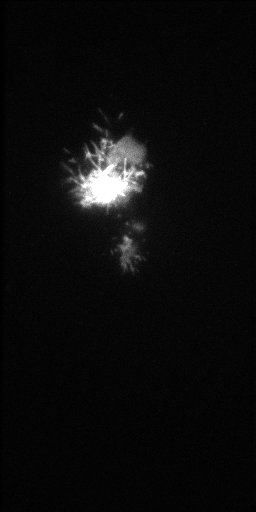

Supplement: Supplementary file 8 — Source Data Fig. 5 [file 44319_2023_11_MOESM8_ESM.zip › FIG 5/Figure 5A/Fig 5A. E-Syt2 KO Jurkat cell resting condition- movie 2 TIRF/movie 2_ E-Syt2 KO Jurkat resting T-100 s.jpg]

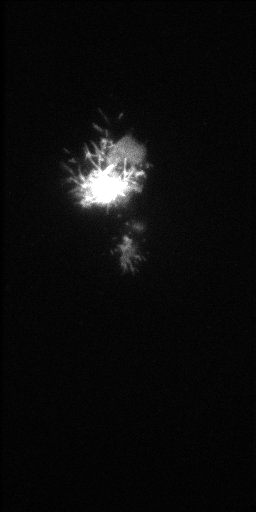

Supplement: Supplementary file 8 — Source Data Fig. 5 [file 44319_2023_11_MOESM8_ESM.zip › FIG 5/Figure 5A/Fig 5A. E-Syt2 KO Jurkat cell resting condition- movie 2 TIRF/movie 2_ E-Syt2 KO Jurkat resting T-100 s.tif]

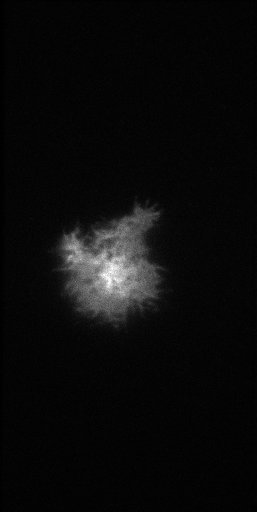

Supplement: Supplementary file 8 — Source Data Fig. 5 [file 44319_2023_11_MOESM8_ESM.zip › FIG 5/Figure 5A/Fig 5A. WT Jurkat resting state movie 9- TIRF /movie 9_WT Jurkat resting T-300.jpg]

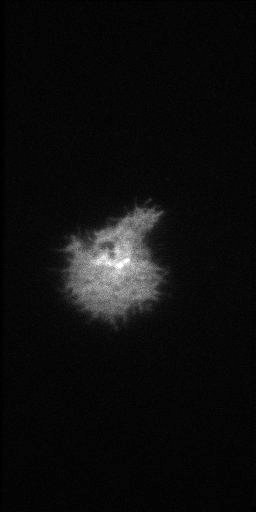

Supplement: Supplementary file 8 — Source Data Fig. 5 [file 44319_2023_11_MOESM8_ESM.zip › FIG 5/Figure 5A/Fig 5A. WT Jurkat resting state movie 9- TIRF /movie 9_WT Jurkat resting T-0 .jpg]
