# Supplementary figures and images for: Extended-Synaptotagmin-1 and -2 control T cell signaling and function (part 2 of 2)
Source: EMBO Rep. 2023 Dec 19;25(1):286–303. doi: 10.1038/s44319-023-00011-7 (PMC10897422; doi:10.1038/s44319-023-00011-7)

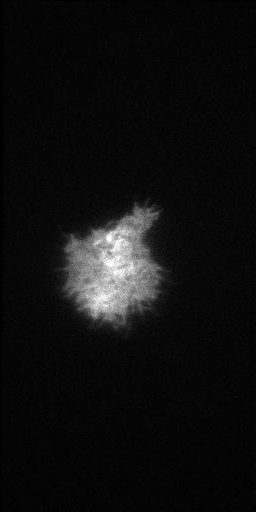

Supplement: Supplementary file 8 — Source Data Fig. 5 [file 44319_2023_11_MOESM8_ESM.zip › FIG 5/Figure 5A/Fig 5A. WT Jurkat resting state movie 9- TIRF /movie 9_WT Jurkat resting T-200.tif]

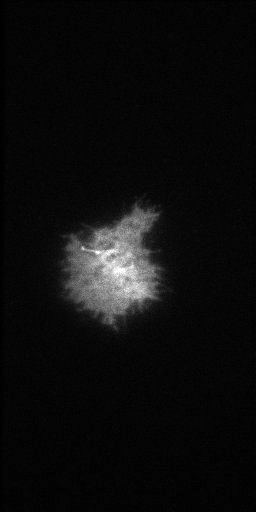

Supplement: Supplementary file 8 — Source Data Fig. 5 [file 44319_2023_11_MOESM8_ESM.zip › FIG 5/Figure 5A/Fig 5A. WT Jurkat resting state movie 9- TIRF /movie 9_WT Jurkat resting T-100 .tif]

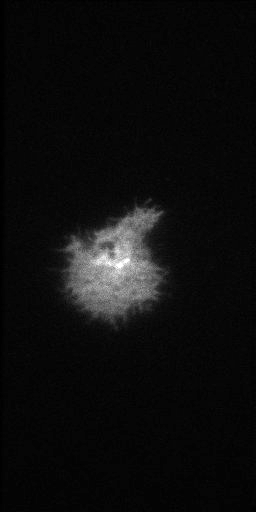

Supplement: Supplementary file 8 — Source Data Fig. 5 [file 44319_2023_11_MOESM8_ESM.zip › FIG 5/Figure 5A/Fig 5A. WT Jurkat resting state movie 9- TIRF /movie 9_WT Jurkat restingT-0 .tif]

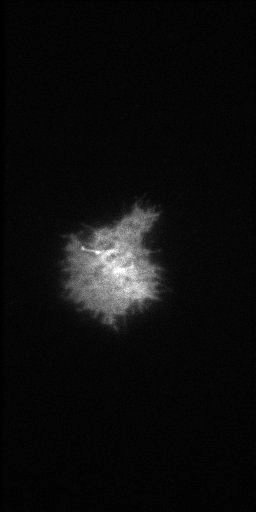

Supplement: Supplementary file 8 — Source Data Fig. 5 [file 44319_2023_11_MOESM8_ESM.zip › FIG 5/Figure 5A/Fig 5A. WT Jurkat resting state movie 9- TIRF /movie 9_WT Jurkat resting T-100 .jpg]

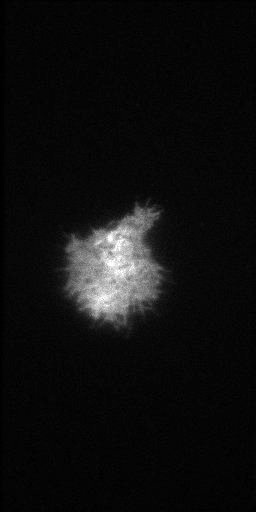

Supplement: Supplementary file 8 — Source Data Fig. 5 [file 44319_2023_11_MOESM8_ESM.zip › FIG 5/Figure 5A/Fig 5A. WT Jurkat resting state movie 9- TIRF /movie 9_WT Jurkat resting T-200.jpg]

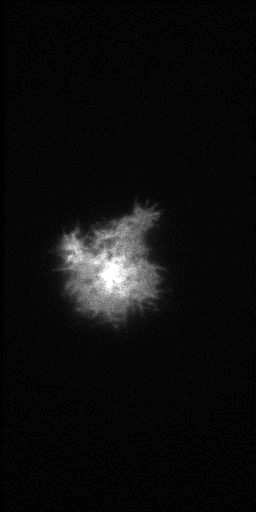

Supplement: Supplementary file 8 — Source Data Fig. 5 [file 44319_2023_11_MOESM8_ESM.zip › FIG 5/Figure 5A/Fig 5A. WT Jurkat resting state movie 9- TIRF /movie 9_WT Jurkat resting T-300.tif]

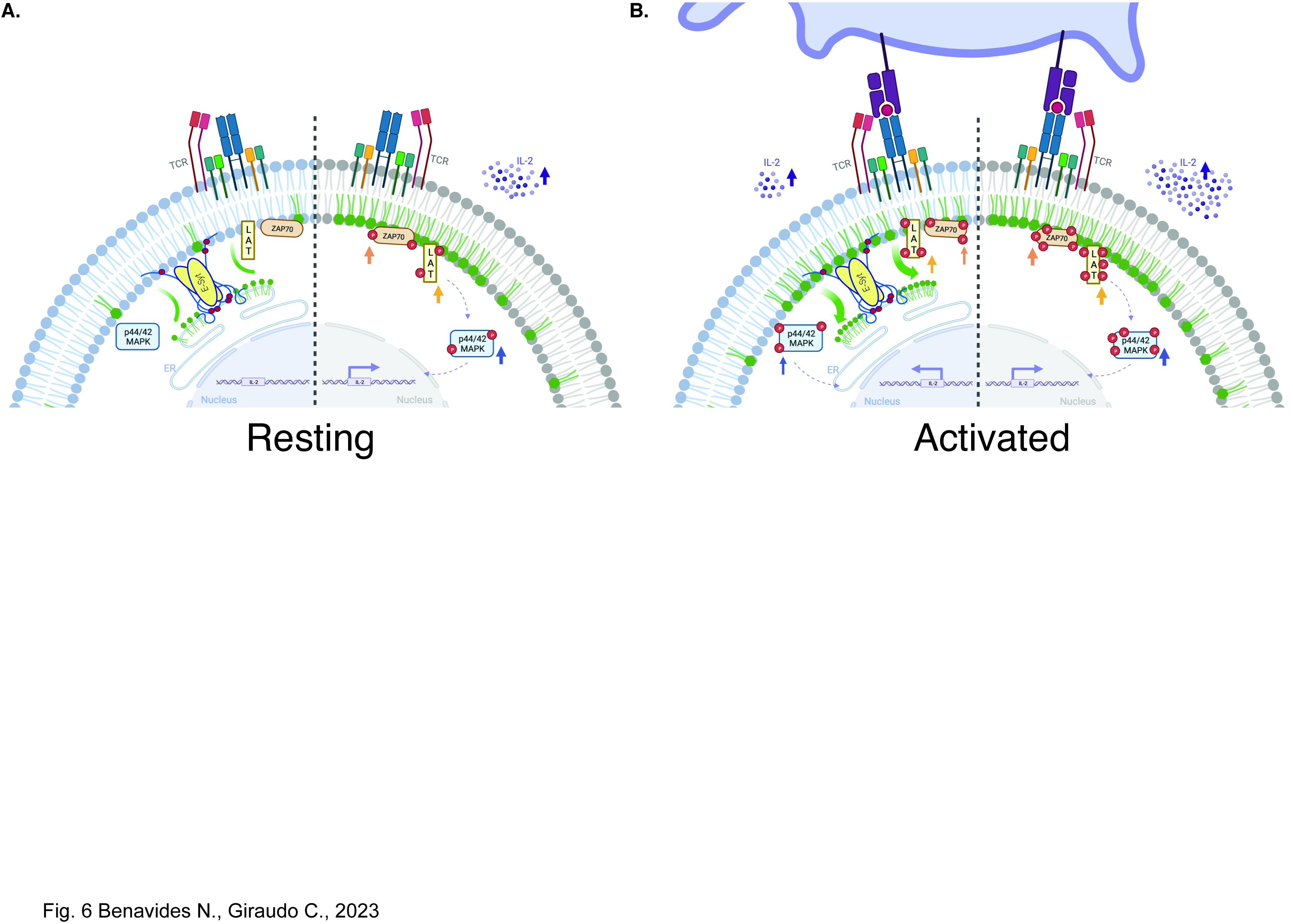

Supplement: Supplementary file 9 — Source Data Fig. 6 [file 44319_2023_11_MOESM9_ESM.zip › FIG 6/FIgure 6. E-Syt proteins modulate signaling and functionality of T cells via plasma membrane DAG levels.tif]

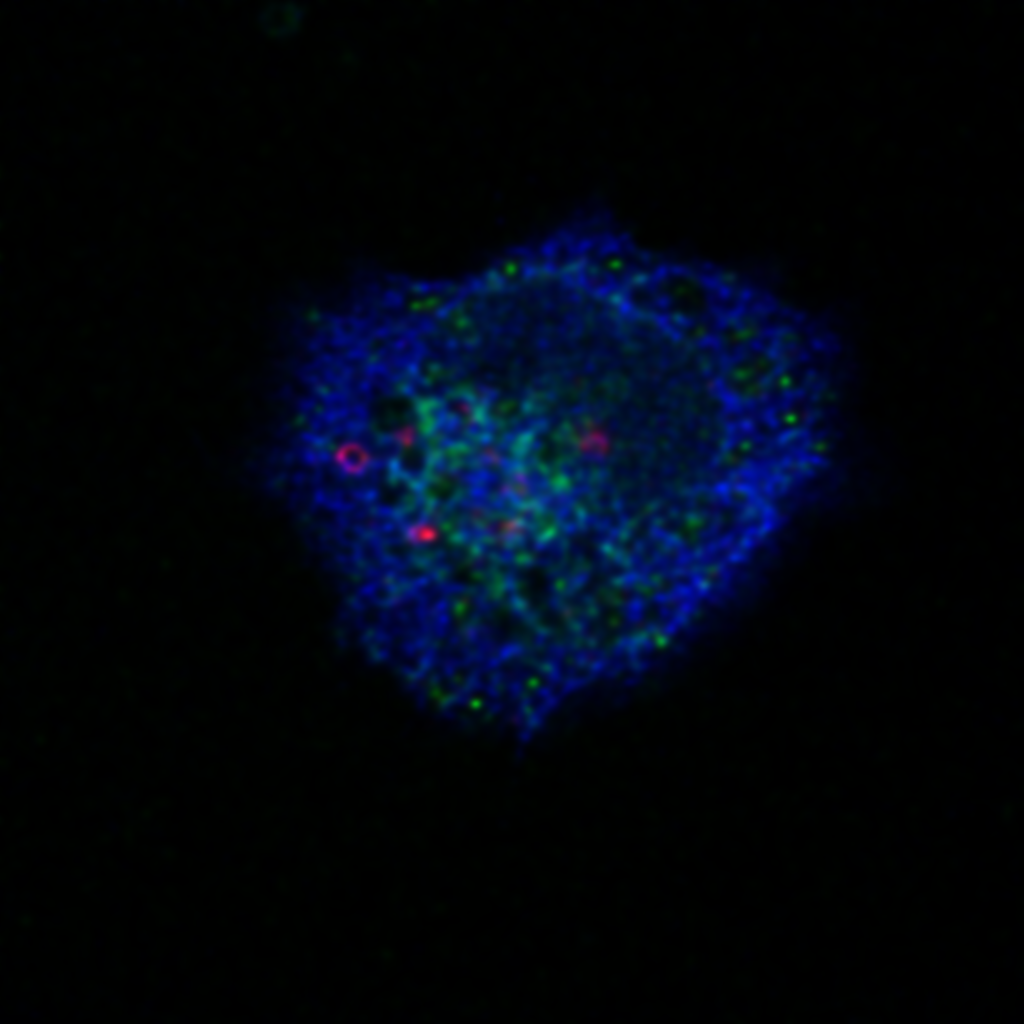

Supplement: Supplementary file 10 — Source Data EV Figs. 1-5 [file 44319_2023_11_MOESM10_ESM.zip › Source Data - External View Figures /Fig EV2 /Figure EV 2A/2022-12-16-WT-Jurkat-GFP-DAG-mCheery-ESYT1-Cy5-EEA1-Resting004_decon.tif]

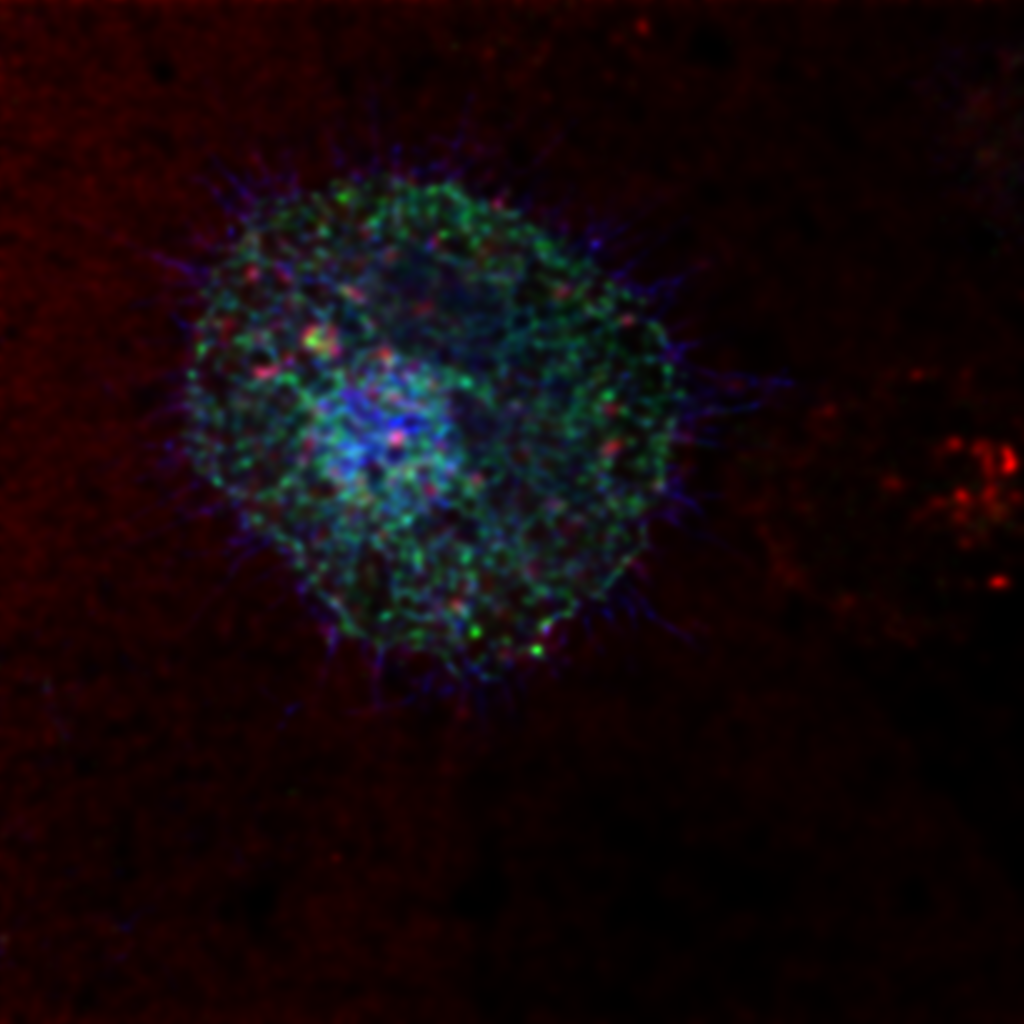

Supplement: Supplementary file 10 — Source Data EV Figs. 1-5 [file 44319_2023_11_MOESM10_ESM.zip › Source Data - External View Figures /Fig EV2 /Figure EV 2C/2023-01-23-WT-Jurkat-GFP-DAG-mCherryESyt1-CY5-EEA1-Act019_decon.tif]

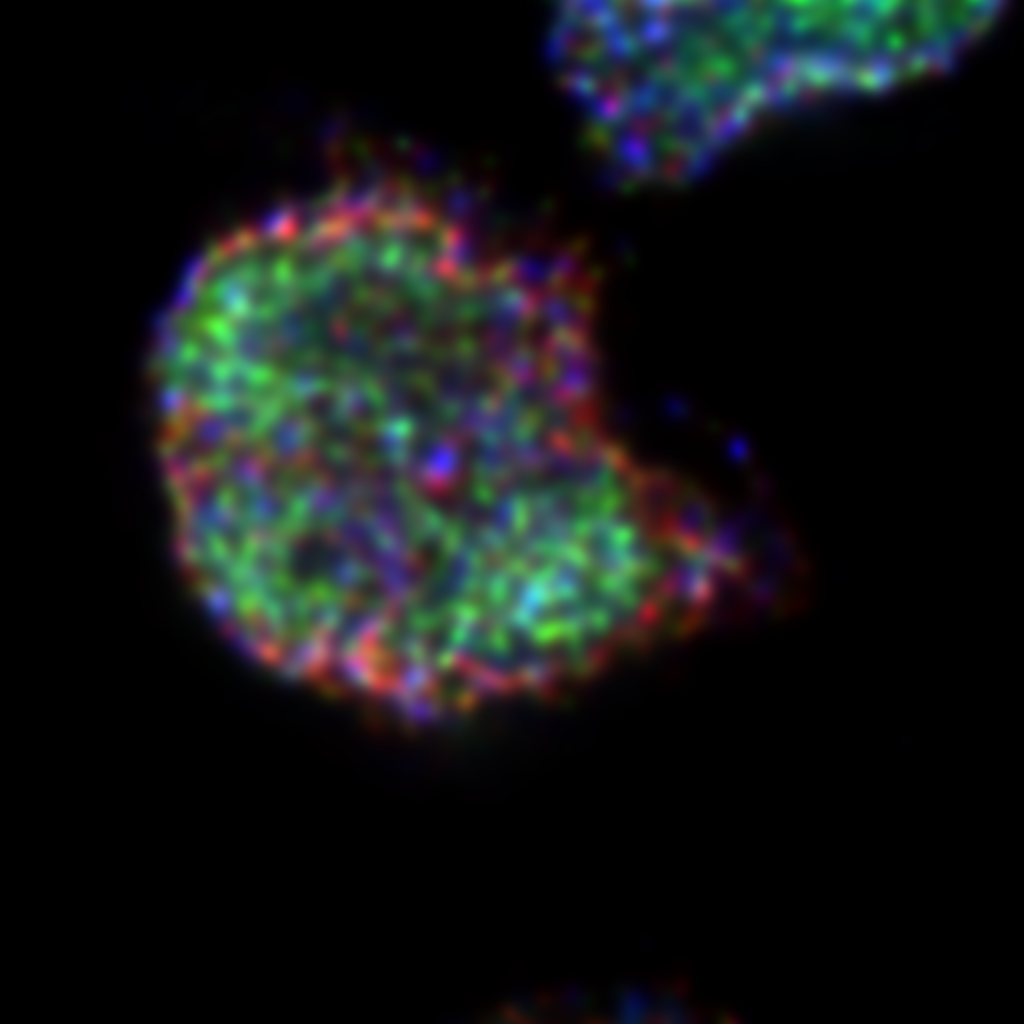

Supplement: Supplementary file 10 — Source Data EV Figs. 1-5 [file 44319_2023_11_MOESM10_ESM.zip › Source Data - External View Figures /Fig EV3/Figure EV 3A/2023-08-05-WT-Jrukat-Cy3-ZAP70-Cy5-pZAP70-Resting012_decon.tif]

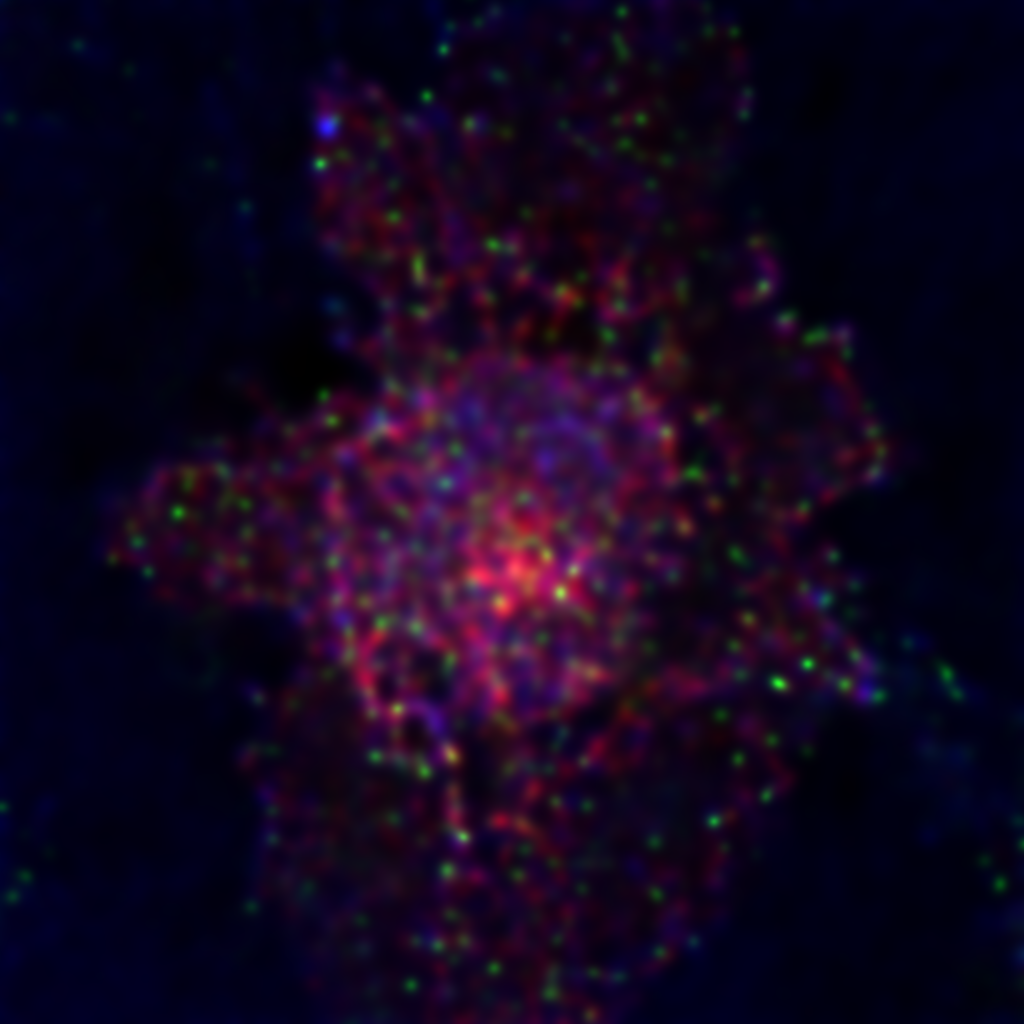

Supplement: Supplementary file 10 — Source Data EV Figs. 1-5 [file 44319_2023_11_MOESM10_ESM.zip › Source Data - External View Figures /Fig EV3/Figure EV 3A/2023-07-13-ESyt2KO-Jurkat-GFP-DAG-Cy3-pZAP70-Cy5-ZAP70-Activated012_decon.tif]

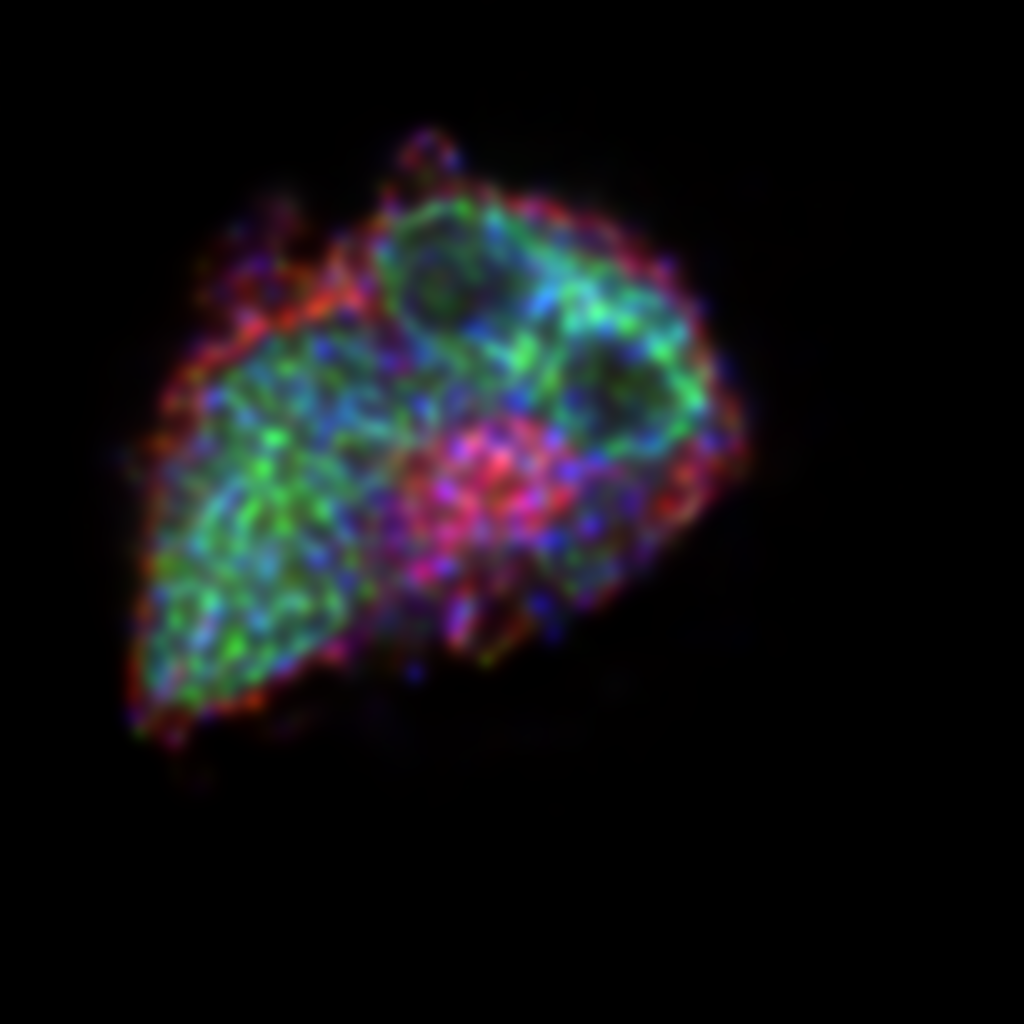

Supplement: Supplementary file 10 — Source Data EV Figs. 1-5 [file 44319_2023_11_MOESM10_ESM.zip › Source Data - External View Figures /Fig EV3/Figure EV 3A/2023-08-05-WT-Jrukat-Cy3-ZAP70-Cy5-pZAP70-Activated004_decon.tif]

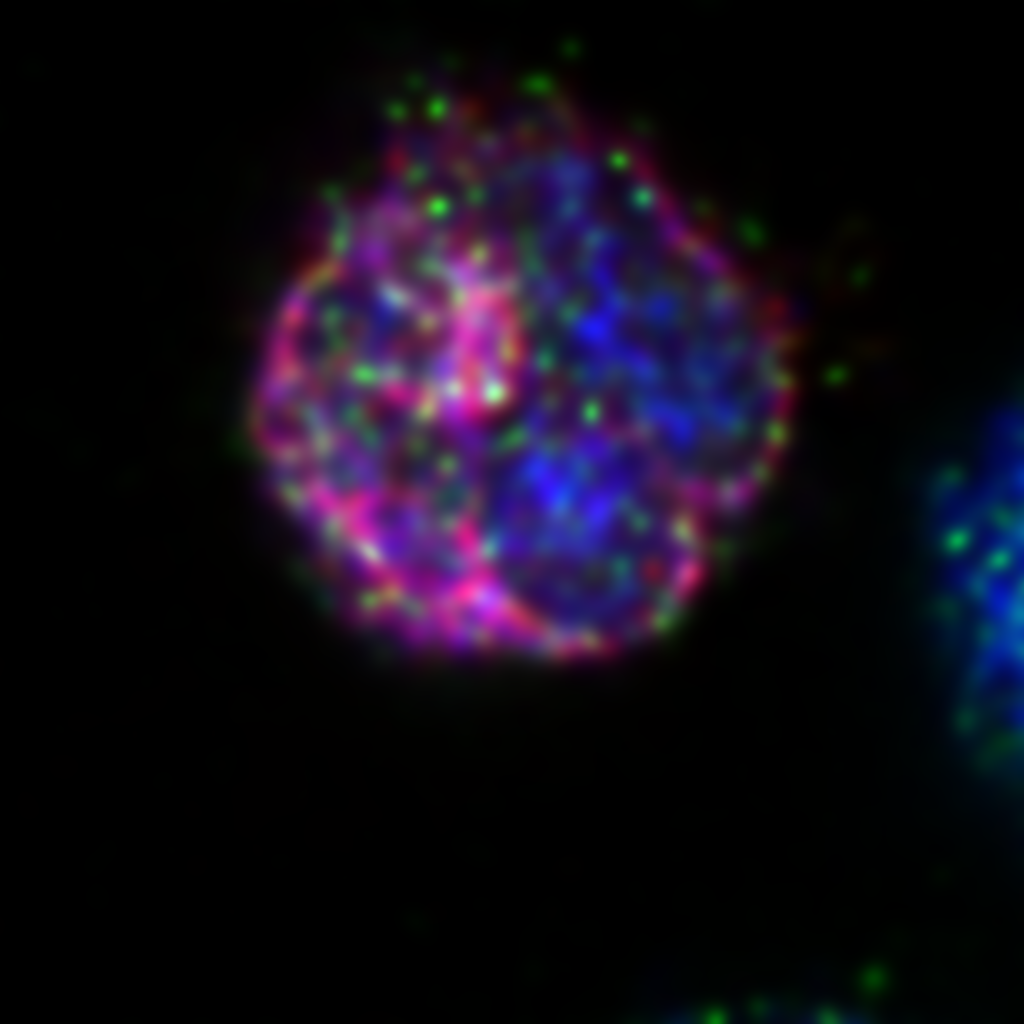

Supplement: Supplementary file 10 — Source Data EV Figs. 1-5 [file 44319_2023_11_MOESM10_ESM.zip › Source Data - External View Figures /Fig EV3/Figure EV 3A/2023-07-07-ESyt2KO-Jurkat-GFP-DAG-Cy3-pZAP70-Cy5-ZAP70-Resting017_decon.tif]
