# Supplementary material for: Extended-Synaptotagmin-1 and -2 control T cell signaling and function
Source: EMBO Rep. 2023 Dec 19;25(1):286–303. doi: 10.1038/s44319-023-00011-7 (PMC10897422; doi:10.1038/s44319-023-00011-7)
Supplement: Supplementary file 11 — Expanded View Figures PDF [file 44319_2023_11_MOESM11_ESM.pdf]

Expanded View Figures

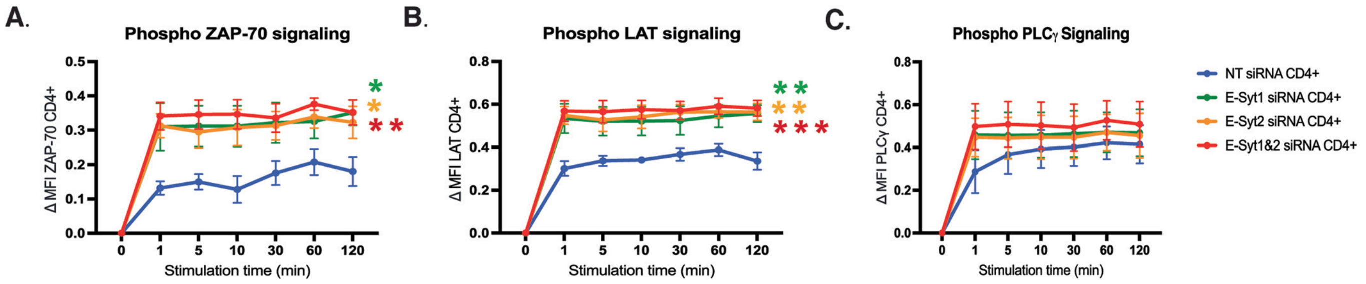

Figure EV1. E-Syt1 and -2 knockdown in primary CD4⁺ T cells enhances TCR signaling kinetics.

(**A–C**) Flow cytometry analysis of CD3 + CD4+ gated T cells from human PBMCs using signaling events measured by phosphorylation levels of early TCR signaling proteins ZAP70 at Y292 (**A**) LAT at Y226 (**B**) and PLC-γ1 at Y783 (**C**) upon stimulation with anti-CD3 and CD28 antibody-coated beads for indicated times. Plots show the change in median fluorescence intensity (MFI) between the indicated time of stimulation and $t = 0$. Plots are a mean ± SEM of at least three independent experiments. Significance was determined by two-way ANOVA with Bonferroni's multiple comparisons test against control NT siRNA CD4⁺ T cells (*$P < 0.0332$, **$P < 0.0021$, ***$P < 0.0002$). Source data are available online for this figure.

© The Author(s)

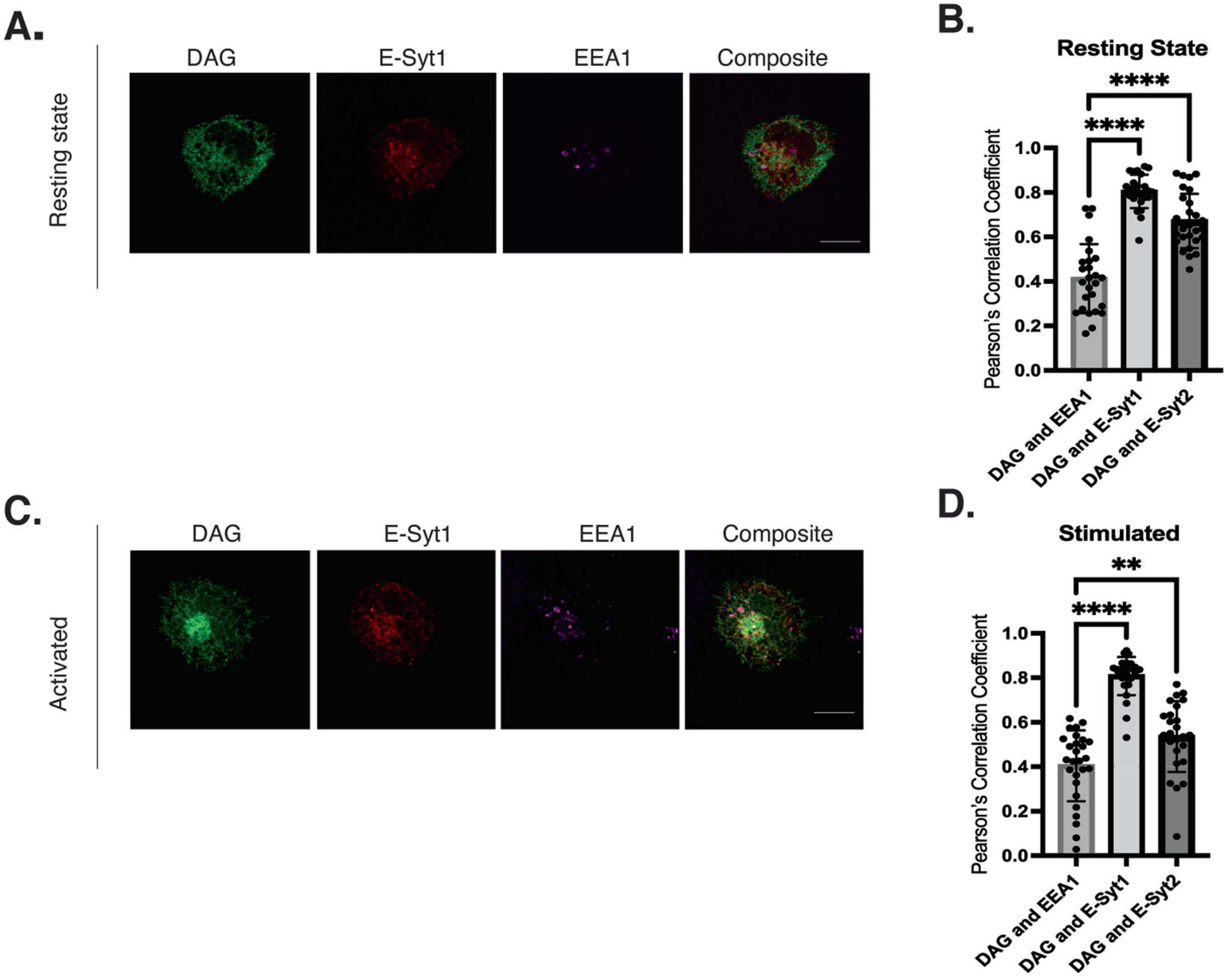

Figure EV2. E-Syt1 and E-Syt2 colocalize with DAG in Jurkat cells.

(**A**) Representative confocal images of WT-Jurkat cells transfected with GFP-PKCg-C1 probe (DAG) and stained with anti-E-Syt1 (red) and anti-EEA1 (Early Endosome Antigen 1, magenta) attached onto poly-lysine treated glass coverslips (resting state). Scale bars: 5 µm. (**B**) Pearson's correlation coefficient analysis of DAG-E-Syt1, DAG-E-Syt2, and DAG-EEA1 colocalization of all cell lines in resting state (DAG-EEA1 $n = 26$ cell, DAG-E-Syt1 $n = 26$ cells, DAG-E-Syt2 $n = 28$ cells) Plots are the mean ± SEM of at least three independent experiments. Significance was determined by one-way ANOVA with Bonferroni's multiple comparisons test against control WT-Jurkat cells (****$P < 0.0001$). (**C**) Representative confocal images of WT-Jurkat cells transfected with GFP-PKCg-C1 probe (DAG) and stained with anti-E-Syt1 (red) and anti-EEA1 (Early Endosome Antigen 1, magenta) attached onto poly-lysine treated glass coverslips plus anti-CD3 and -CD28 antibodies treated glass coverslips (activated state). Scale bars: 5 µm. (**D**) Pearson's correlation coefficient analysis of DAG-E-Syt1, DAG-E-Syt2, and DAG-EEA1 colocalization of all cell lines in stimulated conditions (DAG-EEA1 $n = 27$ cell, DAG-E-Syt1 $n = 27$ cells, DAG-E-Syt2 $n = 25$ cells). Plots are the mean ± SEM of at least three independent experiments. Significance was determined by one-way ANOVA with Bonferroni's multiple comparisons test against control WT-Jurkat cells (**$P < 0.0021$, ****$P < 0.0001$). Source data are available online for this figure.

© The Author(s)

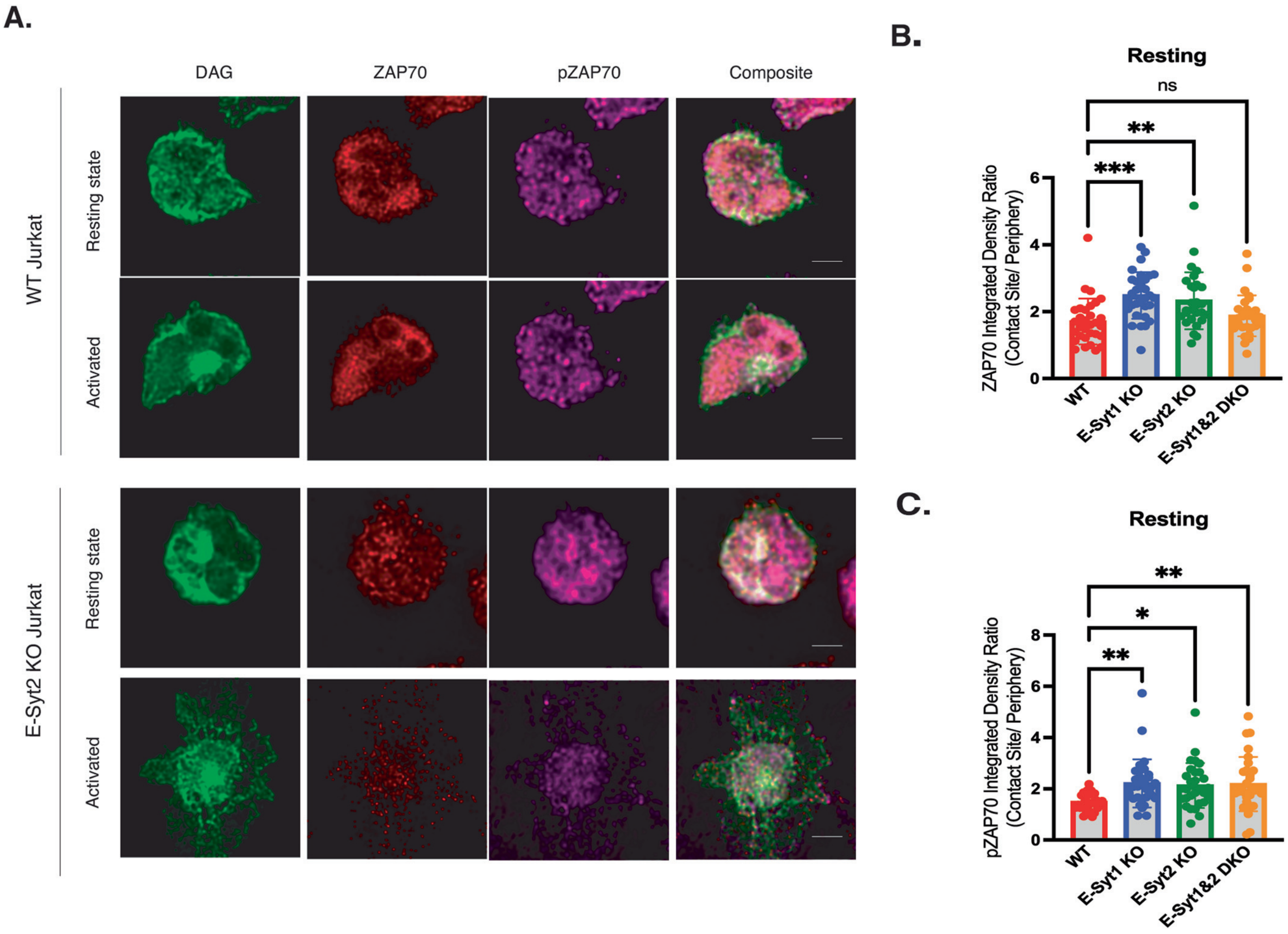

Figure EV3. E-Syt proteins modulate p-ZAP70 and total-ZAP70 levels in Jurkat cells in resting conditions.

(**A**) Representative WT and E-Syt2 KO Jurkat cells transfected with GFP-PKCg-C1 probe (DAG) and stained with anti-ZAP70 and anti-p-ZAP70 antibodies as signaling markers in resting and activated states. Scale bars: 5 µm. (**B**, **C**) Quantification of the ratio of the integrated fluorescent density of ZAP70 (**B**) p-ZAP70 (**C**) between the contact site area (DAG-rich area) and the cell periphery (low DAG signal). Data are shown as mean ± SEM of at least three independent experiments (WT $n = 31$ cells; E-Syt1 KO $n = 31$ cells; E-Syt2 KO $n = 29$ cells; E-Syt1&2 KO $n = 30$ cells). Significance was determined by one-way ANOVA with Bonferroni's multiple comparisons test against control WT Jurkat (*$P < 0.0332$, **$P < 0.0021$, ***$P < 0.0002$). Source data are available online for this figure.

 © The Author(s)

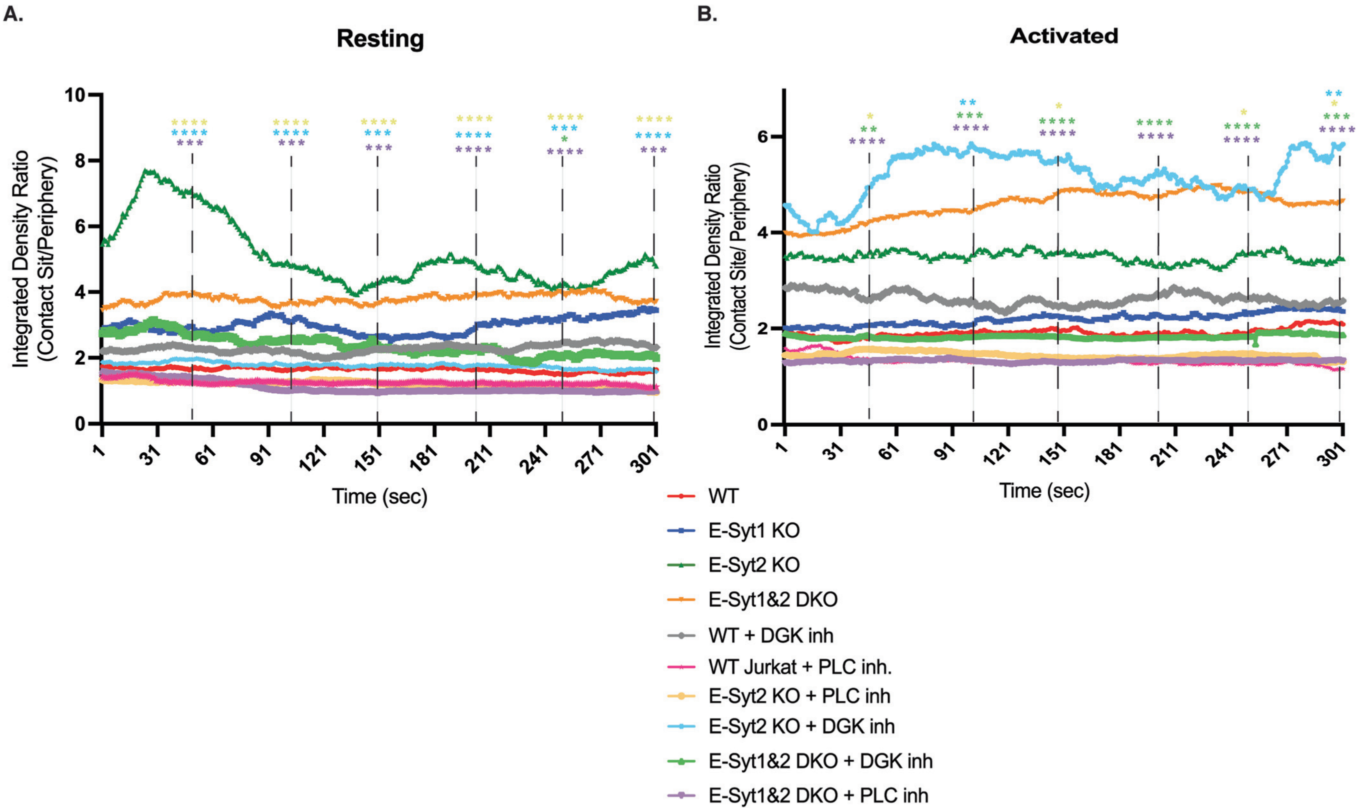

Figure EV4. DAG production at the PM of Jurkat cells requires PLCγ activity.

(**A**, **B**) Quantification from TIRF movies of WT and E-Syt KO cell lines transfected with GFP-C1ab for PM DAG detection treated over night with 5 μM PLCγ inhibitor or 300 μM DGK inhibitor in resting (**A**) or activated conditions (**B**). Quantification of the ratio of the integrated fluorescent density of DAG-rich areas and the periphery. Data are shown as mean ± SEM of at least three independent experiments (WT $n = 8$ cells; E-Syt1 KO $n = 8$ cells; E-Syt2 KO $n = 8$ cells, E-Syt1&2 KO $n = 8$ cells). Significance was determined by two-way ANOVA with Bonferroni's multiple comparisons test against the respective non-treated cell line control (*$P < 0.0332$, **$P < 0.0021$, ***$P < 0.0002$, ****$P < 0.0001$). For clarity, we only showed statistically significant points at the indicated times (50, 100, 150, 200, 250, 300 s). Source data are available online for this figure.

© The Author(s)

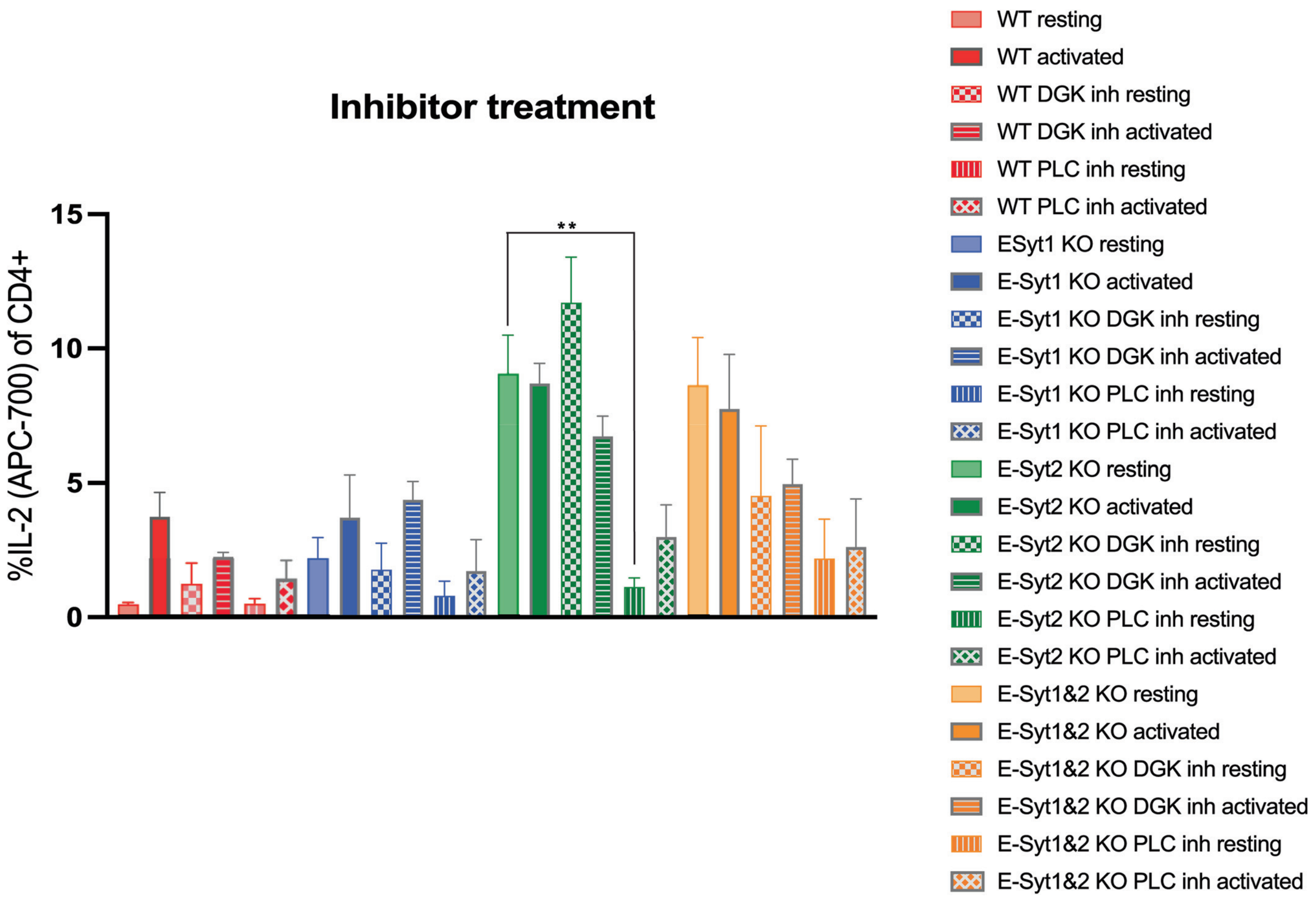

Figure EV5. Treatment of Jurkat cells with PLCγ inhibitor decreases IL-2 production regardless of E-Syt proteins.

Intracellular staining of IL-2 measured by flow cytometry in WT and E-Syt KO Jurkat cell lines at resting state (faded colored bars). Jurkat cell lines stimulated with anti-CD3 and -CD28 coated beads for 12 h (solid colored bars). Jurkat cell lines were treated 5 h prior with 5 μM PLCγ inhibitor or 300 μM before starting the stimulation process. Figure legend indicates cell line, inhibitor treatment, and activation status. The data shown represent the mean ± SEM of at least four independent experiments. Significance was determined by one-way ANOVA with Bonferroni's multiple comparisons test against the respective non-treated cell line control (**$P < 0.0021$). Source data are available online for this figure.

 © The Author(s)
