# Supplementary material for: Extended-Synaptotagmin-1 and -2 control T cell signaling and function
Source: EMBO Rep. 2023 Dec 19;25(1):286–303. doi: 10.1038/s44319-023-00011-7 (PMC10897422; doi:10.1038/s44319-023-00011-7)
Supplement: Supplementary file 1 — Appendix and Sup Figs [file 44319_2023_11_MOESM1_ESM.docx]

**Appendix**

**Table of Contents**

Appendix Figure S1 …………………………………………………………………….………2

Appendix Figure S2 …………………………………………………………………….………3

Appendix Figure S3 ……………………………………………………………….……………5

Appendix Figure S4 ……………………………………………………………………….……6

Appendix Figure S5 ………………………………………………………………………….…8

Appendix Figure S6 ………………………………………………………………………..…10

Appendix Figure S7 …………………………………………………………………..……….11

Appendix Figure S8 …………………………………………………………………..………12

Appendix Figure S1

**Expression of E-Syt proteins in human immune cells**

**A.** Western blot analysis of the expression of E-Syt1, E-Syt2, E-Syt3, and actin in human Peripheral Blood Mononuclear Cells (PBMCs), purified CD8^+^ T cells, and human cell lines such as HeLa epithelial cells, Jurkat CD4^+^ T cells, and YTS Natural Killer cells.

**B.** Mass spectrometry results of GFP-STX11 coimmunoprecipitation experiments using lysates from human primary CD8^+^ T cells transfected with either GFP-STX11 or GFP alone and activated with beads coated with anti-CD3 and -CD28. GFP molecules were immunoprecipitated using an anti-GFP antibody and pulled-down products were analyzed by mass spectrometry. MS/MS counts were compared between the GFP alone controls versus GFP-STX11. Bayes Factor was used as a statistical parameter to normalize the number of peptides by the protein size.

Appendix Figure S2

**Downregulation of E-Syt1&2 increases phospho-protein signaling events in Jurkat cells**

**A**. Representative Western blot showing levels of p- PLCγ1, p44/42 MAPK, p-LAT, and actin from cell lysates of NT siRNA and E-Syt1&2 siRNA treated Jurkat cells stimulated with anti-CD3 and CD28 antibody-coated beads for indicated times. Total PLCγ1, MAPK, LAT, and β-actin were used as loading controls.

**B, C, D.** Densitometry graphs showing normalized phosphorylation of LAT **(B.)**, PLCγ1 **(C.)**, and p44/42 MAPK **(D.)** Plots are a mean ±SEM of at least 3 independent experiments. Significance was determined by two-way aNOVA with Bonferroni’s multiple comparisons test against control NT siRNA Jurkat (*p<0.0332).

**E, F, G.** Raw mean fluorescence intensity (MFI) values at stimulation time t=0 of p-ZAP70 **(E.),** p-LAT **(F.),** and p**-**PLCγ **(G.)** of three independent experiments. Plots show the change in median fluorescence intensity (MFI) between the indicated time of stimulation and t=0. Plots are a mean ±SEM of at least 3 independent experiments. Significance was determined by two-way aNOVA with Bonferroni’s multiple comparisons test against control NT siRNA Jurkat.

Appendix Figure S3

**Absence of E-Syt2 increases IL-2 MFI in Jurkat cells**

**A, B.** Mean fluorescence intensity (MFI) of intracellular staining of IL-2 measured by flow cytometry in WT control and E-Syt KO Jurkat cell lines in resting state **(A.)** stimulated with anti-CD3 and **-**CD28 coated beads **(B.)** The data shown represent the mean ±SEM of at least three independent experiments. Significance was determined by one-way aNOVA with Bonferroni’s multiple comparisons test against control WT Jurkat.

Appendix Figure S4

**E-Syt1 and E-Syt2 subcellular localization in comparison to ER, PM, and nucleus**

**A, B.** WT Jurkat cells were transfected with either EGFP-ESyt1 **(A.)** or EGFP-Esyt2 **(B.)** and stained for KDEL- receptor (ER marker) and/or CellMask for PM or DRAQ5 for the nucleus. Each set of markers is displayed in resting or activated conditions**.**

Appendix Figure S5

**E-Syt proteins modulate DAG and CD4 colocalization in Jurkat cells in resting conditions**

### A. WT, E-Syt1 KO, E-Syt2 KO, and E-Syt1&2 DKO Jurkat cells transfected with GFP-PKCg-C1 probe (DAG) and stained with anti-CD4 and anti-LAT antibodies as synaptic markers in resting and activated states.

### B,C Pearson’s correlation coefficient analysis of DAG and CD4 of E-Syt KO and Jurkat WT cell lines in resting state (WT Jurkat n=38 cells, E-Syt1KO Jurkat n=39 cells, E-Syt2KO Jurkat n=38 cells, E-Syt1&2DKO Jurkat n= 40 cells) (B.) and activated state (WT Jurkat n=44 cell, E-Syt1KO Jurkat n=41 cells, E-Syt2KO Jurkat n=38cells, E-Syt1&2DKO Jurkat n=43 cells) (C.). Plots are a mean ±SEM of at least three independent experiments. Significance was determined by one-way aNOVA with Bonferroni’s multiple comparisons test against control WT Jurkat cells (**p<0.0021, ***p<0.0002).

Appendix Figure S6

**Overexpression of E-Syt1 in Jurkat cells does not impact DAG distribution**

**A, B.** WT Jurkat cells were transfected with EGFP-DAG alone or in conjunction of mCherry-Esyt1 and integrated density ratios for DAG were measured at resting state **(A.)** and activated state **(B.)** (20 cells were analyzed per each condition). Plots are a mean ±SEM of at least three independent experiments. Significance was determined by one-way aNOVA with Bonferroni’s multiple comparisons test against control WT Jurkat cells.

Appendix Figure S7

**E-Syt1 and -2 knockdown in PBMCs increases the colocalization between DAG and lytic granule marker CD63**

**A.** Representative images of NT siRNA and E-Syt1&2 siRNA PBMCs were transfected with EGFP-DAG and stained for tubulin and CD63

**B.** Pearson’s correlation coefficient analysis of transfected PBMCs conjugated with P815 Target cells (NT siRNA PBMCs n=**28** cells, E-Syt1 siRNA PBMCs n=**31** cells, E-Syt2 siRNA PBMC n=**34** cells, E-Syt1&2 siRNA PBMCn= **34** cells). Plots are a mean ±SEM of at least three independent experiments. Significance was determined by one-way aNOVA with Bonferroni’s multiple comparisons test against control NT siRNA PBMCs cells (*p<0.0332, **p<0.0021, ***p<0.0002).

Appendix Figure S8

**Absence of E-Syt proteins does not induce apoptosis after T cell stimulation**

WT and E-Syt KO Jurkat cell lines were stained for Annexin V for detection of apoptosis upon stimulated with anti-CD3 and -CD28 coated beads. Cell lines were treated with staurosporine as a control as this induces apoptosis in T cells. Annexin V (APC) detection was performed using flow cytometry. Data are shown as a mean ±SEM of at least three independent experiments. Significance was determined by one-way aNOVA with Bonferroni’s multiple comparisons test against the non-treated WT control cells respective of stimulation status (**p<0.0021).
